# A more objective PD diagnostic model: integrating texture feature markers of cerebellar gray matter and white matter through machine learning

**DOI:** 10.3389/fnagi.2024.1393841

**Published:** 2024-06-07

**Authors:** Yini Chen, Yiwei Qi, Tianbai Li, Andong Lin, Yang Ni, Renwang Pu, Bo Sun

**Affiliations:** ^1^Department of Radiology, The First Affiliated Hospital of Dalian Medical University, Dalian, China; ^2^Liaoning Provincial Key Laboratory for Research on the Pathogenic Mechanisms of Neurological Diseases, The First Affiliated Hospital, Dalian Medical University, Dalian, China; ^3^Department of Neurology, Zhejiang Taizhou Municipal Hospital, Taizhou, Zhejiang, China

**Keywords:** Parkinson's disease, radiomic, machine learning, SHAP, FeAture Explorer

## Abstract

**Objective:**

The purpose of this study is to explore whether machine learning can be used to establish an effective model for the diagnosis of Parkinson's disease (PD) by using texture features extracted from cerebellar gray matter and white matter, so as to identify subtle changes that cannot be observed by the naked eye.

**Method:**

This study involved a data collection period from June 2010 to March 2023, including 374 subjects from two cohorts. The Parkinson's Progression Markers Initiative (PPMI) served as the training set, with control group and PD patients (HC: 102 and PD: 102) from 24 global sites. Our institution's data was utilized as the test set (HC: 91 and PD: 79). Machine learning was employed to establish multiple models for PD diagnosis based on texture features of the cerebellum's gray and white matter. Results underwent evaluation through 5-fold cross-validation analysis, calculating the area under the receiver operating characteristic curve (AUC) for each model. The performance of each model was compared using the Delong test, and the interpretability of the optimized model was further augmented by employing Shapley additive explanations (SHAP).

**Results:**

The AUCs for all pipelines in the validation dataset were compared using FeAture Explorer (FAE) software. Among the models established by Kruskal-Wallis (KW) and logistic regression via Lasso (LRLasso), the AUC was highest using the “one-standard error” rule. 'WM_original_glrlm_GrayLevelNonUniformity' was considered the most stable and predictive feature.

**Conclusion:**

The texture features of cerebellar gray matter and white matter combined with machine learning may have potential value in the diagnosis of Parkinson's disease, in which the heterogeneity of white matter may be a more valuable imaging marker.

## Introduction

Parkinson's disease (PD) is a progressive neurodegenerative disease that is commonly observed in the elderly population and is characterized by significant motor dysfunction, including resting tremors, muscle rigidity, bradykinesia, and balance impairment (Bloem et al., 2021; Lang et al., 2022). As the disease progresses, patients may experience non-motor symptoms such as significant cognitive impairment, emotional fluctuations, sleep irregularities, and autonomic dysfunction (Sun et al., 2019). Although the cause of PD remains elusive, research suggests that genetic predispositions, environmental factors such as certain chemicals and toxins, aging, and other factors play important roles. Currently, treatment for PD primarily focuses on managing symptoms and improving patients' quality of life. The main approaches to treatment include pharmacotherapy, physical therapy, and occasionally surgical procedures (Lang et al., 2022; Cramb et al., 2023).

Medical image texture analysis is a quantification approach for assessing internal patterns and image structures, having demonstrated potential in disease quantitative analysis (Zwanenburg et al., 2020; Fisher et al., 2024). Machine learning, a pivotal tool in medical image texture analysis, offers numerous unique benefits to the medical domain. Notably, machine learning permits efficient processing of large-scale medical image data, enabling swift and accurate extraction and analysis of texture features, thus providing clinicians and researchers with more comprehensive, objective data for precise disease diagnosis and treatment (Sharma et al., 2023). Additionally, machine learning uncovers and learns hidden complex patterns and features within medical images during texture analysis. By harnessing advanced technologies such as deep learning, machine learning can autonomously extract crucial texture information beneficial for disease diagnosis and prediction, thereby enhancing the sensitivity and accuracy of potential disease detection (Yang et al., 2023). Furthermore, machine learning personalizes medical image analysis, tailoring diagnosis and treatment to patients' unique conditions and medical histories, thus escalating treatment precision and reducing needless interventions, ultimately improving medical outcomes (Chen et al., 2023; Sheng et al., 2023; Fisher et al., 2024). Machine learning and deep learning have demonstrated their potential in assisting the diagnosis of Parkinson's disease, capable of characterizing disease stages and patient functional impairments to better understand the brain mechanisms of PD (Abós et al., 2017; Sivaranjini and Sujatha, 2019; Guo et al., 2022).

Although the diagnosis of PD primarily relies on clinical symptoms and signs, certain imaging techniques can assist in ruling out other diseases with similar symptoms. For instance, conventional magnetic resonance imaging (MRI) can exclude other brain pathologies such as brain tumors or strokes that may cause similar symptoms. Techniques like functional MRI (fMRI) (Shi et al., 2022, 2023) and positron emission tomography (PET) can detect patterns of activity and metabolic changes in the brain, providing real-time information about the functioning of different brain regions (Rischka et al., 2021; Malén et al., 2022). This helps to understand the abnormal patterns of brain activity in PD patients, particularly during the execution of motor and cognitive tasks (Haq et al., 2020). By using specific radioactive tracers, PET scans can detect molecular and biochemical processes in the brain, such as the integrity of the dopamine transport system (Riou et al., 2021; Nordin et al., 2022). This aids in early diagnosis and the assessment of potential new therapies. Structural imaging analysis using MRI can reveal structural changes in the brains of PD patients, such as iron deposition and atrophy in the substantia nigra pars compacta. While these changes are non-specific (Bouilleret et al., 2008), they can serve as biomarkers for disease progression (Fabbri et al., 2017; Du et al., 2018; Biondetti et al., 2020; He et al., 2020).

Given the lack of specific clinical presentations in the early stages of PD, and because the structural changes induced by PD are almost imperceptible to the naked eye on magnetic resonance imaging (MRI), developing an objective and accurate diagnostic method has become a focal point of research. Prior research had centered on the basal ganglia and cerebral cortex, but exploration of the cerebellum in PD patients has recently gained researchers' attention (Ko et al., 2017; Solana-Lavalle and Rosas-Romero, 2021; Pang et al., 2022; Wang et al., 2023). The cerebellum, as a brain region associated with motor functions, may reveal microstructural alterations related to PD through changes in its texture features. Considering the cerebellum's role in coordination and fine motor control, investigating the texture features of gray and white matter greatly contributes to a profound understanding of PD's neurodegenerative process (Deuter et al., 2023; Iskusnykh et al., 2024; Saban et al., 2024). The purpose of this paper is not only to explore the potential of Parkinson's disease diagnosis using machine learning with cerebellar gray matter and white matter texture features, but also to find the most diagnostically valuable factors through model visualization.

## Methods

### Subjects

Participants in this study were recruited from 2 cohorts: the Parkinson's Progression Markers Initiative (PPMI; http://www.ppmi-info.org) (Marek et al., 2011) and First Affiliated Hospital of Dalian Medical University. The inclusion criteria for Parkinson's disease patients in the PPMI cohort are as follows: (1) Imaging studies have not revealed tumors, strokes, or other lesions; (2) The image quality is good without artifacts; (3) The scanning range covers the whole brain. The detailed HC standards in the PPMI queue are found at https://www.ppmi-info.org/study-design/research-documents-and-sops. Healthy subjects who are demographically matched and do not have current or active neurological diseases will also be included in the study. Finally, 204 patients were included in the PPMI cohort, including 102 patients with Parkinson's disease and 102 HCs. The PPMI cohort served as the training set for the experiment. The data used in this study came from the PPMI website in November 2023. The cohort data obtained from PPMI was used as the training set of this study.

A total of 170 patient cohorts were included in our hospital, including 79 PD patients and 91 normal controls. The inclusion and exclusion criteria of all subjects in the study were consistent with the screening of PPMI. This part of the data was used as the test set of the model. The study received ethical approval from the Ethics Committee (approval number: LCKY2014-29), and written informed consent was obtained from all participants.

### MRI acquisition

Image acquisition parameters for the standardized acquisition were obtained from MRI scanner protocols provided by the Parkinson's Progression Markers Initiative (PPMI, https://ida.loni.usc.edu/pages/access/studyData). The detailed MRI acquisition parameters of enrolled patients in our hospital cohort were presented in Table 1.

**Table 1:** All MRI acquisition parameters.

	**General electric company (*n* = 76)**	**Philips, ingenia CX (*n =* 62)**	**United imaging uMR Omega (*n =* 32)**
Field/T	3.0	3.0	3.0
Coil/Channel	12	24	32
TR/ms	10.2	8.4	9
TE/ms	4.2	3.8	3.6
FOV	512 × 512	200 × 200	256 × 180
Thickness/mm	1	1	1
Slice Gap/mm	0	0	0
Pixel spacing	1 × 1 × 1	0.67 × 0.67	0.5 × 0.5

### Image preprocessing and radiomic feature extraction

**Figure 1:**
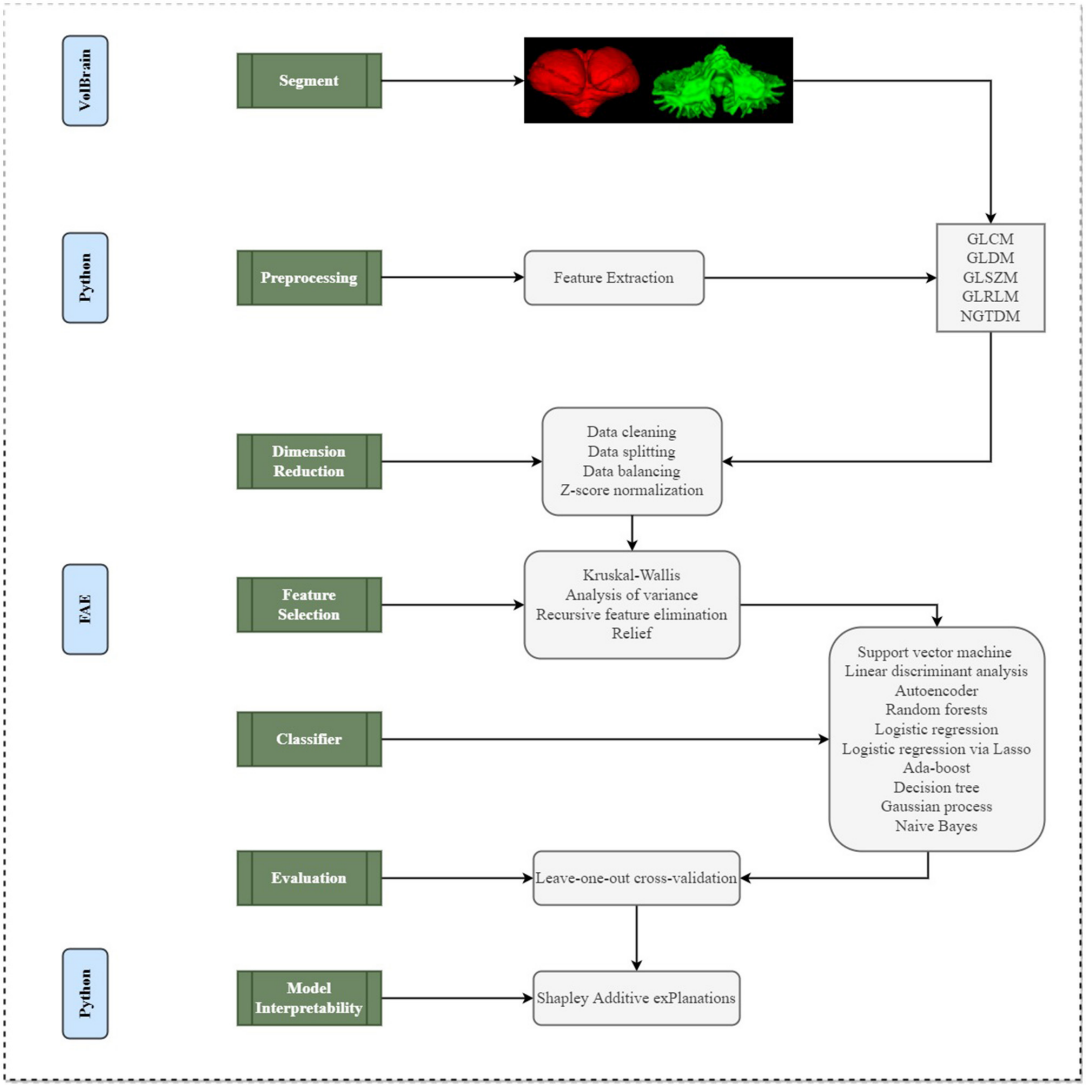
A schematic diagram for the whole radiomics and machine learning pipeline.

The detailed processes of image preprocessing and radiomic feature extraction were described in Figure 1. VolBrain (https://volbrain.upv.es/), which was a robust, high-precision automatic channel for brain segmentation is used to automatically extract masks from each cerebellum (Næss-Schmidt et al., 2016; Park et al., 2023). Prior to initiating the segmentation process, the images undergo a preprocessing routine to ensure uniformity in data analysis across different patients. This includes the application of the N4 bias correction algorithm to eliminate undesired low-frequency intensity variations, followed by standardization of the signal intensity through z-score normalization. Furthermore, all images were registered to the Montreal Neurological Institute space and resampled to achieve a consistent voxel resolution of 1 × 1 × 1 mm^3^. Subsequently, VolBrain employs a specialized cerebellar template for the individual segmentation of each image.

After the completion of image preprocessing, PyRadiomics was used to extract radiomics features of cerebellar gray and white matter, which conformed to the Image Biomarker Standardization Initiative. A total of 73 features were extracted each time, including 22 Gray-level co-occurrence matrix (GLCM) features, 14 Gray-level dependence matrix (GLDM) features, 16 Gray-level size zone matrix (GLSZM) features, 16 Gray-level size zone matrix (GLRLM) features, 5 Neighboring gray tone difference matrix (NGTDM) features. Details of these 73 features were described in Supplementary material. In this study, a total of 146 imaging features [73 features × 2 (cerebellar gray matter + white matter)] were extracted.

### Feature selections

We applied the normalization on the feature matrix. For each feature vector, we calculated the mean value and the standard deviation. Each feature vector was subtracted by the mean value and was divided by the standard deviation. After normalization process, each vector had zero center and unit standard deviation. Since the dimension of feature space was high, we compared the similarity of each feature pair. If the Pearson Correlation Coefficient (PCC) value of the feature pair was larger than 0.90, we removed one of them. After this process, the dimension of the feature space was reduced and each feature was independent to each other. Lastly, the analysis of variance (ANOVA), relief, Kruskal-Wallis (KW), and recursive feature elimination (RFE) were utilized for feature selections. ANOVA is a common analytic method to explore the significant features corresponding to the labels. The relief selected the sub-data set and finds the relative features according to label recursivity. KW test was a rank sum test, which sorted all data from small to large and calculates the rank of each data. The goal of RFE was to select features based on a classifier by recursively considering a smaller set of features, and the weight of each feature remained consistent. The feature number range was set from 1 to 20.

### Classification

The classification performances were tested with 10 ML algorithms based on Python code with scikit-learn library (https://scikit-learn.org/), including the support vector machine (SVM), linear discriminant analysis (LDA), autoencoder (AE), random forests (RF), logistic regression (LR), logistic regression via Lasso (LRLasso), ada-boost (AB), decision tree (DT), Gaussian process (GP), and naive Bayes (NB). These 10 machine learning algorithms will all be used to build models, and the indicator results of all models are provided in the Supplementary material.

All above processes were implemented with FeAture Explorer Pro (FAE, V 0.5.0) on Python (3.7.6) (Abraham et al., 2014; Song et al., 2020).

### Evaluations

This study utilized the 5-fold cross-validation method to assess the results. Additionally, the performance metrics such as accuracy, sensitivity, specificity, positive predictive value (PPV), and negative predictive value (NPV) were computed at a cutoff value that maximized the Youden index for each predictive model. The area under the receiver operating characteristic curve (AUC-ROC), a common measure of model performance, was then calculated for each tested condition (as depicted in Figure 1).

### Model interpretability

SHAP (Shapley Additive exPlanations) was a Python library used to interpret the prediction results of complex machine learning models (Kui et al., 2022; Yasin et al., 2023) (as depicted in Figure 1). Based on the Shapley value in game theory, it distributes the contribution of each characteristic (input variable) to the predicted output in a fair way. The Shapley value was a fair distribution solution, which takes into account the order of all possible feature contributions, and the results satisfy the principles of efficiency, symmetry, additivity, and zero contribution. The role of SHAP was mainly reflected in that it provides a transparent, intuitive, fair and consistent way to quantify and understand the impact of characteristics on model prediction. Through SHAP, we can get the impact of each feature on individual predictions and overall predictions, as well as compare features, so as to understand which features were more important to model prediction. The machine learning model we use usually determines the prediction results by a variety of input characteristics. To better understand this model and make it transparent and interpretable, we can use the SHAP library to see the extent to which each feature affects and how these features interact to affect predictions. The positive or negative SHAP value represents whether the influence of this characteristic on model prediction was promoted or suppressed. The larger the absolute value of SHAP value was, the greater the influence of this characteristic on model prediction was. Therefore, through the SHAP library, we could deeply interpret the prediction behavior of the model and visualize the influence of the characteristics, and optimize and improve it more effectively.

## Results

The baseline clinical characteristics of the 374 patients with subjects in the training set (*n* = 204) and test set (*n* = 170) were summarized in Table 2. In total, 102 (50.0%) and 79 (46.5%) subjects in the training group and test group were Parkinson's disease patients. None of the parameters tested revealed significant difference between groups.

**Table 2:** Clinical characteristics of all participants.

**Clinical variables**	**Training set (*****n =*** **204)**			**Test set (*****n =*** **170)**		
	**NC**	**PD**	***P*** **value**	**NC**	**PD**	***P*** **value**
	***N** =* **102**	***N** =* **102**		***N** =* **91**	***N** =* **79**	
Age (years)^a^	66.00 (62.00, 71.00)	66.00 (59.25, 70.00)	0.236	66.00 (62.00, 71.00)	67.00 (61.00, 71.00)	0.902
Gender, *n* (%)^b^			0.069	66.00 (62.00, 71.00)	67.00 (61.00, 71.00)	0.293
Male	43 (42.16)	56 (54.90)		32 (35.16)	34 (43.04)	
Female	59 (57.84)	46 (45.10)		59(64.84)	45(56.96)	
MDS-UPDRS III	NA	24.00 (15.00, 30.75)		NA	NA	
HY	NA	NA		NA	2.50 (2.00, 3.00)	

Data are presented as the mean ± SD (range) for normally distributed data or median (interquartile range) (range) for non-normally distributed data. ^a^The *P*-value was calculated using *t-*test. ^b^The *P*-value was calculated using the chi-square test. MDS-UPDRS, MDS Unified-Parkinson Disease Rating Scale; HY, Hoehn and Yahr staging scale.

**Figure 2:**
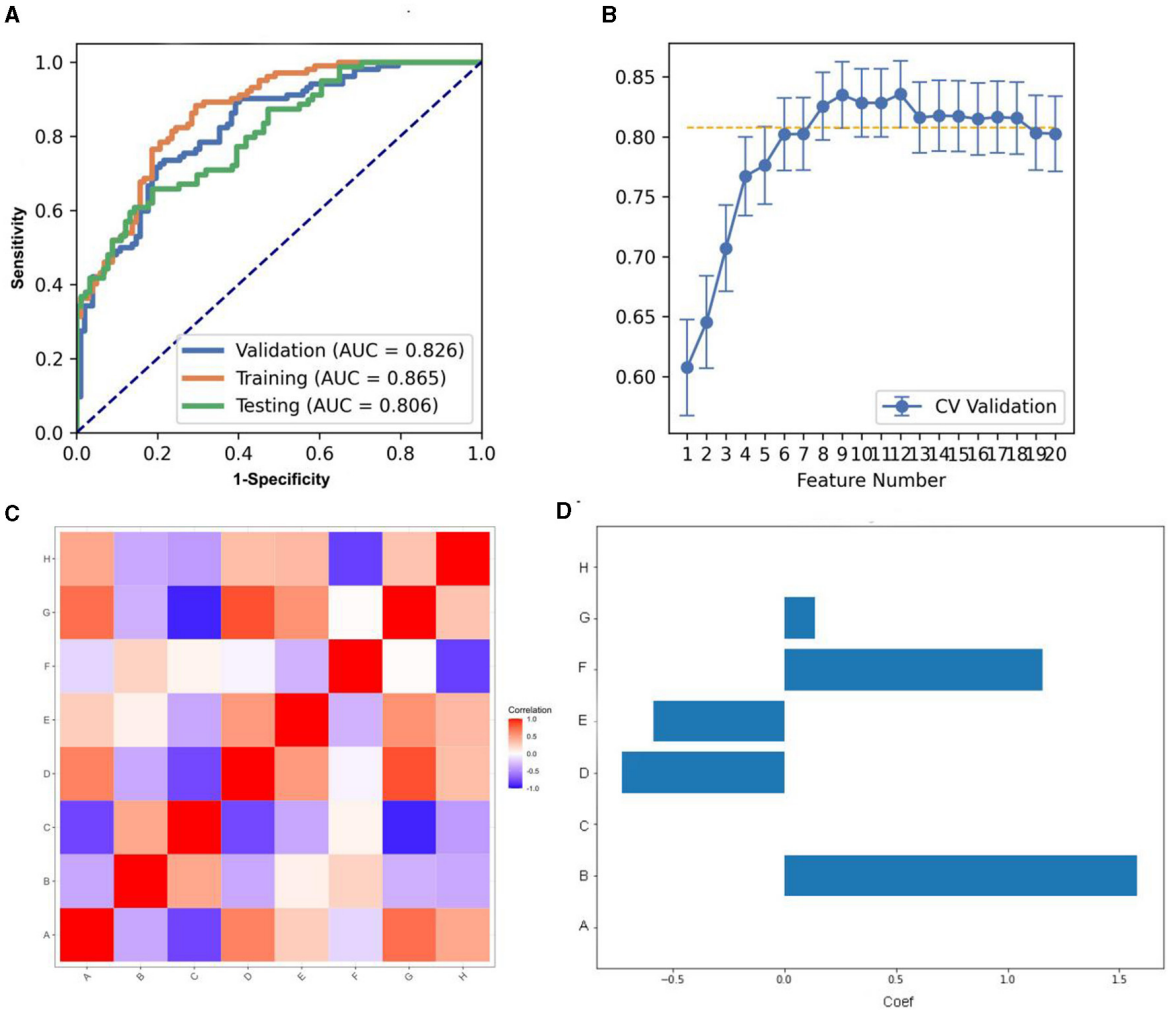
Model performance generated using Kruskal-Wallis (KW). **(A)** Receiver operating characteristic (ROC) curves of this model using different datasets. **(B)** FeAture Explorer (FAE) software suggested a candidate eight-feature model according to the “one-standard error” rule. **(C)** Heatmap of the correlation of the features in the final model. **(D)** Weight distribution map of features in the model.

We compared the AUC of all the pipelines on the validation dataset with FAE. The pipeline using KW feature selection and a SVM classifier yielded the highest AUCs using 12 features. When the “one-standard error” rule was used, FAE also produces an efficient and simple model. At this time, the pipeline used was the KW feature selection and LRLasso classifier, and 8 features were used for modeling. The ROC curves were shown in Figure 2A. The feature selection of this model was shown in Figure 2B. The AUC and the accuracy could achieve 0.826 and 0.760, respectively. In this point, The AUC and the accuracy of the model achieve 0.806 and 0.741 on testing data set. FAE-selected features were shown in Figure 2C, including one ngtdm feature, two gldm features, two glrlm features, and three glcm features. The selected features were shown in Table 3. The correlation heat map of the features in the final model was shown in Figure 2D.

**Table 3:** The coefficients of eight features of the model combined with REF and RF.

	**Feature**	**Coef in model**
A	WM_original_glcm_Idn	0.000
B	WM_original_glcm_Imc1	1.581
C	WM_original_glcm_InverseVariance	0.000
D	WM_original_gldm_DependenceNonUniformityNormalized	−0.726
E	WM_original_gldm_DependenceVariance	−0.585
F	WM_original_glrlm_GrayLevelNonUniformity	1.156
G	WM_original_glrlm_LongRunEmphasis	0.136
H	WM_original_ngtdm_Coarseness	0.000

**Figure 3:**
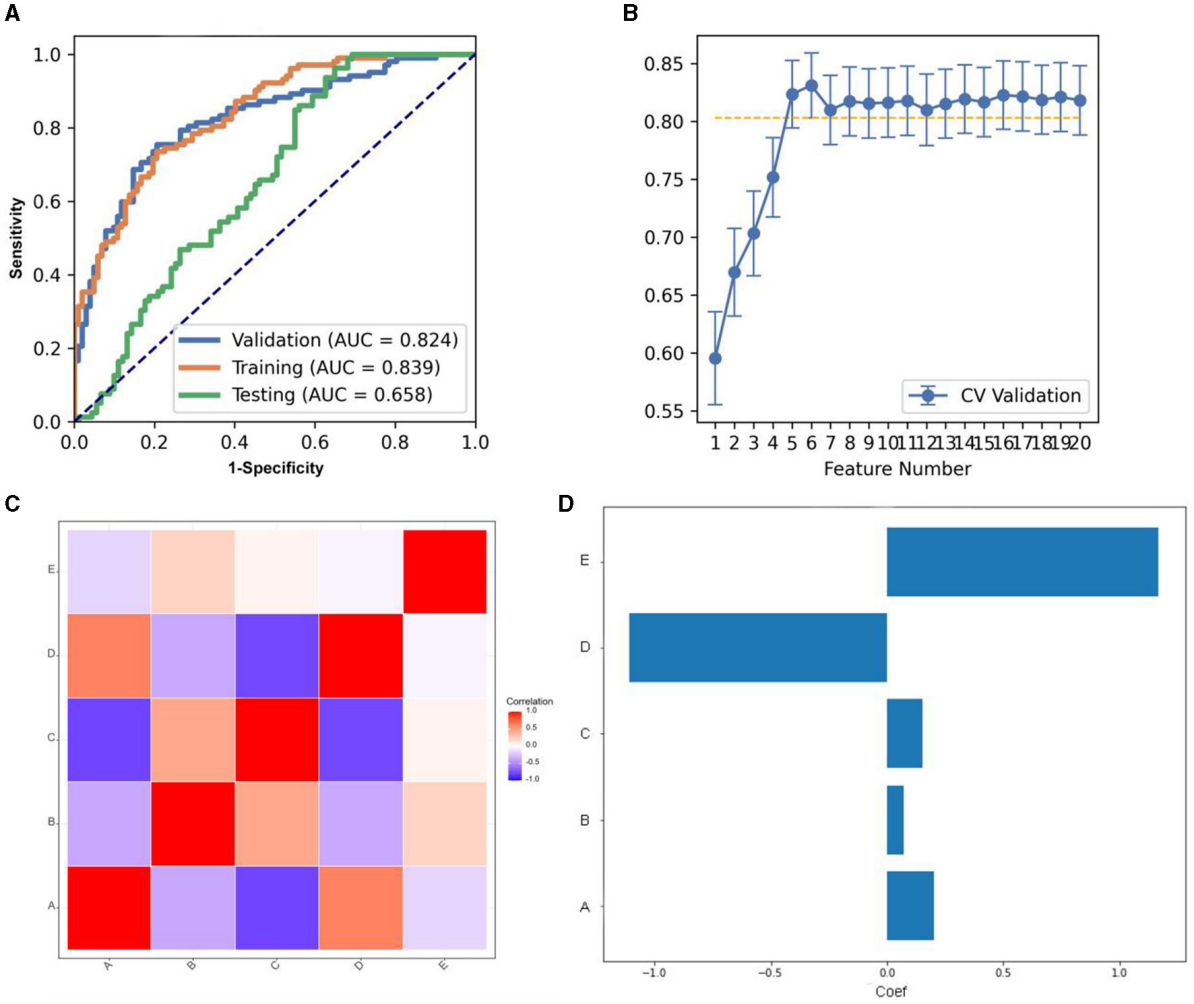
Model performance generated using analysis of variance (ANOVA). **(A)** Receiver operating characteristic (ROC) curves of this model using different datasets. **(B)** FeAture Explorer (FAE) software suggested a candidate five-feature model according to the “one-standard error” rule. **(C)** Heatmap of the correlation of the features in the final model. **(D)** Weight distribution map of features in the model.

As for ANOVA, the pipeline using the LDA classifier yielded the highest AUC using five features with a “one-standard error” rule. The AUC and the accuracy could achieve 0.824 and 0.774, respectively. In this point, the AUC and the accuracy of the model achieve 0.0.658 and 0.635 on testing data set (Figure 3A). The features selected via FAE were depicted in Figures 3B, C, which include one gldm feature, one gldm feature, and three glcm features. These selected attributes were detailed in Supplementary Table 5. Figure 3D presents a heat map that illustrates the correlations among the features incorporated into the final model.

**Figure 4:**
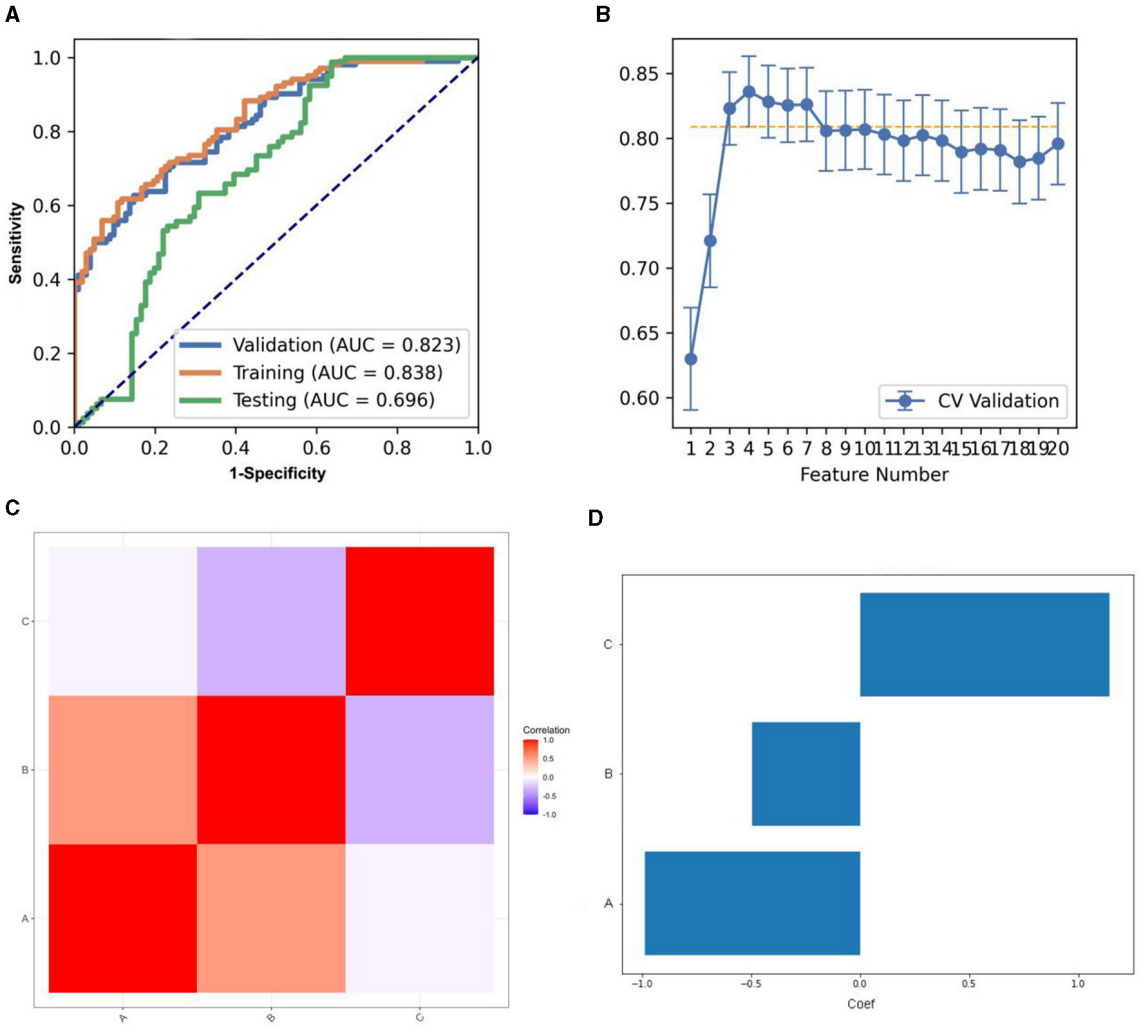
Model performance generated using Recursive feature elimination (REF). **(A)** Receiver operating characteristic (ROC) curves of this model using different datasets. **(B)** FeAture Explorer (FAE) software suggested a candidate seven-feature model according to the “one-standard error” rule. **(C)** Heatmap of the correlation of the features in the final model. **(D)** Weight distribution map of features in the model.

Concerning the REF, the pipeline deploying an LDA classifier secured the highest AUC while utilizing three features, in accordance with the “one-standard error” rule. Concurrently, FAE furnished a three-feature model, as depicted in Figure 4. The model's AUC and accuracy reached 0.823 and 0.740, respectively. At this juncture, on the testing dataset, the model's AUC and accuracy stood at 0.696 and 0.653, respectively. The FAE selected three features, illustrated in Figure 4C, which comprise one glrlm feature and two gldm features. Supplementary Table 6 delineated the designated features for the model along with their corresponding coefficients.

**Figure 5:**
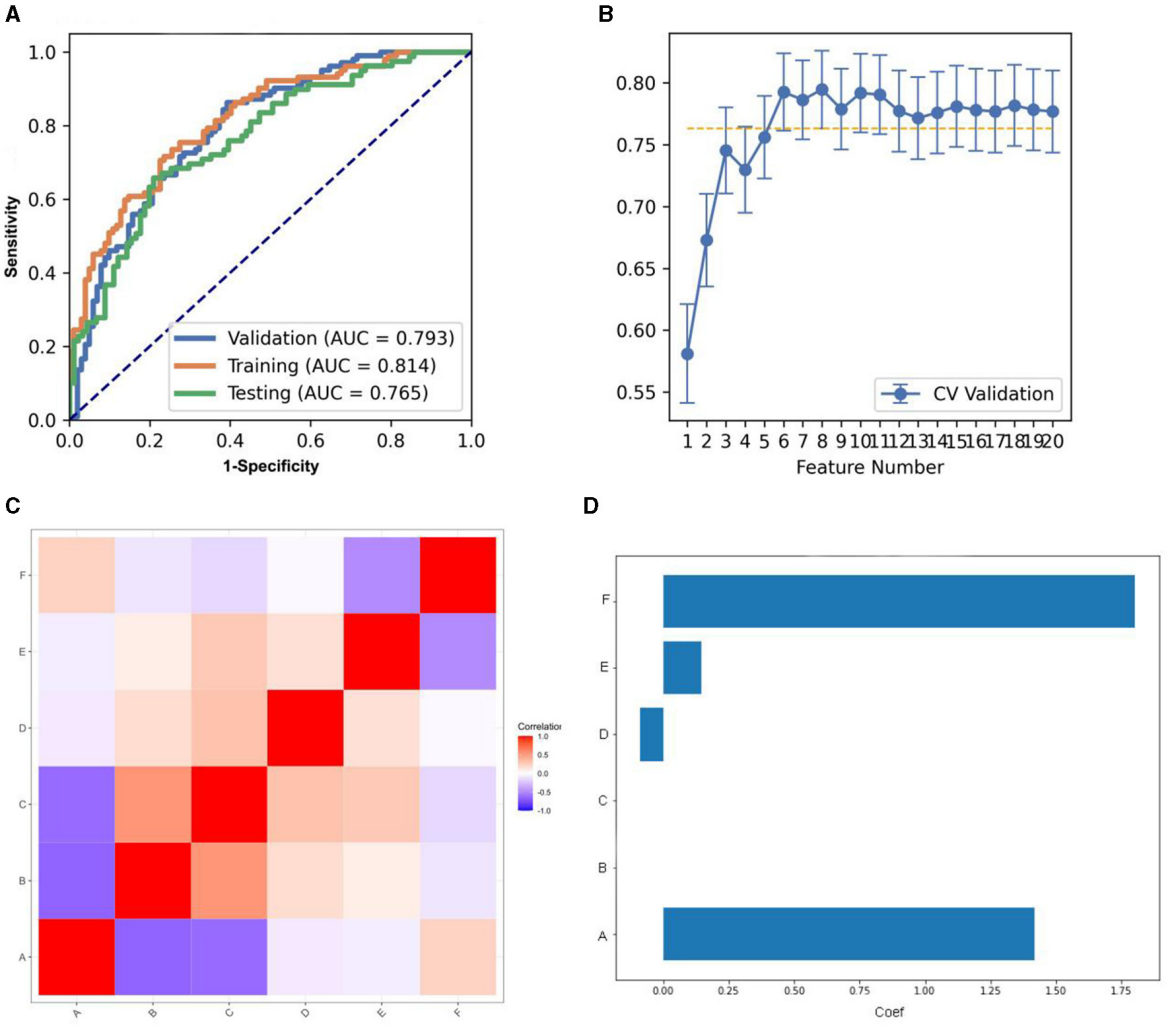
Model performance generated using Relief. **(A)** Receiver operating characteristic (ROC) curves of this model using different datasets. **(B)** FeAture Explorer (FAE) software suggested a candidate six-feature model according to the “one-standard error” rule. **(C)** Heatmap of the correlation of the features in the final model. **(D)** Weight distribution map of features in the model.

Regarding Relief, the LRLasso classifier-based pipeline, while adhering to the “one-standard error” rule, had yielded the most robust AUC. Concurrently, the implementation of the FAE had resulted in a model encapsulating six features, as depicted in Figure 5. The performance metrics, AUC and accuracy, had impressively reached 0.793 and 0.735, respectively. Pertinently, when assessed on the testing dataset, the model's AUC and accuracy stood at 0.764 and 0.729. The six features curated by the FAE, presented in Figure 5C, encompass a diverse set including three glcm features, one gldm feature, one glrlm feature, and one ngtdm feature. Among these, two were sourced from the cerebellar gray matter and four from cerebellar white matter. The nomenclature and corresponding coefficients of the features adopted for the model were systematically cataloged in Supplementary Table 7.

### Comparison of model performances

**Figure 6:**
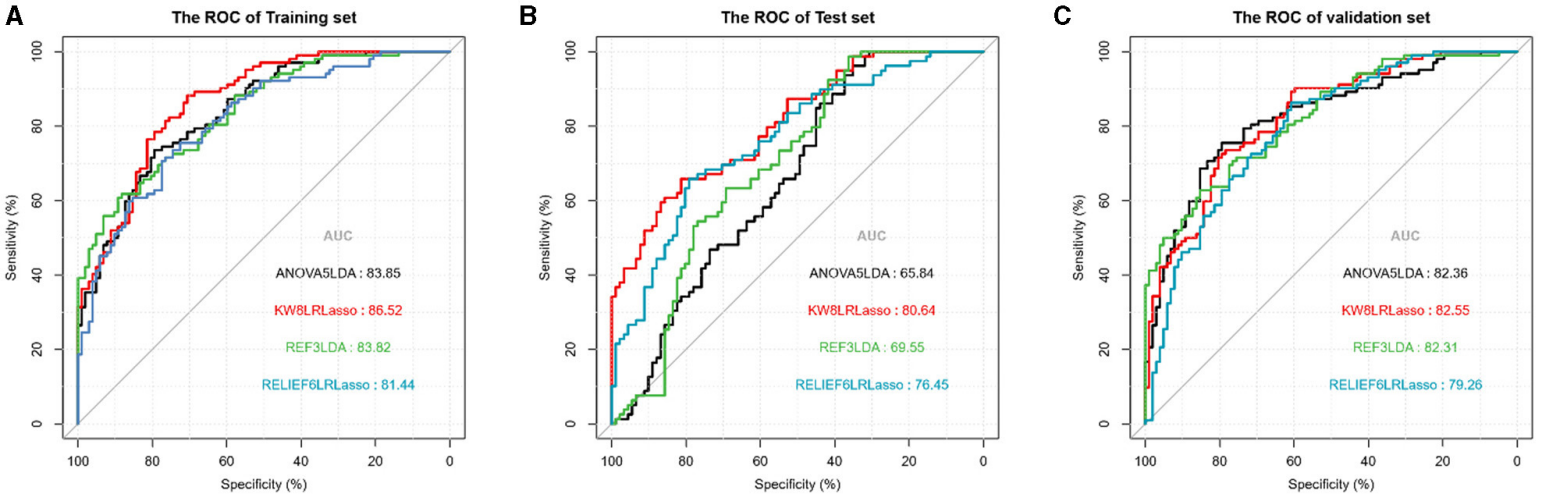
Receiver operating characteristics curves of the models in the training, test, and validation sets.

In the training set, the KW8LRLasso model generally demonstrated enhanced performance relative to the other models evaluated. Although there are differences in AUC among the four models, it was found that not all of these differences are statistically significant. The data from this study revealed a statistical difference in AUC between the KW8LRLasso model and the ANOVA5LDA model (AUC: 0.865 vs. 0.839, *P* = 0.0343), as well as a statistical difference in AUC between the KW8LRLasso model and the RELIEF6LRLasso model (*P* = 0.0112). In the test set, the performance of the KW8LRLasso model was superior to other models. There was a significant difference in AUC between the ANOVA5LDA model and the KW8LRLasso model (AUC: 0.651 vs. 0.768, *P* < 0.001). Additionally, there was also a significant difference in AUC between the KW8LRLasso model and the REF3LDA model (AUC: 0.768 vs. 0.685, *P* < 0.001). In the validation set, the KW8LRLasso model tended to show superior performance compared to others. However, there was no statistical difference in effectiveness between the four models. The detailed comparison of each model can be found in Table 4, and their ROC curves were shown in Figure 6.

**Table 4:** The performances of four models in the training, test, and validation sets.

	**ANOVA5LDA**	**KW8LRLasso**	**REF3LDA**	**RELIEF6LRLasso**	**P**
Training set	0.839	0.865	0.838	0.814	0.034^a^
Test set	0.651	0.768	0.685	0.733	*P* < 0.001^b, e^
					0.003^c^
					0.004^d^
Validation set	0.824	0.826	0.823	0.793	

^a^ANOVA5LDA ~ KW8LRLasso, ^b^ANOVA5LDA ~ KW8LRLasso, ^c^ANOVA5LDA ~ REF3LDA, ^d^ANOVA5LDA ~ RELIEF6LRLasso, ^e^KW8LRLasso ~ REF3LDA.

In addition to the feature selection methods, we also compared and listed the optimal AUCs of different ML classifications in the validation dataset (Supplementary Table 8).

### Model interpretability with SHAP

**Figure 7:**
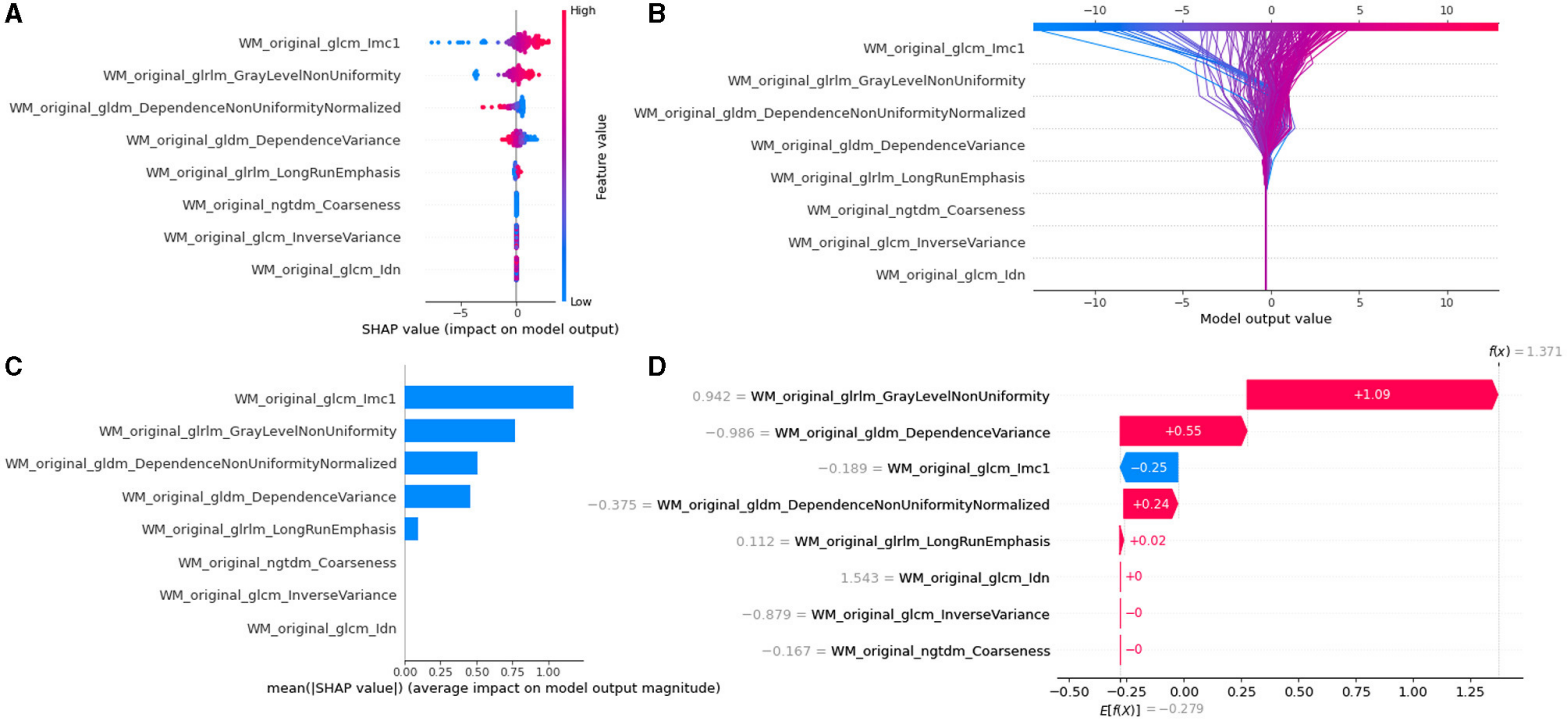
Model interpretability of the KW8LRLasso model with SHapley Additive exPlanations (SHAP). **(A)** Summary plot of feature impact on the decision of the model showing positive and negative relationships of the predictors with the target variable. A positive SHAP value indicates an increase in the probability of PD. **(B)** Decision diagram of the KW8LRLasso model. The vertical gray line in the middle of the decision graph represents the basic value of the model. The colored line, on the other hand, indicates the prediction and shows whether each feature moves the output value above or below the average predicted value. The eigenvalues are positioned next to the prediction line for reference. Moving to the bottom of the diagram, the prediction line demonstrates the accumulation of SHAP value from the base value to the final score of the model at the top of the diagram. **(C)** Variance importance plot listing the most significant variables. The features that were more valuable for the diagnosis of PD were located at the top, presented in descending order. **(D)** Waterfall diagram of the first sample in the KW8LRLasso model. The waterfall diagram is designed to provide an explanation for a single prediction. It takes a single line of the interpreted object as input. The diagram begins with the expected value of the model output at the bottom. Each row then indicates whether each feature has a positive (red) or negative (blue) contribution. In other words, it shows how the value is pushed from the model's expected output value on the data set to the model's predicted output value. It is worth noting that the most contributing factor is 'WM_original_glrlm_GrayLevelNonUniformity'.

The SHAP values for each selected feature in the KW8LRLasso model were calculated, and the relevant plots were shown in Figure 7. Shapley values were used to measure the impact and sign of each radiomic feature in model prediction. Features with positive SHAP values contribute to the prediction of subjects with PD, while features with negative SHAP values aid in predicting subjects without PD. Details of other models can be found in Supplementary Figures 8–10.

## Discussion

In this study, our results support our basic hypothesis that the texture features of the cerebellum can provide a robust distinguishing feature space between PD and HC. Through the visualization of the model by SHAP, we found that the texture features extracted from the white matter of the cerebellum have greater diagnostic value.

The ROIs (cerebellar gray matter and white matter) selected in the present study included regions that are known to be involved in the pathogenesis of PD. Actually, pathophysiological and atrophic changes in the cerebellum are documented in Parkinson's disease (O'Callaghan et al., 2016; Ko et al., 2017). Usually, abnormal changes in the cerebellum in patients with Parkinson's disease involve not only structural changes, but also functional changes. Studies by O'Callaghan et al. have shown that although cerebellum atrophy occurs in patients with Parkinson's disease, this change is miraculously accompanied by an increase in the connection between the two functional subsystems (O'Callaghan et al., 2016). More importantly, the evidence showed that the abnormality of cortical network in patients with Parkinson's disease is related to the degree of local cerebellar atrophy, which indicated that the internal structural changes of cerebellum play a greater role in Parkinson's disease (O'Callaghan et al., 2016). Increased intracerebellar connectivity may reflect pathological, as opposed to compensatory, activity (Pedroso et al., 2013; O'Callaghan et al., 2016). Riou et al. (2021) assessed the motor, cognitive and mental status of Parkinson's patients, combined with resting 18FDG-PET metabolic imaging to explore the relationship between these three aspects and cerebellar metabolism. The results show that the cerebellum not only plays a role in motor symptoms, but also can be extended to other non-motor symptoms (such as cognitive, mental, etc.).

Quantitative imaging, such as IBSI (Zwanenburg et al., 2020) (Image Biomarker Standardization Program), based radiomics had gained increasing attention in the field of oncology, while methods for identifying PD and HC were limited. Image textures generally define the finer spatial organization within an image or region of interest (Park and Kim, 2018; van Timmeren et al., 2020; Le et al., 2021). GLCM describes the frequency of occurrence of adjacent pixel pairs with specific gray levels within an image. For each pair of neighboring pixels, the GLCM calculates the probability of their gray levels occurring together. GLDM was a method for quantifying gray level dependencies within an image. It operates by measuring the differences in gray level values between a pixel and its surrounding pixels within a local area. Unlike the GLCM, the GLDM focuses on the relationship of a pixel to its surrounding pixels, rather than just to its immediate neighbors. GLSZM represents the size of contiguous pixel areas, also known as “size zones,” that share the same gray level value within the image. By doing so, the GLSZM quantifies the size of common areas within the image, which was directly related to the distribution of gray levels and helps to describe the coarseness of textures within the image. GLRLM measures the length of consecutive occurrences of the same gray level value within an image (Palani et al., 2023). A run length refers to a sequence of consecutive pixels in a specific direction that had the same gray level. NGTDM was a method for characterizing image texture, taking into account the differences in gray level values between a pixel and the pixels in its neighborhood (Liang et al., 2021).

Increasing evidence suggests that the cerebellum plays a role in the pathophysiology of Parkinson's disease (Vercruysse et al., 2015; Zeng et al., 2016; Rusholt et al., 2020; Zwanenburg et al., 2020; Yan et al., 2023; Zhong et al., 2023). However, most of the past radiomics studies on Parkinson's disease had focused on the basal ganglia (Park et al., 2023; Sun et al., 2023), and the value of advanced texture features in the gray and white matter of the cerebellum had been greatly underestimated. In this study, the common parameters used to differentiate PD from HC was WM_original_glrlm_GrayLevelNonUniformity in the four feature selection methods. Therefore, we consider this feature as the most stable and efficient feature, contributing to each model. The “Original_glrlm_GrayLevelNonUniformity” measures the similarity of gray-level intensity values in an image, such that a higher value correlates with lesser similarity and greater heterogeneity. In the cases included in this study, the Original_glrlm_GrayLevelNonUniformity was higher in the PD group than in the HC group, reflecting the variation and distribution of grayscale values within T1WI images of white matter in the cerebellum of PD patients, which were more complex and varied compared to the HC group. Parkinson's disease is a common neurodegenerative disease characterized by the abnormal accumulation of α-synuclein in the cytoplasm of oligodendrocytes, known as glial cytoplasmic inclusions (GCI) (Dickson, 2012; Kaindlstorfer et al., 2018). Abnormal accumulation of alpha-synuclein in OLG cells can lead to pathological changes such as neuronal degeneration, neuroinflammatory responses, and oxidative stress, which may accelerate the progression of neurodegenerative diseases. This abnormal accumulation is likely one of the key factors leading to the death of substantia nigra dopamine neurons. In Parkinson's disease (PD), the death of these neurons can result in damage to the pathways between the substantia nigra and the striatum. These pathways include numerous white matter tracts, such as the cortico-basal ganglia-brainstem pathway, the cortico-spinal tract, and the cortico-thalamic tract, among others. Therefore, these white matter tracts are also affected. Based on this pathological change, PD patients often exhibit signs of demyelination, axonal loss, and gliosis in their white matter, resulting in significant atrophy of the cerebellar white matter in PD patients. Rusholt et al. (2020) also confirmed this view. They found that cerebellar white matter atrophy in patients with PD was significantly higher than that in healthy controls, but there was no significant difference in cerebellar cortical volume between the two groups. Previous studies have confirmed the potential importance of white matter changes in the pathology of PD, particularly in the cerebellum (Rusholt et al., 2020; Yan et al., 2023; Zhong et al., 2023), where damaged white matter changes are a prominent characteristic of PD (Rusholt et al., 2020). Previous studies have shown that gray matter atrophy in PD patients is particularly prominent in the cerebral cortex, while there are only a few small brain regions in the cerebellum with abnormal atrophy (Nishio et al., 2010; Barber et al., 2017; Xuan et al., 2017). Xuan et al. (2017) have suggested that this may be due to compensatory changes in the gray matter of the cerebellum. Rusholt et al. (2020)'s findings suggested that there was no significant volume change in the entire gray matter of the cerebellum or its main branches, which again emphasized the importance of white matter changes in exploring PD pathological changes. In this study, quantitative texture features of the white matter were found to be more valuable than those of the gray matter, consistent with the pathological basis and previous studies. Therefore, leveraging the heterogeneity of white matter changes, we constructed an efficient diagnostic model based on advanced machine learning algorithms and visualized the contribution values of quantitative texture feature imaging biomarkers using SHAP.

ANOVA, Relief, KW, and RFE were four feature selection methods, each with its own set of advantages and disadvantages, suitable for different circumstances. ANOVA was computationally simple, easy to understand, and implement, yet it may overlook significant feature combinations and struggle with non-linear relationships. Relief took into account the interdependencies between features; however, its computational cost can become substantial when dealing with a large number of features. KW was capable of handling non-linear relationships and interactions but is sensitive to outliers and missing values. RFE enhanced model interpretability by recursively selecting features, but it may lead to overfitting, particularly when there were few features or the dataset was small.

After comparing the indicators of the four models across three data sets, it could be concluded that the KW8LRLasso model exhibits the highest level of performance in this study. Through the implementation of the KW feature screening method, effective feature selection is achieved. This method not only enhances the performance and efficiency of the model but also improves its interpretability and generalizability, particularly when confronted with high-dimensional data. The LRLasso algorithm is known for its strong performance in high-dimensional data analysis and feature selection. It can effectively handle datasets with a large number of variables and improve both the stability and interpretability of the model. Additionally, the regularization feature of the algorithm helps mitigate multicollinearity issues and enhances the model's robustness. These advantages may explain why the KW8LRLasso model is superior to the other three models. However, when applying these findings clinically, it is important to consider more complex factors. It is necessary to increase the sample size in order to enhance the model's universality, stability, and sensitivity. This will ensure that the study's conclusions are not solely based on the current data set.

Our study has some limitations. First, this study only included radiomics features and did not involve clinical-related data, lacking a systematic and comprehensive investigation. In future research, it is necessary to incorporate as detailed clinical information as possible. Secondly, this study did not classify Parkinson's patients into phenotypes; the next step would be to expand the sample size and subdivide into subtypes. Lastly, we treated the cerebellum as an individual entity and only constructed models based on cerebellar structural images; subsequent studies could incorporate more functional images and sequences. In future research, acquiring high-quality functional imaging data and subsequently integrating functional connectivity analysis within the domain of machine learning presents an intriguing possibility.

## Conclusion

This study focused on the cerebellum and extracts texture features from 3DT1WI images of its gray and white matter to establish a model for diagnosing Parkinson's disease. Through the visualization of the model, the heterogeneity of the cerebellar white matter in PD patients was highlighted. Future research can focus on delving into these complex texture features and exploring their relationship with potential pathological microstructural changes.

## Data availability statement

The original contributions presented in the study are included in the article/Supplementary material, further inquiries can be directed to the corresponding authors.

## Ethics statement

The studies involving humans were approved by the Ethics Committee of Dalian Medical University. The studies were conducted in accordance with the local legislation and institutional requirements. The participants provided their written informed consent to participate in this study.

## Author contributions

YC: Formal analysis, Investigation, Methodology, Software, Validation, Visualization, Writing – original draft, Writing – review & editing, Resources. YQ: Investigation, Software, Validation, Writing – original draft. TL: Software, Validation, Writing – original draft, Funding acquisition, Supervision, Visualization, Writing – review & editing, Project administration, Resources. AL: Visualization, Writing – original draft, Data curation, Investigation, Methodology, Resources. YN: Data curation, Investigation, Methodology, Visualization, Writing – original draft, Software, Formal analysis, Validation. RP: Methodology, Writing – original draft, Project administration, Resources, Supervision, Validation, Writing – review & editing. BS: Methodology, Supervision, Validation, Writing – review & editing, Conceptualization, Visualization.
