# Corrigendum: A more objective PD diagnostic model: integrating texture feature markers of cerebellar gray matter and white matter through machine learning

**DOI:** 10.3389/fnagi.2024.1490807

**Published:** 2024-10-03

**Authors:** Yini Chen, Yiwei Qi, Tianbai Li, Andong Lin, Yang Ni, Renwang Pu, Bo Sun

**Affiliations:** ^1^Department of Radiology, The First Affiliated Hospital of Dalian Medical University, Dalian, China; ^2^Liaoning Provincial Key Laboratory for Research on the Pathogenic Mechanisms of Neurological Diseases, The First Affiliated Hospital, Dalian Medical University, Dalian, China; ^3^Department of Neurology, Zhejiang Taizhou Municipal Hospital, Taizhou, Zhejiang, China

**Keywords:** Parkinson's disease, radiomic, machine learning, SHAP, FeAture Explorer

In the published article, there was an error. The model was incorrectly described as being composed of a single feature. A correction has been made to **Results**, paragraph four. This sentence previously stated:

“Concerning the REF, the pipeline deploying an LDA classifier secured the highest AUC while utilizing merely a single feature, in accordance with the “one-standard error” rule.”

The corrected sentence appears below:

“Concerning the REF, the pipeline deploying an LDA classifier secured the highest AUC while utilizing three features, in accordance with the “one-standard error” rule.”

In the published article, there was an error. The model was incorrectly described as being composed of a single feature. A correction has been made to **Results**, paragraph five. A sentence has been removed as it was redundant. This sentence previously stated:

“This was accomplished by utilizing a mere single feature.”

In the published article, there was an error. The model was incorrectly described as being composed of seventeen features. A correction has been made to **Results**, paragraph five. This sentence previously stated:

“The seventeen features curated by the FAE, presented in Figure 5C, encompass a diverse set including three glcm features, one gldm feature, one glrlm feature, and one ngtdm feature.”

The corrected sentence appears below:

“The six features curated by the FAE, presented in Figure 5C, encompass a diverse set including three glcm features, one gldm feature, one glrlm feature, and one ngtdm feature.”

In the published article, there was an error. A correction has been made to **Discussion**, paragraph one. A sentence has been removed as it was redundant. This sentence previously stated:

“This study established a robust model, and more importantly, through the visualization of the model by SHAP, it was found that the texture features extracted from the white matter of the cerebellum have greater diagnostic value.”

The authors apologize for these errors and state that this does not change the scientific conclusions of the article in any way. The original article has been updated.

