# Supplementary material for: A more objective PD diagnostic model: integrating texture feature markers of cerebellar gray matter and white matter through machine learning
Source: Front Aging Neurosci. 2024 Jun 7;16:1393841. doi: 10.3389/fnagi.2024.1393841 (PMC11190310; doi:10.3389/fnagi.2024.1393841)
Supplement: Supplementary file 1 [file Data_Sheet_1.docx]

## Supplementary Tables

Table 5. The coefficients of features in the model of ANOVA and LDA.

|  | Feature | Coef in model |
| --- | --- | --- |
| A | WM_original_glcm_Idn | 0.203 |
| B | WM_original_glcm_Imc1 | 0.074 |
| C | WM_original_glcm_InverseVariance | 0.152 |
| D | WM_original_gldm_DependenceNonUniformityNormalized | -1.108 |
| E | WM_original_glrlm_GrayLevelNonUniformity | 1.168 |

Table 6. The coefficients of features in the model of REF and LDA.

|  | Feature | Coef in model |
| --- | --- | --- |
| A | WM_original_gldm_DependenceNonUniformityNormalized | -0.987 |
| B | WM_original_gldm_DependenceVariance | -0.493 |
| C | WM_original_glrlm_GrayLevelNonUniformity | 1.146 |

Table 7. The coefficients of features in the model of Relief and LRLasso.

|  | Feature | Coef in model |
| --- | --- | --- |
| A | WM_original_glrlm_GrayLevelNonUniformity | 1.419 |
| B | WM_original_ngtdm_Strength | 0.000 |
| C | WM_original_glcm_ClusterProminence | 0.000 |
| D | GM_original_gldm_DependenceNonUniformity | -0.087 |
| E | GM_original_glcm_Correlation | 0.147 |
| F | WM_original_glcm_Imc1 | 1.800 |

Table 8. The optimal area under the receiver operator characteristics curve (AUC), 95% confdence interval (CI), standard error (Std), accuracy, Youden index, sensitivity, specifcity, accuracy, positive predictive value (PPV), and negative predictive value (NPV) of all algorithm classifcations with leave-one-out cross-validation

|  | AUC | 95% CIs | Std | Cutoff | MCC | Acc | Youden Index | Sen | Spe | PPV | NPV |
| --- | --- | --- | --- | --- | --- | --- | --- | --- | --- | --- | --- |
| Zscore_PCC_KW_8_LRLasso | 0.8255 | [0.7701-0.8810] | 0.0283 | 0.8271 | 0.5755 | 0.5216 | 0.7598 | 1.0882 | 0.7157 | 0.8039 | 0.7849 |
| Zscore_PCC_ANOVA_5_LDA | 0.8236 | [0.7665-0.8808] | 0.0291 | 0.8336 | 0.4884 | 0.5494 | 0.7745 | 1.0392 | 0.7549 | 0.7941 | 0.7857 |
| Zscore_PCC_RFE_3_LDA | 0.8231 | 0.696 | Zscore_PCC_RFE_3_LDA | 0.8231 | 0.696 | Zscore_PCC_RFE_3_LDA | 0.8231 | 0.696 | Zscore_PCC_RFE_3_LDA | 0.8231 | 0.696 |
| Zscore_PCC_RFE_3_LR | 0.8223 | 0.7203 | Zscore_PCC_RFE_3_LR | 0.8223 | 0.7203 | Zscore_PCC_RFE_3_LR | 0.8223 | 0.7203 | Zscore_PCC_RFE_3_LR | 0.8223 | 0.7203 |
| Zscore_PCC_KW_8_LR | 0.822 | 0.75 | Zscore_PCC_KW_8_LR | 0.822 | 0.75 | Zscore_PCC_KW_8_LR | 0.822 | 0.75 | Zscore_PCC_KW_8_LR | 0.822 | 0.75 |
| Zscore_PCC_RFE_3_SVM | 0.8219 | 0.7217 | Zscore_PCC_RFE_3_SVM | 0.8219 | 0.7217 | Zscore_PCC_RFE_3_SVM | 0.8219 | 0.7217 | Zscore_PCC_RFE_3_SVM | 0.8219 | 0.7217 |
| Zscore_PCC_RFE_3_LRLasso | 0.8217 | 0.7143 | Zscore_PCC_RFE_3_LRLasso | 0.8217 | 0.7143 | Zscore_PCC_RFE_3_LRLasso | 0.8217 | 0.7143 | Zscore_PCC_RFE_3_LRLasso | 0.8217 | 0.7143 |
| Zscore_PCC_RFE_3_LRLasso | 0.8217 | 0.7143 | Zscore_PCC_RFE_3_LRLasso | 0.8217 | 0.7143 | Zscore_PCC_RFE_3_LRLasso | 0.8217 | 0.7143 | Zscore_PCC_RFE_3_LRLasso | 0.8217 | 0.7143 |
| Zscore_PCC_KW_8_SVM | 0.8198 | 0.7297 | Zscore_PCC_KW_8_SVM | 0.8198 | 0.7297 | Zscore_PCC_KW_8_SVM | 0.8198 | 0.7297 | Zscore_PCC_KW_8_SVM | 0.8198 | 0.7297 |
| Zscore_PCC_ANOVA_5_LRLasso | 0.8175 | 0.7549 | Zscore_PCC_ANOVA_5_LRLasso | 0.8175 | 0.7549 | Zscore_PCC_ANOVA_5_LRLasso | 0.8175 | 0.7549 | Zscore_PCC_ANOVA_5_LRLasso | 0.8175 | 0.7549 |
| Zscore_PCC_ANOVA_5_LRLasso | 0.8175 | 0.7549 | Zscore_PCC_ANOVA_5_LRLasso | 0.8175 | 0.7549 | Zscore_PCC_ANOVA_5_LRLasso | 0.8175 | 0.7549 | Zscore_PCC_ANOVA_5_LRLasso | 0.8175 | 0.7549 |
| Zscore_PCC_RFE_3_GP | 0.8104 | 0.7407 | Zscore_PCC_RFE_3_GP | 0.8104 | 0.7407 | Zscore_PCC_RFE_3_GP | 0.8104 | 0.7407 | Zscore_PCC_RFE_3_GP | 0.8104 | 0.7407 |
| Zscore_PCC_RFE_4_RF | 0.8095 | 0.7327 | Zscore_PCC_RFE_4_RF | 0.8095 | 0.7327 | Zscore_PCC_RFE_4_RF | 0.8095 | 0.7327 | Zscore_PCC_RFE_4_RF | 0.8095 | 0.7596 |
| Zscore_PCC_KW_8_LDA | 0.8051 | 0.8049 | Zscore_PCC_KW_8_LDA | 0.8051 | 0.8049 | Zscore_PCC_KW_8_LDA | 0.8051 | 0.8049 | Zscore_PCC_KW_8_LDA | 0.8051 | 0.8049 |
| Zscore_PCC_KW_8_RF | 0.7962 | 0.7647 | Zscore_PCC_KW_8_RF | 0.7962 | 0.7647 | Zscore_PCC_KW_8_RF | 0.7962 | 0.7647 | Zscore_PCC_KW_8_RF | 0.7962 | 0.7647 |
| Zscore_PCC_KW_6_GP | 0.7958 | 0.7105 | Zscore_PCC_KW_6_GP | 0.7958 | 0.7105 | Zscore_PCC_KW_6_GP | 0.7958 | 0.7105 | Zscore_PCC_KW_6_GP | 0.7958 | 0.7105 |
| Zscore_PCC_ANOVA_13_AB | 0.7947 | 0.7451 | Zscore_PCC_ANOVA_13_AB | 0.7947 | 0.7451 | Zscore_PCC_ANOVA_13_AB | 0.7947 | 0.7451 | Zscore_PCC_ANOVA_13_AB | 0.7947 | 0.7451 |
| Zscore_PCC_Relief_6_LRLasso | 0.7926 | 0.8158 | Zscore_PCC_Relief_6_LRLasso | 0.7926 | 0.8158 | Zscore_PCC_Relief_6_LRLasso | 0.7926 | 0.8158 | Zscore_PCC_Relief_6_LRLasso | 0.7926 | 0.8158 |
| Zscore_PCC_Relief_6_SVM | 0.7921 | 0.7882 | Zscore_PCC_Relief_6_SVM | 0.7921 | 0.7882 | Zscore_PCC_Relief_6_SVM | 0.7921 | 0.7882 | Zscore_PCC_Relief_6_SVM | 0.7921 | 0.7882 |
| Zscore_PCC_Relief_6_SVM | 0.7921 | 0.7882 | Zscore_PCC_Relief_6_SVM | 0.7921 | 0.7882 | Zscore_PCC_Relief_6_SVM | 0.7921 | 0.7882 | Zscore_PCC_Relief_6_SVM | 0.7921 | 0.7882 |
| Zscore_PCC_ANOVA_5_GP | 0.7905 | 0.7523 | Zscore_PCC_ANOVA_5_GP | 0.7905 | 0.7523 | Zscore_PCC_ANOVA_5_GP | 0.7905 | 0.7523 | Zscore_PCC_ANOVA_5_GP | 0.7905 | 0.7523 |
| Zscore_PCC_ANOVA_8_RF | 0.7901 | 0.8148 | Zscore_PCC_ANOVA_8_RF | 0.7901 | 0.8148 | Zscore_PCC_ANOVA_8_RF | 0.7901 | 0.8148 | Zscore_PCC_ANOVA_8_RF | 0.7901 | 0.8148 |
| Zscore_PCC_Relief_6_RF | 0.7901 | 0.7717 | Zscore_PCC_Relief_6_RF | 0.7901 | 0.7717 | Zscore_PCC_Relief_6_RF | 0.7901 | 0.7717 | Zscore_PCC_Relief_6_RF | 0.7901 | 0.7473 |
| Zscore_PCC_RFE_4_AB | 0.7856 | 0.8056 | Zscore_PCC_RFE_4_AB | 0.7856 | 0.8056 | Zscore_PCC_RFE_4_AB | 0.7856 | 0.8056 | Zscore_PCC_RFE_4_AB | 0.7856 | 0.7473 |
| Zscore_PCC_RFE_3_NB | 0.7839 | 0.6864 | Zscore_PCC_RFE_3_NB | 0.7839 | 0.6864 | Zscore_PCC_RFE_3_NB | 0.7839 | 0.6864 | Zscore_PCC_RFE_3_NB | 0.7839 | 0.6864 |
| Zscore_PCC_Relief_7_GP | 0.7739 | 0.7333 | Zscore_PCC_Relief_7_GP | 0.7739 | 0.7333 | Zscore_PCC_Relief_7_GP | 0.7739 | 0.7333 | Zscore_PCC_Relief_7_GP | 0.7739 |  |
| Zscore_PCC_KW_10_AE | 0.7737 | 0.7297 | Zscore_PCC_KW_10_AE | 0.7737 | 0.7297 | Zscore_PCC_KW_10_AE | 0.7737 | 0.7297 | Zscore_PCC_KW_10_AE | 0.7737 | 0.7297 |

PCC = Pearson Correlation Coefficient ; KW = Kruskal-Wallis ; AE = AutoEncoder ; GP = Gaussian process ; RF = random forests ; RFE = recursive feature elimination ; LDA = linear discriminant analysis ; LR = logistic regression ; SVM = support vector machine ; ANOVA = analysis of variance ; LRLasso = logistic regression via Lasso

## Supplementary Figures


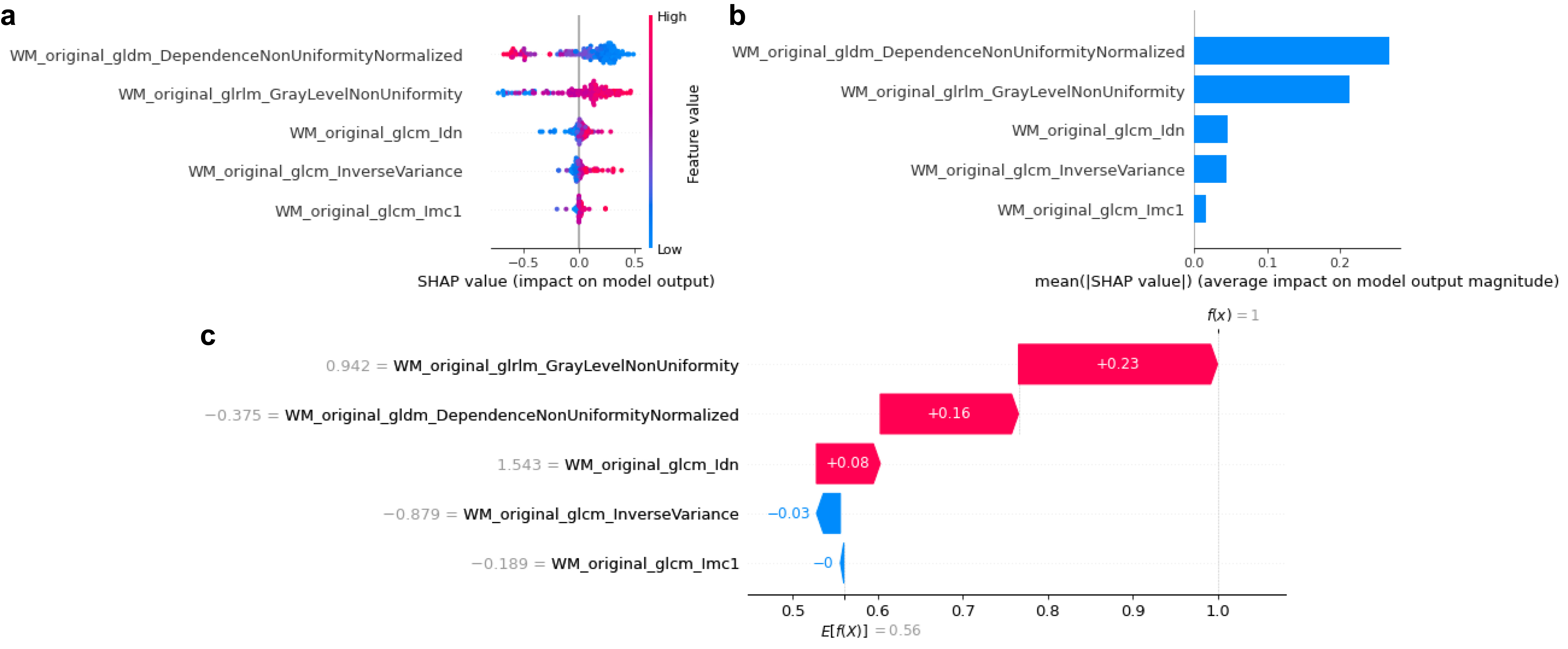


Figure 8. Model interpretability of the ANOVA5LDA model with SHapley Additive exPlanations (SHAP). a. Summary plot of feature impact on the decision of the model showing positive and negative relationships of the predictors with the target variable. A positive SHAP value indicates an increase in the probability of PD. b. Variance importance plot listing the most significant variables. The features that were more valuable for the diagnosis of PD were located at the top, presented in descending order. c.Waterfall diagram of the first sample in the ANOVA5LDA model. The waterfall diagram is designed to provide an explanation for a single prediction. It takes a single line of the interpreted object as input. The diagram begins with the expected value of the model output at the bottom. Each row then indicates whether each feature has a positive (red) or negative (blue) contribution. In other words, it shows how the value is pushed from the model's expected output value on the data set to the model's predicted output value.Note that ‘WM_original_glrlm_GrayLevelNonUniformity’ largely push the model distinction score higher.

.


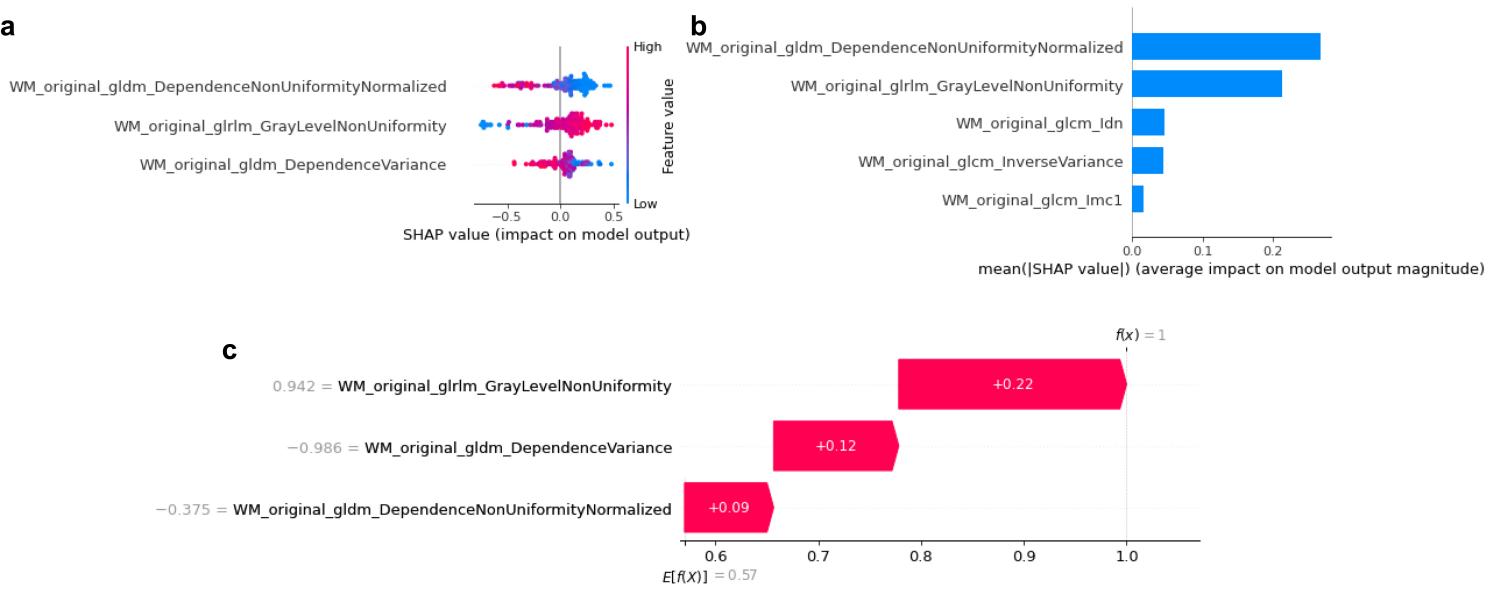


Figure 9. Model interpretability of the REF3LDA model with SHapley Additive exPlanations (SHAP) . a. Summary plot of feature impact on the decision of the model showing positive and negative relationships of the predictors with the target variable. A positive SHAP value indicates an increase in the probability of PD. b. Variance importance plot listing the most significant variables. The features that were more valuable for the diagnosis of PD were located at the top, presented in descending order. c.Waterfall diagram of the first sample in the REF3LDA model. The waterfall diagram is designed to provide an explanation for a single prediction. It takes a single line of the interpreted object as input. The diagram begins with the expected value of the model output at the bottom. Each row then indicates whether each feature has a positive (red) or negative (blue) contribution. In other words, it shows how the value is pushed from the model's expected output value on the data set to the model's predicted output value.It is worth noting that the most contributing factor is 'WM_original_glrlm_GrayLevelNonUniformity'.


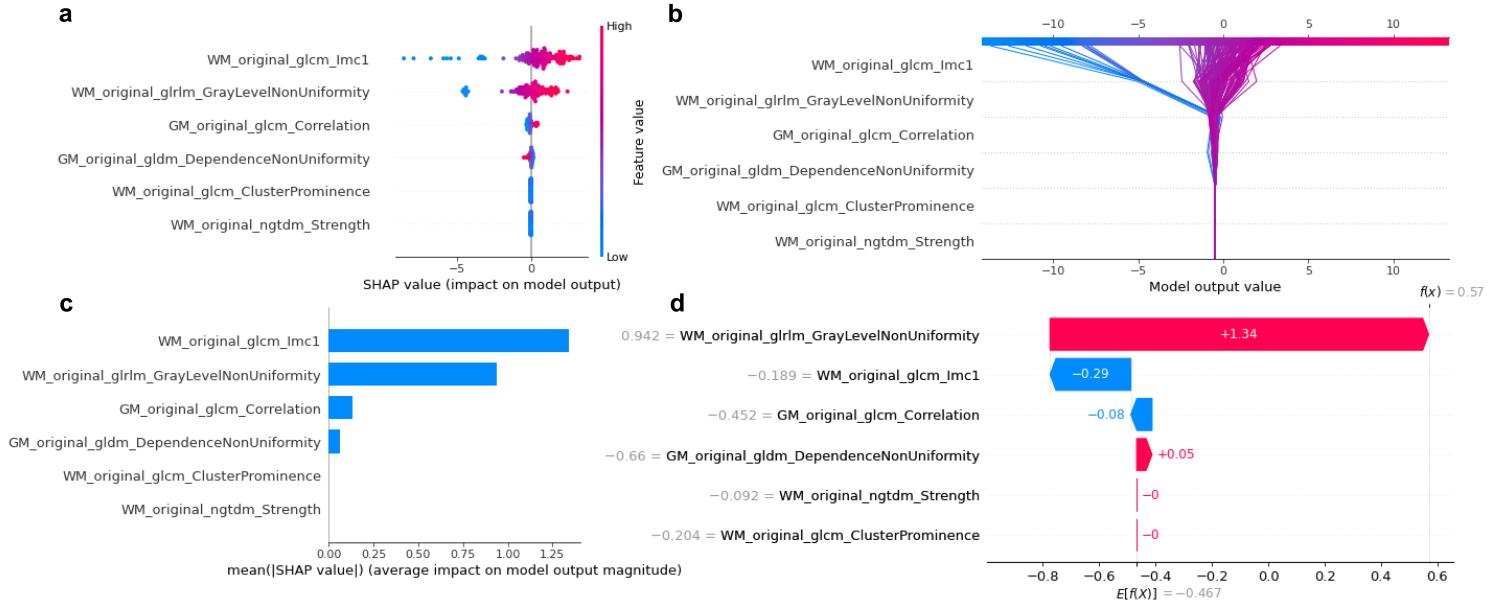


Figure 10. Model interpretability of the RELIEF6LRLasso model with SHapley Additive exPlanations (SHAP) . **a** . Summary plot of feature impact on the decision of the model showing positive and negative relationships of the predictors with the target variable. A positive SHAP value indicates an increase in the probability of PD. b. Decision diagram of the RELIEF6LRLasso model. The vertical gray line in the middle of the decision graph represents the basic value of the model. The colored line, on the other hand, indicates the prediction and shows whether each feature moves the output value above or below the average predicted value. The eigenvalues are positioned next to the prediction line for reference. Moving to the bottom of the diagram, the prediction line demonstrates the accumulation of SHAP value from the base value to the final score of the model at the top of the diagram. c. Variance importance plot listing the most significant variables. The features that were more valuable for the diagnosis of PD were located at the top, presented in descending order. **d**. Waterfall diagram of the first sample in the RELIEF6LRLasso model. The waterfall diagram is designed to provide an explanation for a single prediction. It takes a single line of the interpreted object as input. The diagram begins with the expected value of the model output at the bottom. Each row then indicates whether each feature has a positive (red) or negative (blue) contribution. In other words, it shows how the value is pushed from the model's expected output value on the data set to the model's predicted output value.It is worth noting that the most contributing factor is 'WM_original_glrlm_GrayLevelNonUniformity'.

**Table S1. Detailed classification of 73 image features.**

| **Type** |  |  |  |  |  |
| --- | --- | --- | --- | --- | --- |
| Grey Level Co-occurrence Matrix (GLCM) | Autocorrelation  ClusterProminence  ClusterShade  ClusterTendency  Contrast | Correlation  DifferenceAverage  DifferenceEntropy  DifferenceVariance  Id | Idm  Idmn  Idn  Imc1  Imc2 | InverseVariance  JointAverage  JointEnergy  JointEntropy  MaximumProbability | SumEntropy  SumSquares |
| Gray-level dependence matrix (GLDM) | DependenceEntropy  DependenceNonUniformity  DependenceNonUniformityNormalized  DependenceVariance  GrayLevelNonUniformity | GrayLevelVariance  HighGrayLevelEmphasis  LargeDependenceEmphasis  LargeDependenceHighGrayLevelEmphasis  LargeDependenceLowGrayLevelEmphasis | LowGrayLevelEmphasis  SmallDependenceEmphasis  SmallDependenceHighGrayLevelEmphasis  SmallDependenceLowGrayLevelEmphasis |  |  |
| Gray-level run length matrix (GLRLM) | GrayLevelNonUniformity  GrayLevelNonUniformityNormalized  GrayLevelVariance  HighGrayLevelRunEmphasis  LongRunEmphasis | LongRunHighGrayLevelEmphasis  LongRunLowGrayLevelEmphasis  LowGrayLevelRunEmphasis  RunEntropy  RunLengthNonUniformity | RunLengthNonUniformityNormalized  RunPercentage  RunVariance  ShortRunEmphasis  ShortRunHighGrayLevelEmphasis | ShortRunLowGrayLevelEmphasis |  |
| Gray-level size zone matrix (GLSZM) | GrayLevelNonUniformity  GrayLevelNonUniformityNormalized  GrayLevelVariance  HighGrayLevelZoneEmphasis  LargeAreaEmphasis | LargeAreaHighGrayLevelEmphasis  LargeAreaLowGrayLevelEmphasis  LowGrayLevelZoneEmphasis  SizeZoneNonUniformity  SizeZoneNonUniformityNormalized | SmallAreaEmphasis  SmallAreaHighGrayLevelEmphasis  SmallAreaLowGrayLevelEmphasis  ZoneEntropy  ZonePercentage | ZoneVariance |  |
| Neighboring gray tone difference matrix (NGTDM) | Busyness  Coarseness  Complexity  Contrast  Strength |  |  |  |  |

Note: Detailed meanings and formulas for each feature can be found at the URL (https://pyradiomics.readthedocs.io/en/latest/
