# Supplementary material for: A more objective PD diagnostic model: integrating texture feature markers of cerebellar gray matter and white matter through machine learning
Source: Front Aging Neurosci. 2024 Jun 7;16:1393841. doi: 10.3389/fnagi.2024.1393841 (PMC11190310; doi:10.3389/fnagi.2024.1393841)
Supplement: Supplementary file 2 [file Data_Sheet_2.pdf]

Train_results

	AUC	95% CIs	Std	AUC-PR	Cutoff	MCC	Acc	Youden Index	Sen	Spe	PPV	NPV
Zscore_PCC_ANOVA_1_SVM	0.7567	[0.7239-0.7894]	0.0167	0.7434	0.5477	0.4123	0.7034	1.1618	0.6225	0.7843	0.7427	0.6751
Zscore_PCC_ANOVA_1_LDA	0.7546	[0.7217-0.7875]	0.0168	0.7354	0.5653	0.4149	0.7022	1.223	0.5907	0.8137	0.7603	0.6653
Zscore_PCC_ANOVA_1_AE	0.74	[0.7059-0.7742]	0.0174	0.7297	0.5267	0.4092	0.7034	1.1078	0.6495	0.7574	0.728	0.6836
Zscore_PCC_ANOVA_1_RF	1	[nan-nan]	0	1	0.585	1	1	1	1	1	1	1
Zscore_PCC_ANOVA_1_LR	0.7568	[0.7240-0.7896]	0.0167	0.7436	0.5656	0.4189	0.7047	1.2132	0.598	0.8113	0.7601	0.6687
Zscore_PCC_ANOVA_1_LRLasso	0.7565	[0.7237-0.7893]	0.0167	0.7429	0.569	0.4144	0.7022	1.2181	0.5931	0.8113	0.7586	0.666
Zscore_PCC_ANOVA_1_AB	0.9219	[0.9050-0.9387]	0.0086	0.927	0.4984	0.6653	0.8321	0.9436	0.8603	0.8039	0.8144	0.8519
Zscore_PCC_ANOVA_1_DT	1	[nan-nan]	0	1	1	1	1	1	1	1	1	1
Zscore_PCC_ANOVA_1_GP	0.7632	[0.7310-0.7953]	0.0164	0.7694	0.5836	0.4253	0.7059	1.25	0.5809	0.8309	0.7745	0.6647
Zscore_PCC_ANOVA_1_NB	0.7434	[0.7100-0.7768]	0.017	0.7313	0.6054	0.3742	0.6863	1.0931	0.6397	0.7328	0.7054	0.6704
Zscore_PCC_ANOVA_2_SVM	0.817	[0.7885-0.8455]	0.0145	0.8224	0.5685	0.5081	0.7525	1.1127	0.6961	0.8088	0.7845	0.7269
Zscore_PCC_ANOVA_2_LDA	0.8077	[0.7784-0.8369]	0.0149	0.805	0.5665	0.4833	0.7414	1.0417	0.7206	0.7623	0.7519	0.7318
Zscore_PCC_ANOVA_2_AE	0.7986	[0.7685-0.8288]	0.0154	0.7828	0.4707	0.473	0.7365	1.0025	0.7353	0.7377	0.7371	0.7359
Zscore_PCC_ANOVA_2_RF	1	[nan-nan]	0	1	0.59	1	1	1	1	1	1	1
Zscore_PCC_ANOVA_2_LR	0.8175	[0.7891-0.8459]	0.0145	0.8265	0.5711	0.5024	0.75	1.098	0.701	0.799	0.7772	0.7277
Zscore_PCC_ANOVA_2_LRLasso	0.8172	[0.7888-0.8456]	0.0145	0.8254	0.5705	0.5041	0.7512	1.0809	0.7108	0.7917	0.7733	0.7324
Zscore_PCC_ANOVA_2_AB	0.9722	[0.9637-0.9807]	0.0043	0.9729	0.4992	0.8096	0.9044	0.9559	0.9265	0.8824	0.8873	0.9231
Zscore_PCC_ANOVA_2_DT	1	[nan-nan]	0	1	1	1	1	1	1	1	1	1
Zscore_PCC_ANOVA_2_GP	0.8306	[0.8030-0.8582]	0.0141	0.8318	0.5366	0.5159	0.7574	1.0686	0.723	0.7917	0.7763	0.7408
Zscore_PCC_ANOVA_2_NB	0.7816	[0.7501-0.8130]	0.016	0.7617	0.7491	0.4835	0.7377	1.1814	0.6471	0.8284	0.7904	0.7012
Zscore_PCC_ANOVA_3_SVM	0.8273	[0.7998-0.8549]	0.0141	0.8336	0.5803	0.5117	0.7537	1.1299	0.6887	0.8186	0.7915	0.7245
Zscore_PCC_ANOVA_3_LDA	0.8089	[0.7798-0.8380]	0.0148	0.8102	0.5675	0.4936	0.7463	1.0613	0.7157	0.777	0.7624	0.7321
Zscore_PCC_ANOVA_3_AE	0.7737	[0.7419-0.8054]	0.0162	0.7528	0.4873	0.432	0.7145	0.8799	0.7745	0.6544	0.6915	0.7437
Zscore_PCC_ANOVA_3_RF	1	[nan-nan]	0	1	0.58	1	1	1	1	1	1	1
Zscore_PCC_ANOVA_3_LR	0.8279	[0.8004-0.8553]	0.014	0.8359	0.5703	0.5027	0.75	1.1029	0.6985	0.8015	0.7787	0.7267
Zscore_PCC_ANOVA_3_LRLasso	0.8276	[0.8001-0.8551]	0.014	0.8357	0.5439	0.5051	0.7525	1.0245	0.7402	0.7647	0.7588	0.7464
Zscore_PCC_ANOVA_3_AB	0.9881	[0.9833-0.9928]	0.0024	0.9887	0.5004	0.8898	0.9449	1.0123	0.9387	0.951	0.9504	0.9395
Zscore_PCC_ANOVA_3_DT	1	[nan-nan]	0	1	1	1	1	1	1	1	1	1
Zscore_PCC_ANOVA_3_GP	0.8509	[0.8250-0.8767]	0.0132	0.8517	0.5095	0.5539	0.777	1	0.777	0.777	0.777	0.777
Zscore_PCC_ANOVA_3_NB	0.789	[0.7584-0.8197]	0.0156	0.7825	0.7841	0.467	0.7328	1.0735	0.6961	0.7696	0.7513	0.7169
Zscore_PCC_ANOVA_4_SVM	0.8487	[0.8230-0.8744]	0.0131	0.846	0.5347	0.5393	0.7696	1.0196	0.7598	0.7794	0.775	0.7644
Zscore_PCC_ANOVA_4_LDA	0.8324	[0.8052-0.8595]	0.0138	0.8241	0.5635	0.5221	0.761	1.0123	0.7549	0.7672	0.7643	0.7579
Zscore_PCC_ANOVA_4_AE	0.7379	[0.7045-0.7712]	0.017	0.7466	0.4743	0.3476	0.6703	1.1985	0.5711	0.7696	0.7125	0.6421
Zscore_PCC_ANOVA_4_RF	1	[nan-nan]	0	1	0.635	1	1	1	1	1	1	1
Zscore_PCC_ANOVA_4_LR	0.8474	[0.8216-0.8733]	0.0132	0.8436	0.5299	0.5419	0.7708	1.027	0.7574	0.7843	0.7783	0.7637
Zscore_PCC_ANOVA_4_LRLasso	0.8468	[0.8209-0.8727]	0.0132	0.8433	0.5242	0.527	0.7635	0.9975	0.7647	0.7623	0.7628	0.7641
Zscore_PCC_ANOVA_4_AB	0.9958	[0.9935-0.9982]	0.0012	0.9962	0.503	0.9353	0.9669	1.0564	0.9387	0.9951	0.9948	0.942
Zscore_PCC_ANOVA_4_DT	1	[nan-nan]	0	1	1	1	1	1	1	1	1	1
Zscore_PCC_ANOVA_4_GP	0.8714	[0.8475-0.8954]	0.0122	0.865	0.4822	0.6056	0.8015	0.9069	0.848	0.7549	0.7758	0.8324
Zscore_PCC_ANOVA_4_NB	0.7904	[0.7598-0.8209]	0.0156	0.7784	0.8382	0.4559	0.7279	1.0098	0.723	0.7328	0.7302	0.7257
Zscore_PCC_ANOVA_5_SVM	0.8654	[0.8414-0.8895]	0.0123	0.8645	0.4458	0.5737	0.7855	0.9044	0.8333	0.7377	0.7606	0.8157
Zscore_PCC_ANOVA_5_LDA	0.8544	[0.8293-0.8795]	0.0128	0.8509	0.5371	0.5469	0.7733	1.0368	0.7549	0.7917	0.7837	0.7636
Zscore_PCC_ANOVA_5_AE	0.8045	[0.7749-0.8340]	0.0151	0.8142	0.4895	0.4942	0.7451	1.1275	0.6814	0.8088	0.7809	0.7174
Zscore_PCC_ANOVA_5_RF	1	[nan-nan]	0	1	0.61	1	1	1	1	1	1	1
Zscore_PCC_ANOVA_5_LR	0.8652	[0.8412-0.8892]	0.0122	0.8643	0.5109	0.5638	0.7819	1.0196	0.7721	0.7917	0.7875	0.7764
Zscore_PCC_ANOVA_5_LRLasso	0.8646	[0.8405-0.8886]	0.0123	0.8635	0.5212	0.5614	0.7806	1.0172	0.7721	0.7892	0.7855	0.7759
Zscore_PCC_ANOVA_5_AB	0.998	[0.9965-0.9994]	0.0007	0.998	0.5017	0.9563	0.9779	1.0294	0.9632	0.9926	0.9924	0.9643
Zscore_PCC_ANOVA_5_DT	1	[nan-nan]	0	1	1	1	1	1	1	1	1	1
Zscore_PCC_ANOVA_5_GP	0.89	[0.8681-0.9120]	0.0112	0.8839	0.5106	0.6398	0.8199	0.9877	0.826	0.8137	0.816	0.8238
Zscore_PCC_ANOVA_5_NB	0.7942	[0.7640-0.8244]	0.0154	0.7912	0.9111	0.4832	0.7402	1.1078	0.6863	0.7941	0.7692	0.7168
Zscore_PCC_ANOVA_6_SVM	0.8711	[0.8474-0.8948]	0.0121	0.8645	0.5268	0.5964	0.7978	1.0515	0.7721	0.8235	0.814	0.7832
Zscore_PCC_ANOVA_6_LDA	0.8714	[0.8478-0.8950]	0.012	0.8658	0.5414	0.6001	0.799	1.0833	0.7574	0.8407	0.8262	0.776
Zscore_PCC_ANOVA_6_AE	0.7877	[0.7570-0.8184]	0.0157	0.8056	0.4938	0.4623	0.7279	1.1667	0.6446	0.8113	0.7735	0.6954
Zscore_PCC_ANOVA_6_RF	1	[nan-nan]	0	1	0.645	1	1	1	1	1	1	1
Zscore_PCC_ANOVA_6_LR	0.8713	[0.8477-0.8948]	0.012	0.865	0.4849	0.5882	0.7941	0.9951	0.7966	0.7917	0.7927	0.7956
Zscore_PCC_ANOVA_6_LRLasso	0.8709	[0.8474-0.8945]	0.012	0.8653	0.5313	0.5886	0.7941	1.0343	0.777	0.8113	0.8046	0.7844
Zscore_PCC_ANOVA_6_AB	0.9995	[0.9990-0.9999]	0.0002	0.9995	0.4992	0.9755	0.9877	0.9902	0.9926	0.9828	0.983	0.9926
Zscore_PCC_ANOVA_6_DT	1	[nan-nan]	0	1	1	1	1	1	1	1	1	1
Zscore_PCC_ANOVA_6_GP	0.8972	[0.8758-0.9185]	0.0109	0.887	0.45	0.6706	0.8309	0.8382	0.9118	0.75	0.7848	0.8947
Zscore_PCC_ANOVA_6_NB	0.8008	[0.7710-0.8306]	0.0152	0.7902	0.9126	0.4706	0.7353	1.0147	0.7279	0.7426	0.7388	0.7319
Zscore_PCC_ANOVA_7_SVM	0.8748	[0.8514-0.8981]	0.0119	0.8675	0.5265	0.6059	0.8027	1.0417	0.7819	0.8235	0.8159	0.7906
Zscore_PCC_ANOVA_7_LDA	0.8758	[0.8526-0.8989]	0.0118	0.8692	0.5153	0.6114	0.8051	1.0613	0.7745	0.8358	0.8251	0.7875
Zscore_PCC_ANOVA_7_AE	0.7489	[0.7157-0.7821]	0.0169	0.7296	0.5372	0.3984	0.69	0.6985	0.8407	0.5392	0.646	0.7719
Zscore_PCC_ANOVA_7_RF	1	[nan-nan]	0	1	0.63	1	1	1	1	1	1	1
Zscore_PCC_ANOVA_7_LR	0.8752	[0.8521-0.8983]	0.0118	0.8695	0.4178	0.6022	0.799	0.8824	0.8578	0.7402	0.7675	0.8389
Zscore_PCC_ANOVA_7_LRLasso	0.8749	[0.8518-0.8980]	0.0118	0.8692	0.4847	0.5909	0.7953	0.973	0.8088	0.7819	0.7876	0.8035
Zscore_PCC_ANOVA_7_AB	0.9996	[0.9992-1.0000]	0.0002	0.9996	0.5008	0.9755	0.9877	1	0.9877	0.9877	0.9877	0.9877
Zscore_PCC_ANOVA_7_DT	1	[nan-nan]	0	1	1	1	1	1	1	1	1	1
Zscore_PCC_ANOVA_7_GP	0.9109	[0.8911-0.9308]	0.0101	0.9031	0.4963	0.7142	0.8566	0.9485	0.8824	0.8309	0.8392	0.876
Zscore_PCC_ANOVA_7_NB	0.8048	[0.7751-0.8345]	0.0151	0.7908	0.8918	0.498	0.7488	0.9583	0.7696	0.7279	0.7388	0.7596
Zscore_PCC_ANOVA_8_SVM	0.8788	[0.8560-0.9016]	0.0116	0.8742	0.5215	0.6235	0.8113	1.0539	0.7843	0.8382	0.829	0.7953

Zscore_PCC_ANOVA_8_LDA	0.8794 [0.8567-0.9021]	0.0116	0.8739	0.5152	0.6211	0.81	1.0564	0.7819	0.8382	0.8286	0.7935
Zscore_PCC_ANOVA_8_AE	0.7242 [0.6894-0.7590]	0.0177	0.6878	0.5064	0.3851	0.6924	0.9632	0.7108	0.674	0.6856	0.6997
Zscore_PCC_ANOVA_8_RF	1 [nan-nan]	0	1	0.62	1	1	1	1	1	1	1
Zscore_PCC_ANOVA_8_LR	0.8798 [0.8573-0.9023]	0.0115	0.8751	0.5155	0.6062	0.8027	1.0515	0.777	0.8284	0.8191	0.7879
Zscore_PCC_ANOVA_8_LRLasso	0.8782 [0.8555-0.9009]	0.0116	0.8734	0.5335	0.6065	0.8027	1.0613	0.7721	0.8333	0.8225	0.7852
Zscore_PCC_ANOVA_8_AB	0.9998 [0.9996-1.0000]	0.0001	0.9998	0.5009	0.9878	0.9939	1.0074	0.9902	0.9975	0.9975	0.9903
Zscore_PCC_ANOVA_8_DT	1 [nan-nan]	0	1	1	1	1	1	1	1	1	1
Zscore_PCC_ANOVA_8_GP	0.9257 [0.9080-0.9435]	0.009	0.9224	0.4662	0.751	0.8738	0.9044	0.9216	0.826	0.8412	0.9133
Zscore_PCC_ANOVA_8_NB	0.8074 [0.7782-0.8367]	0.0149	0.8107	0.9502	0.4945	0.7463	1.0858	0.7034	0.7892	0.7694	0.7269
Zscore_PCC_ANOVA_9_SVM	0.8808 [0.8581-0.9035]	0.0116	0.8743	0.5261	0.6331	0.8162	1.049	0.7917	0.8407	0.8325	0.8014
Zscore_PCC_ANOVA_9_LDA	0.8821 [0.8597-0.9045]	0.0114	0.876	0.516	0.6311	0.815	1.0613	0.7843	0.8456	0.8355	0.7968
Zscore_PCC_ANOVA_9_AE	0.8063 [0.7766-0.8360]	0.0152	0.8019	0.4904	0.4986	0.7488	0.9338	0.7819	0.7157	0.7333	0.7664
Zscore_PCC_ANOVA_9_RF	1 [nan-nan]	0	1	0.655	1	1	1	1	1	1	1
Zscore_PCC_ANOVA_9_LR	0.8812 [0.8587-0.9036]	0.0114	0.8753	0.4316	0.6144	0.8064	0.9265	0.8431	0.7696	0.7854	0.8307
Zscore_PCC_ANOVA_9_LRLasso	0.8798 [0.8573-0.9024]	0.0115	0.8747	0.5335	0.6038	0.8015	1.0539	0.7745	0.8284	0.8187	0.786
Zscore_PCC_ANOVA_9_AB	1 [0.9999-1.0000]	0	1	0.4998	0.9951	0.9975	1	0.9975	0.9975	0.9975	0.9975
Zscore_PCC_ANOVA_9_DT	1 [nan-nan]	0	1	1	1	1	1	1	1	1	1
Zscore_PCC_ANOVA_9_GP	0.9363 [0.9201-0.9524]	0.0082	0.9342	0.4724	0.7756	0.886	0.9044	0.9338	0.8382	0.8523	0.9268
Zscore_PCC_ANOVA_9_NB	0.8074 [0.7781-0.8368]	0.015	0.8086	0.935	0.4976	0.7488	0.9828	0.7574	0.7402	0.7446	0.7531
Zscore_PCC_ANOVA_10_SVM	0.8823 [0.8598-0.9048]	0.0115	0.8761	0.523	0.6304	0.815	1.0417	0.7941	0.8358	0.8286	0.8024
Zscore_PCC_ANOVA_10_LDA	0.8829 [0.8606-0.9053]	0.0114	0.8768	0.4964	0.6302	0.815	1.0319	0.799	0.8309	0.8253	0.8052
Zscore_PCC_ANOVA_10_AE	0.7654 [0.7331-0.7976]	0.0165	0.7391	0.5139	0.4341	0.7157	0.8873	0.7721	0.6593	0.6938	0.7431
Zscore_PCC_ANOVA_10_RF	1 [nan-nan]	0	1	0.665	1	1	1	1	1	1	1
Zscore_PCC_ANOVA_10_LR	0.8814 [0.8590-0.9037]	0.0114	0.8762	0.4277	0.6146	0.8064	0.9216	0.8456	0.7672	0.7841	0.8324
Zscore_PCC_ANOVA_10_LRLasso	0.8801 [0.8576-0.9025]	0.0115	0.875	0.4677	0.6013	0.8002	0.9485	0.826	0.7745	0.7855	0.8165
Zscore_PCC_ANOVA_10_AB	0.9999 [0.9998-1.0000]	0.0001	0.9999	0.5032	0.9902	0.9951	1.0098	0.9902	1	1	0.9903
Zscore_PCC_ANOVA_10_DT	1 [nan-nan]	0	1	1	1	1	1	1	1	1	1
Zscore_PCC_ANOVA_10_GP	0.9443 [0.9293-0.9593]	0.0077	0.9411	0.4593	0.7862	0.8909	0.8946	0.9436	0.8382	0.8537	0.937
Zscore_PCC_ANOVA_10_NB	0.8075 [0.7781-0.8369]	0.015	0.8096	0.9557	0.5074	0.7537	1.0025	0.7525	0.7549	0.7543	0.7531
Zscore_PCC_ANOVA_11_SVM	0.8834 [0.8610-0.9058]	0.0114	0.8769	0.5274	0.6328	0.8162	1.0392	0.7966	0.8358	0.8291	0.8042
Zscore_PCC_ANOVA_11_LDA	0.8832 [0.8609-0.9054]	0.0114	0.8762	0.499	0.6303	0.815	1.0368	0.7966	0.8333	0.827	0.8038
Zscore_PCC_ANOVA_11_AE	0.7658 [0.7338-0.7978]	0.0163	0.757	0.5363	0.4395	0.7145	0.7819	0.8235	0.6054	0.6761	0.7743
Zscore_PCC_ANOVA_11_RF	1 [nan-nan]	0	1	0.65	1	1	1	1	1	1	1
Zscore_PCC_ANOVA_11_LR	0.8809 [0.8585-0.9033]	0.0114	0.8753	0.4299	0.6071	0.8027	0.924	0.8407	0.7647	0.7813	0.8276
Zscore_PCC_ANOVA_11_LRLasso	0.8801 [0.8576-0.9026]	0.0115	0.8751	0.4677	0.6013	0.8002	0.9485	0.826	0.7745	0.7855	0.8165
Zscore_PCC_ANOVA_11_AB	1 [0.9999-1.0000]	0	1	0.5022	0.9951	0.9975	1.0049	0.9951	1	1	0.9951
Zscore_PCC_ANOVA_11_DT	1 [nan-nan]	0	1	1	1	1	1	1	1	1	1
Zscore_PCC_ANOVA_11_GP	0.9478 [0.9333-0.9624]	0.0074	0.9444	0.4637	0.797	0.8958	0.8848	0.9534	0.8382	0.8549	0.9474
Zscore_PCC_ANOVA_11_NB	0.8014 [0.7716-0.8313]	0.0152	0.8025	0.9851	0.4973	0.7475	1.0931	0.701	0.7941	0.773	0.7265
Zscore_PCC_ANOVA_12_SVM	0.8866 [0.8645-0.9087]	0.0113	0.8813	0.5335	0.6382	0.8186	1.0539	0.7917	0.8456	0.8368	0.8023
Zscore_PCC_ANOVA_12_LDA	0.8883 [0.8665-0.9101]	0.0111	0.8834	0.4923	0.6496	0.8248	1.0172	0.8162	0.8333	0.8304	0.8193
Zscore_PCC_ANOVA_12_AE	0.7912 [0.7607-0.8218]	0.0156	0.7857	0.5215	0.4804	0.7377	0.8578	0.8088	0.6667	0.7082	0.7771
Zscore_PCC_ANOVA_12_RF	1 [nan-nan]	0	1	0.64	1	1	1	1	1	1	1
Zscore_PCC_ANOVA_12_LR	0.8822 [0.8600-0.9044]	0.0113	0.8774	0.4242	0.6268	0.8125	0.924	0.8505	0.7745	0.7904	0.8382
Zscore_PCC_ANOVA_12_LRLasso	0.8837 [0.8615-0.9058]	0.0113	0.8794	0.504	0.6103	0.8051	0.9975	0.8064	0.8039	0.8044	0.8059
Zscore_PCC_ANOVA_12_AB	1 [nan-nan]	0	1	0.501	1	1	1	1	1	1	1
Zscore_PCC_ANOVA_12_DT	1 [nan-nan]	0	1	1	1	1	1	1	1	1	1
Zscore_PCC_ANOVA_12_GP	0.9543 [0.9409-0.9677]	0.0068	0.9527	0.4678	0.815	0.9056	0.9044	0.9534	0.8578	0.8702	0.9485
Zscore_PCC_ANOVA_12_NB	0.7979 [0.7676-0.8281]	0.0154	0.7894	0.9947	0.4986	0.7475	1.1176	0.6887	0.8064	0.7806	0.7215
Zscore_PCC_ANOVA_13_SVM	0.8881 [0.8662-0.9100]	0.0112	0.8827	0.5326	0.6281	0.8137	1.0441	0.7917	0.8358	0.8282	0.8005
Zscore_PCC_ANOVA_13_LDA	0.8889 [0.8672-0.9106]	0.0111	0.8844	0.5173	0.6403	0.8199	1.0417	0.799	0.8407	0.8338	0.8071
Zscore_PCC_ANOVA_13_AE	0.7935 [0.7628-0.8241]	0.0156	0.7745	0.4993	0.4978	0.7488	0.9681	0.7647	0.7328	0.7411	0.757
Zscore_PCC_ANOVA_13_RF	1 [nan-nan]	0	1	0.675	1	1	1	1	1	1	1
Zscore_PCC_ANOVA_13_LR	0.8834 [0.8613-0.9055]	0.0113	0.8787	0.428	0.6168	0.8076	0.9289	0.8431	0.7721	0.7872	0.8311
Zscore_PCC_ANOVA_13_LRLasso	0.885 [0.8631-0.9070]	0.0112	0.8808	0.3926	0.6215	0.8051	0.8113	0.8995	0.7108	0.7567	0.8761
Zscore_PCC_ANOVA_13_AB	1 [nan-nan]	0	1	0.5	1	1	1	1	1	1	1
Zscore_PCC_ANOVA_13_DT	1 [nan-nan]	0	1	1	1	1	1	1	1	1	1
Zscore_PCC_ANOVA_13_GP	0.9616 [0.9494-0.9738]	0.0062	0.9596	0.4661	0.8298	0.913	0.9044	0.9608	0.8652	0.877	0.9566
Zscore_PCC_ANOVA_13_NB	0.7927 [0.7621-0.8234]	0.0156	0.7843	0.9946	0.4834	0.7414	1.0466	0.7181	0.7647	0.7532	0.7307
Zscore_PCC_ANOVA_14_SVM	0.89 [0.8682-0.9117]	0.0111	0.8826	0.5454	0.6245	0.8113	1.0784	0.7721	0.8505	0.8378	0.7886
Zscore_PCC_ANOVA_14_LDA	0.892 [0.8707-0.9133]	0.0109	0.8876	0.5044	0.6326	0.8162	1.0294	0.8015	0.8309	0.8258	0.8071
Zscore_PCC_ANOVA_14_AE	0.613 [0.5742-0.6518]	0.0198	0.5475	0.6575	0.2343	0.6115	0.6936	0.7647	0.4583	0.5854	0.6608
Zscore_PCC_ANOVA_14_RF	1 [nan-nan]	0	1	0.66	1	1	1	1	1	1	1
Zscore_PCC_ANOVA_14_LR	0.8845 [0.8626-0.9064]	0.0112	0.8804	0.5358	0.6074	0.8027	1.0809	0.7623	0.8431	0.8293	0.78
Zscore_PCC_ANOVA_14_LRLasso	0.8855 [0.8636-0.9074]	0.0112	0.8816	0.5335	0.6185	0.8088	1.0539	0.7819	0.8358	0.8264	0.793
Zscore_PCC_ANOVA_14_AB	1 [nan-nan]	0	1	0.5001	1	1	1	1	1	1	1
Zscore_PCC_ANOVA_14_DT	1 [nan-nan]	0	1	1	1	1	1	1	1	1	1
Zscore_PCC_ANOVA_14_GP	0.9668 [0.9554-0.9783]	0.0059	0.9646	0.479	0.8474	0.9228	0.9338	0.9559	0.8897	0.8966	0.9528
Zscore_PCC_ANOVA_14_NB	0.7938 [0.7633-0.8243]	0.0156	0.7896	0.9965	0.4814	0.7402	1.0637	0.7083	0.7721	0.7565	0.7258
Zscore_PCC_ANOVA_15_SVM	0.8937 [0.8724-0.9151]	0.0109	0.8844	0.5	0.6397	0.8199	0.9975	0.8211	0.8186	0.8191	0.8206
Zscore_PCC_ANOVA_15_LDA	0.8985 [0.8780-0.9190]	0.0105	0.8927	0.4105	0.6551	0.826	0.902	0.875	0.777	0.7969	0.8614
Zscore_PCC_ANOVA_15_AE	0.7804 [0.7490-0.8117]	0.016	0.7696	0.5182	0.475	0.7365	0.9093	0.7819	0.6912	0.7169	0.7601
Zscore_PCC_ANOVA_15_RF	1 [nan-nan]	0	1	0.655	1	1	1	1	1	1	1

Zscore_PCC_ANOVA_15_LR	0.8873 [0.8656-0.9089]	0.011	0.8818	0.4145	0.6147	0.8051	0.8799	0.8652	0.7451	0.7724	0.8468
Zscore_PCC_ANOVA_15_LRLasso	0.8878 [0.8662-0.9095]	0.011	0.8824	0.5335	0.6159	0.8076	1.0466	0.7843	0.8309	0.8226	0.7939
Zscore_PCC_ANOVA_15_AB	1 [nan-nan]	0	1	0.5034	1	1	1	1	1	1	1
Zscore_PCC_ANOVA_15_DT	1 [nan-nan]	0	1	1	1	1	1	1	1	1	1
Zscore_PCC_ANOVA_15_GP	0.9684 [0.9571-0.9796]	0.0057	0.9666	0.5094	0.8554	0.9277	0.9926	0.9314	0.924	0.9246	0.9309
Zscore_PCC_ANOVA_15_NB	0.7881 [0.7573-0.8190]	0.0157	0.785	0.998	0.4659	0.7328	1.0294	0.7181	0.7475	0.7399	0.7262
Zscore_PCC_ANOVA_16_SVM	0.8952 [0.8740-0.9165]	0.0109	0.883	0.4364	0.643	0.8199	0.8995	0.8701	0.7696	0.7906	0.8556
Zscore_PCC_ANOVA_16_LDA	0.8993 [0.8790-0.9197]	0.0104	0.8923	0.4342	0.6529	0.826	0.9461	0.8529	0.799	0.8093	0.8446
Zscore_PCC_ANOVA_16_AE	0.7815 [0.7501-0.8129]	0.016	0.7605	0.5031	0.4524	0.7255	1.0784	0.6863	0.7647	0.7447	0.7091
Zscore_PCC_ANOVA_16_RF	1 [nan-nan]	0	1	0.655	1	1	1	1	1	1	1
Zscore_PCC_ANOVA_16_LR	0.8886 [0.8670-0.9102]	0.011	0.8808	0.3976	0.6204	0.8064	0.8431	0.8848	0.7279	0.7648	0.8634
Zscore_PCC_ANOVA_16_LRLasso	0.8891 [0.8675-0.9106]	0.011	0.8821	0.3805	0.6304	0.8076	0.7819	0.9167	0.6985	0.7525	0.8934
Zscore_PCC_ANOVA_16_AB	1 [nan-nan]	0	1	0.5034	1	1	1	1	1	1	1
Zscore_PCC_ANOVA_16_DT	1 [nan-nan]	0	1	1	1	1	1	1	1	1	1
Zscore_PCC_ANOVA_16_GP	0.9739 [0.9639-0.9839]	0.0051	0.9718	0.4976	0.8682	0.9338	0.9657	0.951	0.9167	0.9194	0.9492
Zscore_PCC_ANOVA_16_NB	0.7772 [0.7457-0.8088]	0.0161	0.7674	0.9993	0.4296	0.7145	1.0564	0.6863	0.7426	0.7273	0.703
Zscore_PCC_ANOVA_17_SVM	0.8957 [0.8745-0.9169]	0.0108	0.8856	0.4901	0.6423	0.8211	0.9804	0.8309	0.8113	0.8149	0.8275
Zscore_PCC_ANOVA_17_LDA	0.9009 [0.8808-0.9210]	0.0103	0.8948	0.4496	0.6453	0.8223	0.9534	0.8456	0.799	0.808	0.838
Zscore_PCC_ANOVA_17_AE	0.8082 [0.7790-0.8373]	0.0149	0.8016	0.5611	0.478	0.739	0.9926	0.7426	0.7353	0.7372	0.7407
Zscore_PCC_ANOVA_17_RF	1 [nan-nan]	0	1	0.66	1	1	1	1	1	1	1
Zscore_PCC_ANOVA_17_LR	0.8888 [0.8673-0.9104]	0.011	0.8812	0.468	0.6156	0.8076	0.9632	0.826	0.7892	0.7967	0.8193
Zscore_PCC_ANOVA_17_LRLasso	0.8898 [0.8683-0.9113]	0.011	0.8829	0.4434	0.6172	0.8076	0.9191	0.848	0.7672	0.7846	0.8347
Zscore_PCC_ANOVA_17_AB	1 [nan-nan]	0	1	0.503	1	1	1	1	1	1	1
Zscore_PCC_ANOVA_17_DT	1 [nan-nan]	0	1	1	1	1	1	1	1	1	1
Zscore_PCC_ANOVA_17_GP	0.9778 [0.9686-0.9870]	0.0047	0.9757	0.518	0.8775	0.9387	1.0049	0.9363	0.9412	0.9409	0.9366
Zscore_PCC_ANOVA_17_NB	0.7631 [0.7307-0.7955]	0.0165	0.7462	0.9986	0.4321	0.7157	0.9412	0.7451	0.6863	0.7037	0.7292
Zscore_PCC_ANOVA_18_SVM	0.9013 [0.8808-0.9218]	0.0105	0.8922	0.4169	0.6569	0.8248	0.8505	0.8995	0.75	0.7825	0.8818
Zscore_PCC_ANOVA_18_LDA	0.9053 [0.8858-0.9248]	0.01	0.9012	0.4516	0.6527	0.826	0.951	0.8505	0.8015	0.8107	0.8428
Zscore_PCC_ANOVA_18_AE	0.7724 [0.7405-0.8043]	0.0163	0.7668	0.5067	0.4441	0.7218	0.9534	0.7451	0.6985	0.7119	0.7326
Zscore_PCC_ANOVA_18_RF	1 [nan-nan]	0	1	0.66	1	1	1	1	1	1	1
Zscore_PCC_ANOVA_18_LR	0.8918 [0.8706-0.9130]	0.0108	0.8859	0.3936	0.6276	0.81	0.8456	0.8873	0.7328	0.7686	0.8667
Zscore_PCC_ANOVA_18_LRLasso	0.8937 [0.8727-0.9147]	0.0107	0.8879	0.4309	0.6311	0.8137	0.8922	0.8676	0.7598	0.7832	0.8516
Zscore_PCC_ANOVA_18_AB	1 [nan-nan]	0	1	0.5038	1	1	1	1	1	1	1
Zscore_PCC_ANOVA_18_DT	1 [nan-nan]	0	1	1	1	1	1	1	1	1	1
Zscore_PCC_ANOVA_18_GP	0.9823 [0.9742-0.9904]	0.0041	0.9811	0.4923	0.8907	0.9449	0.9534	0.9681	0.9216	0.9251	0.9666
Zscore_PCC_ANOVA_18_NB	0.7576 [0.7251-0.7902]	0.0166	0.748	0.9997	0.4069	0.7034	1.0049	0.701	0.7059	0.7044	0.7024
Zscore_PCC_ANOVA_19_SVM	0.9033 [0.8832-0.9235]	0.0103	0.8952	0.5	0.652	0.826	1	0.826	0.826	0.826	0.826
Zscore_PCC_ANOVA_19_LDA	0.9073 [0.8881-0.9264]	0.0098	0.905	0.5048	0.652	0.826	1.0147	0.8186	0.8333	0.8308	0.8213
Zscore_PCC_ANOVA_19_AE	0.7858 [0.7548-0.8167]	0.0158	0.7775	0.4501	0.4615	0.7304	0.9461	0.7574	0.7034	0.7186	0.7435
Zscore_PCC_ANOVA_19_RF	1 [nan-nan]	0	1	0.675	1	1	1	1	1	1	1
Zscore_PCC_ANOVA_19_LR	0.8926 [0.8714-0.9138]	0.0108	0.8858	0.4041	0.6407	0.8174	0.8652	0.8848	0.75	0.7797	0.8669
Zscore_PCC_ANOVA_19_LRLasso	0.8943 [0.8733-0.9153]	0.0107	0.8881	0.4203	0.6407	0.8174	0.8652	0.8848	0.75	0.7797	0.8669
Zscore_PCC_ANOVA_19_AB	1 [nan-nan]	0	1	0.5033	1	1	1	1	1	1	1
Zscore_PCC_ANOVA_19_DT	1 [nan-nan]	0	1	1	1	1	1	1	1	1	1
Zscore_PCC_ANOVA_19_GP	0.9847 [0.9770-0.9923]	0.0039	0.9838	0.4976	0.9021	0.951	0.9804	0.9608	0.9412	0.9423	0.96
Zscore_PCC_ANOVA_19_NB	0.7601 [0.7277-0.7926]	0.0166	0.7499	0.9999	0.4056	0.7022	1.076	0.6642	0.7402	0.7188	0.6879
Zscore_PCC_ANOVA_20_SVM	0.9031 [0.8829-0.9233]	0.0103	0.8951	0.5154	0.6522	0.826	1.0245	0.8137	0.8382	0.8342	0.8182
Zscore_PCC_ANOVA_20_LDA	0.9088 [0.8898-0.9278]	0.0097	0.907	0.4642	0.662	0.8309	0.9755	0.8431	0.8186	0.823	0.8392
Zscore_PCC_ANOVA_20_AE	0.7626 [0.7301-0.7951]	0.0166	0.7247	0.5049	0.4401	0.7181	0.8676	0.7843	0.652	0.6926	0.7514
Zscore_PCC_ANOVA_20_RF	1 [nan-nan]	0	1	0.65	1	1	1	1	1	1	1
Zscore_PCC_ANOVA_20_LR	0.8926 [0.8714-0.9138]	0.0108	0.8855	0.4102	0.6341	0.815	0.8848	0.8725	0.7574	0.7824	0.856
Zscore_PCC_ANOVA_20_LRLasso	0.8948 [0.8738-0.9157]	0.0107	0.8884	0.445	0.6354	0.8162	0.902	0.8652	0.7672	0.7879	0.8505
Zscore_PCC_ANOVA_20_AB	1 [nan-nan]	0	1	0.504	1	1	1	1	1	1	1
Zscore_PCC_ANOVA_20_DT	1 [nan-nan]	0	1	1	1	1	1	1	1	1	1
Zscore_PCC_ANOVA_20_GP	0.9874 [0.9805-0.9943]	0.0035	0.9858	0.5115	0.9118	0.9559	1	0.9559	0.9559	0.9559	0.9559
Zscore_PCC_ANOVA_20_NB	0.7733 [0.7415-0.8052]	0.0162	0.7614	1	0.4247	0.712	1.0564	0.6838	0.7402	0.7247	0.7007
Zscore_PCC_KW_1_SVM	0.7745 [0.7427-0.8062]	0.0162	0.7486	0.5313	0.4363	0.7181	1.0147	0.7108	0.7255	0.7214	0.715
Zscore_PCC_KW_1_LDA	0.683 [0.6469-0.7192]	0.0184	0.6609	0.5231	0.2893	0.6446	1.0294	0.6299	0.6593	0.649	0.6405
Zscore_PCC_KW_1_AE	0.6125 [0.5731-0.6519]	0.0201	0.5805	0.5226	0.314	0.6458	0.6299	0.8309	0.4608	0.6064	0.7315
Zscore_PCC_KW_1_RF	1 [nan-nan]	0	1	0.555	1	1	1	1	1	1	1
Zscore_PCC_KW_1_LR	0.7499 [0.7167-0.7831]	0.0169	0.7192	0.5376	0.3942	0.6936	1.1863	0.6005	0.7868	0.738	0.6632
Zscore_PCC_KW_1_LRLasso	0.7499 [0.7167-0.7830]	0.0169	0.7202	0.5382	0.3779	0.6887	1.049	0.6642	0.7132	0.6985	0.6799
Zscore_PCC_KW_1_AB	0.9432 [0.9293-0.9570]	0.0071	0.9456	0.4995	0.7133	0.8566	1.0074	0.8529	0.8603	0.8593	0.854
Zscore_PCC_KW_1_DT	1 [nan-nan]	0	1	1	1	1	1	1	1	1	1
Zscore_PCC_KW_1_GP	0.7357 [0.7018-0.7696]	0.0173	0.7145	0.5355	0.3696	0.6789	1.25	0.5539	0.8039	0.7386	0.6431
Zscore_PCC_KW_1_NB	0.6299 [0.5918-0.6679]	0.0194	0.5809	0.9661	0.2163	0.6078	0.9265	0.6446	0.5711	0.6005	0.6164
Zscore_PCC_KW_2_SVM	0.7688 [0.7368-0.8008]	0.0163	0.7629	0.5687	0.4321	0.7083	1.2647	0.576	0.8407	0.7833	0.6647
Zscore_PCC_KW_2_LDA	0.7625 [0.7301-0.7949]	0.0165	0.755	0.5644	0.4169	0.7022	1.2426	0.5809	0.8235	0.767	0.6627
Zscore_PCC_KW_2_AE	0.6819 [0.6452-0.7186]	0.0187	0.7116	0.49	0.367	0.6642	1.4461	0.4412	0.8873	0.7965	0.6136
Zscore_PCC_KW_2_RF	1 [nan-nan]	0	1	0.585	1	1	1	1	1	1	1
Zscore_PCC_KW_2_LR	0.7682 [0.7361-0.8002]	0.0163	0.7629	0.5732	0.4315	0.7083	1.2598	0.5784	0.8382	0.7815	0.6654
Zscore_PCC_KW_2_LRLasso	0.7673 [0.7352-0.7994]	0.0164	0.762	0.5679	0.4222	0.7059	1.2206	0.5956	0.8162	0.7642	0.6687
Zscore_PCC_KW_2_AB	0.9664 [0.9568-0.9761]	0.0049	0.9694	0.5007	0.7784	0.8885	1.0613	0.8578	0.9191	0.9138	0.8661

Zscore_PCC_KW_2_DT	1 [nan-nan]	0	1	1	1	1	1	1	1	1	1
Zscore_PCC_KW_2_GP	0.7686 [0.7367-0.8005]	0.0163	0.7846	0.575	0.4368	0.7096	1.2819	0.5686	0.8505	0.7918	0.6635
Zscore_PCC_KW_2_NB	0.713 [0.6778-0.7481]	0.018	0.668	0.9853	0.3701	0.685	1.0074	0.6814	0.6887	0.6864	0.6837
Zscore_PCC_KW_3_SVM	0.8223 [0.7942-0.8504]	0.0143	0.8262	0.5164	0.5074	0.7537	0.9828	0.7623	0.7451	0.7494	0.7581
Zscore_PCC_KW_3_LDA	0.8071 [0.7776-0.8365]	0.015	0.8056	0.5502	0.4853	0.7426	1.0147	0.7353	0.75	0.7463	0.7391
Zscore_PCC_KW_3_AE	0.7968 [0.7664-0.8271]	0.0155	0.7777	0.4921	0.4841	0.7414	0.9289	0.777	0.7059	0.7254	0.7599
Zscore_PCC_KW_3_RF	1 [nan-nan]	0	1	0.575	1	1	1	1	1	1	1
Zscore_PCC_KW_3_LR	0.8217 [0.7936-0.8499]	0.0144	0.8258	0.5439	0.5076	0.7537	1.0319	0.7377	0.7696	0.762	0.7458
Zscore_PCC_KW_3_LRLasso	0.8217 [0.7936-0.8499]	0.0144	0.8261	0.5505	0.5052	0.7525	1.0343	0.7353	0.7696	0.7614	0.7441
Zscore_PCC_KW_3_AB	0.9901 [0.9860-0.9941]	0.0021	0.9905	0.5008	0.8807	0.94	1.0417	0.9191	0.9608	0.9591	0.9224
Zscore_PCC_KW_3_DT	1 [nan-nan]	0	1	1	1	1	1	1	1	1	1
Zscore_PCC_KW_3_GP	0.8315 [0.8041-0.8590]	0.014	0.8351	0.4582	0.5444	0.7696	0.8627	0.8382	0.701	0.7371	0.8125
Zscore_PCC_KW_3_NB	0.7512 [0.7181-0.7843]	0.0169	0.7236	0.9932	0.3915	0.6949	1.0956	0.6471	0.7426	0.7154	0.6779
Zscore_PCC_KW_4_SVM	0.8427 [0.8165-0.8689]	0.0134	0.8419	0.4273	0.5401	0.7659	0.826	0.8529	0.6789	0.7265	0.822
Zscore_PCC_KW_4_LDA	0.8249 [0.7970-0.8528]	0.0142	0.8218	0.5596	0.5221	0.761	1.0074	0.7574	0.7647	0.763	0.7591
Zscore_PCC_KW_4_AE	0.7873 [0.7565-0.8181]	0.0157	0.7703	0.4788	0.451	0.7255	1.0049	0.723	0.7279	0.7266	0.7244
Zscore_PCC_KW_4_RF	1 [nan-nan]	0	1	0.61	1	1	1	1	1	1	1
Zscore_PCC_KW_4_LR	0.843 [0.8169-0.8692]	0.0133	0.8443	0.4489	0.5364	0.7659	0.8701	0.8309	0.701	0.7354	0.8056
Zscore_PCC_KW_4_LRLasso	0.8432 [0.8170-0.8693]	0.0133	0.8444	0.4428	0.541	0.7672	0.8431	0.8456	0.6887	0.7309	0.8169
Zscore_PCC_KW_4_AB	0.995 [0.9925-0.9975]	0.0013	0.9952	0.5006	0.9265	0.9632	1.0098	0.9583	0.9681	0.9678	0.9587
Zscore_PCC_KW_4_DT	1 [nan-nan]	0	1	1	1	1	1	1	1	1	1
Zscore_PCC_KW_4_GP	0.8556 [0.8301-0.8811]	0.013	0.853	0.4875	0.5889	0.7941	0.951	0.8186	0.7696	0.7804	0.8093
Zscore_PCC_KW_4_NB	0.7756 [0.7440-0.8072]	0.0161	0.751	0.995	0.4267	0.7132	1.0294	0.6985	0.7279	0.7197	0.7071
Zscore_PCC_KW_5_SVM	0.8482 [0.8223-0.8740]	0.0132	0.8421	0.4543	0.5562	0.7757	0.8701	0.8407	0.7108	0.744	0.8169
Zscore_PCC_KW_5_LDA	0.8304 [0.8031-0.8578]	0.0139	0.8275	0.5775	0.5251	0.7623	1.049	0.7377	0.7868	0.7758	0.75
Zscore_PCC_KW_5_AE	0.6866 [0.6504-0.7227]	0.0184	0.6494	0.4748	0.3124	0.6556	1.0858	0.6127	0.6985	0.6702	0.6433
Zscore_PCC_KW_5_RF	1 [nan-nan]	0	1	0.64	1	1	1	1	1	1	1
Zscore_PCC_KW_5_LR	0.8478 [0.8220-0.8736]	0.0132	0.8459	0.5403	0.547	0.7733	1.0417	0.7525	0.7941	0.7852	0.7624
Zscore_PCC_KW_5_LRLasso	0.8482 [0.8225-0.8740]	0.0132	0.8447	0.4656	0.5529	0.7745	0.8824	0.8333	0.7157	0.7456	0.8111
Zscore_PCC_KW_5_AB	0.9981 [0.9968-0.9994]	0.0007	0.9981	0.5012	0.9536	0.9767	1.0172	0.9681	0.9853	0.985	0.9687
Zscore_PCC_KW_5_DT	1 [nan-nan]	0	1	1	1	1	1	1	1	1	1
Zscore_PCC_KW_5_GP	0.8668 [0.8422-0.8913]	0.0125	0.8604	0.466	0.619	0.8076	0.8897	0.8627	0.7525	0.777	0.8457
Zscore_PCC_KW_5_NB	0.7858 [0.7548-0.8169]	0.0158	0.7631	0.994	0.4575	0.7279	0.9167	0.7696	0.6863	0.7104	0.7487
Zscore_PCC_KW_6_SVM	0.8608 [0.8362-0.8853]	0.0125	0.8556	0.4417	0.5684	0.7819	0.8725	0.8456	0.7181	0.75	0.823
Zscore_PCC_KW_6_LDA	0.8421 [0.8160-0.8682]	0.0133	0.8421	0.5855	0.5256	0.7623	1.0637	0.7304	0.7941	0.7801	0.7465
Zscore_PCC_KW_6_AE	0.7962 [0.7659-0.8265]	0.0155	0.7828	0.4774	0.4814	0.7402	0.9363	0.7721	0.7083	0.7258	0.7565
Zscore_PCC_KW_6_RF	1 [nan-nan]	0	1	0.63	1	1	1	1	1	1	1
Zscore_PCC_KW_6_LR	0.8592 [0.8346-0.8838]	0.0125	0.8568	0.4584	0.5627	0.7806	0.9289	0.8162	0.7451	0.762	0.8021
Zscore_PCC_KW_6_LRLasso	0.859 [0.8344-0.8836]	0.0126	0.8559	0.4745	0.5722	0.7855	0.9387	0.8162	0.7549	0.7691	0.8042
Zscore_PCC_KW_6_AB	0.9992 [0.9984-1.0000]	0.0004	0.9992	0.4996	0.9828	0.9914	1.0025	0.9902	0.9926	0.9926	0.9902
Zscore_PCC_KW_6_DT	1 [nan-nan]	0	1	1	1	1	1	1	1	1	1
Zscore_PCC_KW_6_GP	0.8832 [0.8606-0.9059]	0.0116	0.8779	0.5025	0.64	0.8199	0.9681	0.8358	0.8039	0.81	0.8304
Zscore_PCC_KW_6_NB	0.7882 [0.7572-0.8191]	0.0158	0.765	0.9947	0.4682	0.7328	0.8971	0.7843	0.6814	0.7111	0.7596
Zscore_PCC_KW_7_SVM	0.8611 [0.8366-0.8856]	0.0125	0.8557	0.5071	0.5686	0.7843	1	0.7843	0.7843	0.7843	0.7843
Zscore_PCC_KW_7_LDA	0.8526 [0.8275-0.8778]	0.0128	0.85	0.4993	0.5497	0.7745	0.951	0.799	0.75	0.7617	0.7887
Zscore_PCC_KW_7_AE	0.7761 [0.7442-0.8079]	0.0162	0.7469	0.5702	0.4455	0.7218	0.9093	0.7672	0.6765	0.7034	0.7439
Zscore_PCC_KW_7_RF	1 [nan-nan]	0	1	0.615	1	1	1	1	1	1	1
Zscore_PCC_KW_7_LR	0.8608 [0.8363-0.8852]	0.0125	0.8568	0.5356	0.5593	0.7794	1.0392	0.7598	0.799	0.7908	0.7689
Zscore_PCC_KW_7_LRLasso	0.8598 [0.8352-0.8843]	0.0125	0.8561	0.5264	0.5588	0.7794	1.0049	0.777	0.7819	0.7808	0.778
Zscore_PCC_KW_7_AB	0.9995 [0.9990-1.0000]	0.0003	0.9995	0.5001	0.9853	0.9926	1.0098	0.9877	0.9975	0.9975	0.9879
Zscore_PCC_KW_7_DT	1 [nan-nan]	0	1	1	1	1	1	1	1	1	1
Zscore_PCC_KW_7_GP	0.8951 [0.8737-0.9165]	0.0109	0.8935	0.4521	0.6812	0.837	0.8554	0.9093	0.7647	0.7944	0.894
Zscore_PCC_KW_7_NB	0.7784 [0.7470-0.8098]	0.016	0.7593	0.9968	0.4586	0.7279	0.8922	0.7819	0.674	0.7058	0.7555
Zscore_PCC_KW_8_SVM	0.8664 [0.8423-0.8905]	0.0123	0.8595	0.4471	0.5826	0.7904	0.924	0.8284	0.7525	0.7699	0.8143
Zscore_PCC_KW_8_LDA	0.8596 [0.8352-0.8840]	0.0125	0.8574	0.5458	0.5567	0.7782	1.0368	0.7598	0.7966	0.7888	0.7683
Zscore_PCC_KW_8_AE	0.7373 [0.7032-0.7713]	0.0174	0.7079	0.4981	0.4027	0.6985	0.8333	0.7819	0.6152	0.6702	0.7382
Zscore_PCC_KW_8_RF	1 [nan-nan]	0	1	0.62	1	1	1	1	1	1	1
Zscore_PCC_KW_8_LR	0.8651 [0.8412-0.8891]	0.0122	0.8618	0.4378	0.5685	0.7831	0.9093	0.8284	0.7377	0.7596	0.8113
Zscore_PCC_KW_8_LRLasso	0.8648 [0.8407-0.8888]	0.0123	0.8613	0.5268	0.5687	0.7843	1.0098	0.7794	0.7892	0.7871	0.7816
Zscore_PCC_KW_8_AB	0.9994 [0.9988-1.0000]	0.0003	0.9994	0.4997	0.9779	0.989	0.9975	0.9902	0.9877	0.9878	0.9902
Zscore_PCC_KW_8_DT	1 [nan-nan]	0	1	1	1	1	1	1	1	1	1
Zscore_PCC_KW_8_GP	0.9037 [0.8831-0.9242]	0.0105	0.9008	0.4722	0.6896	0.8431	0.902	0.8922	0.7941	0.8125	0.8804
Zscore_PCC_KW_8_NB	0.7894 [0.7587-0.8201]	0.0157	0.7694	0.9989	0.4683	0.7341	1.0221	0.723	0.7451	0.7393	0.729
Zscore_PCC_KW_9_SVM	0.874 [0.8505-0.8974]	0.012	0.8653	0.464	0.6055	0.8027	0.9828	0.8113	0.7941	0.7976	0.808
Zscore_PCC_KW_9_LDA	0.8743 [0.8510-0.8976]	0.0119	0.8674	0.5155	0.6086	0.8039	1.049	0.7794	0.8284	0.8196	0.7897
Zscore_PCC_KW_9_AE	0.7944 [0.7638-0.8250]	0.0156	0.7787	0.4989	0.4792	0.739	1.0711	0.7034	0.7745	0.7573	0.7231
Zscore_PCC_KW_9_RF	1 [nan-nan]	0	1	0.63	1	1	1	1	1	1	1
Zscore_PCC_KW_9_LR	0.8727 [0.8494-0.8960]	0.0119	0.8668	0.3857	0.6009	0.7953	0.8162	0.8873	0.7034	0.7495	0.8619
Zscore_PCC_KW_9_LRLasso	0.8735 [0.8503-0.8968]	0.0119	0.8683	0.5207	0.5983	0.799	1.0294	0.7843	0.8137	0.8081	0.7905
Zscore_PCC_KW_9_AB	0.9998 [0.9996-1.0000]	0.0001	0.9999	0.5002	0.9927	0.9963	1.0025	0.9951	0.9975	0.9975	0.9951
Zscore_PCC_KW_9_DT	1 [nan-nan]	0	1	1	1	1	1	1	1	1	1
Zscore_PCC_KW_9_GP	0.919 [0.9001-0.9379]	0.0096	0.9136	0.471	0.7441	0.8701	0.8971	0.9216	0.8186	0.8356	0.9126
Zscore_PCC_KW_9_NB	0.7903 [0.7596-0.8210]	0.0157	0.7735	0.9989	0.4681	0.7341	0.9926	0.7377	0.7304	0.7324	0.7358

Zscore_PCC_KW_10_SVM	0.8761 [0.8529-0.8993]	0.0118	0.8681	0.5193	0.6136	0.8064	1.0539	0.7794	0.8333	0.8238	0.7907
Zscore_PCC_KW_10_LDA	0.8765 [0.8535-0.8995]	0.0118	0.8684	0.4797	0.6128	0.8064	0.9902	0.8113	0.8015	0.8034	0.8094
Zscore_PCC_KW_10_AE	0.795 [0.7649-0.8251]	0.0154	0.8022	0.5002	0.4581	0.7279	0.902	0.777	0.6789	0.7076	0.7527
Zscore_PCC_KW_10_RF	1 [nan-nan]	0	1	0.625	1	1	1	1	1	1	1
Zscore_PCC_KW_10_LR	0.8738 [0.8506-0.8969]	0.0118	0.8694	0.3916	0.6016	0.7966	0.8333	0.8799	0.7132	0.7542	0.8559
Zscore_PCC_KW_10_LRLasso	0.8749 [0.8519-0.8979]	0.0117	0.8713	0.539	0.5947	0.7966	1.0735	0.7598	0.8333	0.8201	0.7763
Zscore_PCC_KW_10_AB	0.9998 [0.9996-1.0000]	0.0001	0.9999	0.4997	0.9902	0.9951	1	0.9951	0.9951	0.9951	0.9951
Zscore_PCC_KW_10_DT	1 [nan-nan]	0	1	1	1	1	1	1	1	1	1
Zscore_PCC_KW_10_GP	0.9288 [0.9113-0.9463]	0.0089	0.924	0.4657	0.7575	0.8762	0.8848	0.9338	0.8186	0.8374	0.9252
Zscore_PCC_KW_10_NB	0.7921 [0.7615-0.8228]	0.0156	0.7748	0.999	0.4706	0.7353	0.9853	0.7426	0.7279	0.7319	0.7388
Zscore_PCC_KW_11_SVM	0.8762 [0.8530-0.8994]	0.0118	0.868	0.5214	0.6162	0.8076	1.0564	0.7794	0.8358	0.826	0.7912
Zscore_PCC_KW_11_LDA	0.8782 [0.8554-0.9010]	0.0116	0.8716	0.5286	0.6168	0.8076	1.0711	0.7721	0.8431	0.8311	0.7872
Zscore_PCC_KW_11_AE	0.6005 [0.5616-0.6394]	0.0198	0.5694	0.4924	0.278	0.6054	0.348	0.9314	0.2794	0.5638	0.8028
Zscore_PCC_KW_11_RF	1 [nan-nan]	0	1	0.64	1	1	1	1	1	1	1
Zscore_PCC_KW_11_LR	0.8732 [0.8500-0.8964]	0.0118	0.8692	0.3941	0.6016	0.7966	0.8333	0.8799	0.7132	0.7542	0.8559
Zscore_PCC_KW_11_LRLasso	0.8749 [0.8518-0.8979]	0.0118	0.8713	0.539	0.5947	0.7966	1.0735	0.7598	0.8333	0.8201	0.7763
Zscore_PCC_KW_11_AB	1 [1.0000-1.0000]	0	1	0.5006	0.9976	0.9988	0.9975	1	0.9975	0.9976	1
Zscore_PCC_KW_11_DT	1 [nan-nan]	0	1	1	1	1	1	1	1	1	1
Zscore_PCC_KW_11_GP	0.9334 [0.9166-0.9502]	0.0086	0.9294	0.4817	0.7642	0.8811	0.9289	0.9167	0.8456	0.8558	0.9103
Zscore_PCC_KW_11_NB	0.7976 [0.7673-0.8279]	0.0155	0.789	0.9995	0.4839	0.7414	0.9338	0.7745	0.7083	0.7264	0.7585
Zscore_PCC_KW_12_SVM	0.88 [0.8573-0.9027]	0.0116	0.8724	0.5374	0.6097	0.8039	1.0784	0.7647	0.8431	0.8298	0.7818
Zscore_PCC_KW_12_LDA	0.881 [0.8586-0.9034]	0.0114	0.8763	0.5483	0.6154	0.8064	1.0931	0.7598	0.8529	0.8378	0.7803
Zscore_PCC_KW_12_AE	0.746 [0.7122-0.7798]	0.0172	0.7179	0.5071	0.4288	0.712	0.8505	0.7868	0.6373	0.6844	0.7493
Zscore_PCC_KW_12_RF	1 [nan-nan]	0	1	0.635	1	1	1	1	1	1	1
Zscore_PCC_KW_12_LR	0.8754 [0.8525-0.8983]	0.0117	0.8725	0.4646	0.5884	0.7941	0.9755	0.8064	0.7819	0.7871	0.8015
Zscore_PCC_KW_12_LRLasso	0.8769 [0.8541-0.8996]	0.0116	0.8742	0.5349	0.5967	0.7978	1.0613	0.7672	0.8284	0.8172	0.7806
Zscore_PCC_KW_12_AB	1 [0.9999-1.0000]	0	1	0.503	0.9951	0.9975	1.0049	0.9951	1	1	0.9951
Zscore_PCC_KW_12_DT	1 [nan-nan]	0	1	1	1	1	1	1	1	1	1
Zscore_PCC_KW_12_GP	0.9431 [0.9278-0.9584]	0.0078	0.9394	0.4777	0.7815	0.8897	0.9265	0.9265	0.8529	0.863	0.9206
Zscore_PCC_KW_12_NB	0.7944 [0.7639-0.8249]	0.0156	0.7839	0.9998	0.4707	0.7353	1.0196	0.7255	0.7451	0.74	0.7308
Zscore_PCC_KW_13_SVM	0.8815 [0.8590-0.9041]	0.0115	0.8748	0.53	0.6315	0.815	1.0711	0.7794	0.8505	0.8391	0.7941
Zscore_PCC_KW_13_LDA	0.8838 [0.8617-0.9060]	0.0113	0.8784	0.4885	0.6252	0.8125	1.0221	0.8015	0.8235	0.8195	0.8058
Zscore_PCC_KW_13_AE	0.7975 [0.7669-0.8282]	0.0157	0.7688	0.494	0.4958	0.7475	0.9461	0.7745	0.7206	0.7349	0.7617
Zscore_PCC_KW_13_RF	1 [nan-nan]	0	1	0.635	1	1	1	1	1	1	1
Zscore_PCC_KW_13_LR	0.8771 [0.8543-0.8999]	0.0116	0.8724	0.4157	0.6012	0.799	0.8971	0.8505	0.7475	0.7711	0.8333
Zscore_PCC_KW_13_LRLasso	0.8791 [0.8566-0.9016]	0.0115	0.8744	0.5333	0.5969	0.7978	1.0662	0.7647	0.8309	0.8189	0.7793
Zscore_PCC_KW_13_AB	1 [nan-nan]	0	1	0.4993	1	1	1	1	1	1	1
Zscore_PCC_KW_13_DT	1 [nan-nan]	0	1	1	1	1	1	1	1	1	1
Zscore_PCC_KW_13_GP	0.9455 [0.9306-0.9605]	0.0076	0.942	0.4675	0.7972	0.8971	0.9118	0.9412	0.8529	0.8649	0.9355
Zscore_PCC_KW_13_NB	0.7934 [0.7629-0.8240]	0.0156	0.7825	0.9999	0.4683	0.7341	1.0221	0.723	0.7451	0.7393	0.729
Zscore_PCC_KW_14_SVM	0.8827 [0.8601-0.9052]	0.0115	0.8756	0.5123	0.6331	0.8162	1.049	0.7917	0.8407	0.8325	0.8014
Zscore_PCC_KW_14_LDA	0.8854 [0.8634-0.9074]	0.0112	0.8808	0.451	0.635	0.8174	0.9779	0.8284	0.8064	0.8106	0.8246
Zscore_PCC_KW_14_AE	0.8073 [0.7783-0.8363]	0.0148	0.8109	0.6655	0.4785	0.7353	1.1814	0.6446	0.826	0.7874	0.6992
Zscore_PCC_KW_14_RF	1 [nan-nan]	0	1	0.645	1	1	1	1	1	1	1
Zscore_PCC_KW_14_LR	0.8779 [0.8553-0.9006]	0.0116	0.8738	0.4291	0.605	0.8015	0.9167	0.8431	0.7598	0.7783	0.8289
Zscore_PCC_KW_14_LRLasso	0.8801 [0.8576-0.9025]	0.0115	0.8755	0.529	0.6035	0.8015	1.0441	0.7794	0.8235	0.8154	0.7887
Zscore_PCC_KW_14_AB	1 [1.0000-1.0000]	0	1	0.5013	0.9976	0.9988	1.0025	0.9975	1	1	0.9976
Zscore_PCC_KW_14_DT	1 [nan-nan]	0	1	1	1	1	1	1	1	1	1
Zscore_PCC_KW_14_GP	0.9515 [0.9374-0.9657]	0.0072	0.9488	0.4721	0.8038	0.9007	0.924	0.9387	0.8627	0.8724	0.9337
Zscore_PCC_KW_14_NB	0.792 [0.7614-0.8226]	0.0156	0.7807	0.9999	0.4583	0.7292	0.9975	0.7304	0.7279	0.7286	0.7297
Zscore_PCC_KW_15_SVM	0.8866 [0.8645-0.9088]	0.0113	0.8807	0.5052	0.6453	0.8223	1.0466	0.799	0.8456	0.838	0.808
Zscore_PCC_KW_15_LDA	0.8883 [0.8666-0.9100]	0.0111	0.8844	0.4679	0.6448	0.8223	0.9779	0.8333	0.8113	0.8153	0.8296
Zscore_PCC_KW_15_AE	0.7773 [0.7459-0.8087]	0.016	0.7697	0.5496	0.444	0.7218	0.9583	0.7426	0.701	0.7129	0.7315
Zscore_PCC_KW_15_RF	1 [nan-nan]	0	1	0.645	1	1	1	1	1	1	1
Zscore_PCC_KW_15_LR	0.8798 [0.8573-0.9024]	0.0115	0.8751	0.4191	0.6213	0.8088	0.8922	0.8627	0.7549	0.7788	0.8462
Zscore_PCC_KW_15_LRLasso	0.8834 [0.8612-0.9055]	0.0113	0.8789	0.5014	0.6128	0.8064	0.9951	0.8088	0.8039	0.8049	0.8079
Zscore_PCC_KW_15_AB	1 [nan-nan]	0	1	0.5026	1	1	1	1	1	1	1
Zscore_PCC_KW_15_DT	1 [nan-nan]	0	1	1	1	1	1	1	1	1	1
Zscore_PCC_KW_15_GP	0.9569 [0.9435-0.9703]	0.0068	0.9537	0.4724	0.8264	0.9118	0.9167	0.9534	0.8701	0.8801	0.9492
Zscore_PCC_KW_15_NB	0.7958 [0.7654-0.8262]	0.0155	0.7878	1	0.4731	0.7365	0.9828	0.7451	0.7279	0.7325	0.7406
Zscore_PCC_KW_16_SVM	0.8854 [0.8632-0.9077]	0.0114	0.8795	0.4938	0.6424	0.8211	1.0294	0.8064	0.8358	0.8308	0.8119
Zscore_PCC_KW_16_LDA	0.8903 [0.8688-0.9117]	0.0109	0.8862	0.4816	0.6544	0.8272	0.9975	0.8284	0.826	0.8264	0.828
Zscore_PCC_KW_16_AE	0.7856 [0.7547-0.8165]	0.0158	0.7792	0.4897	0.4657	0.7328	1	0.7328	0.7328	0.7328	0.7328
Zscore_PCC_KW_16_RF	1 [nan-nan]	0	1	0.65	1	1	1	1	1	1	1
Zscore_PCC_KW_16_LR	0.8815 [0.8592-0.9038]	0.0114	0.8766	0.4294	0.6247	0.8113	0.9167	0.8529	0.7696	0.7873	0.8396
Zscore_PCC_KW_16_LRLasso	0.8843 [0.8623-0.9064]	0.0113	0.8791	0.5255	0.6156	0.8076	1.0368	0.7892	0.826	0.8193	0.7967
Zscore_PCC_KW_16_AB	1 [1.0000-1.0000]	0	1	0.5021	0.9976	0.9988	1.0025	0.9975	1	1	0.9976
Zscore_PCC_KW_16_DT	1 [nan-nan]	0	1	1	1	1	1	1	1	1	1
Zscore_PCC_KW_16_GP	0.9635 [0.9513-0.9756]	0.0062	0.9604	0.5101	0.8407	0.9203	1.0025	0.9191	0.9216	0.9214	0.9193
Zscore_PCC_KW_16_NB	0.7962 [0.7659-0.8266]	0.0155	0.7942	1	0.4755	0.7377	1	0.7377	0.7377	0.7377	0.7377
Zscore_PCC_KW_17_SVM	0.889 [0.8670-0.9110]	0.0112	0.8807	0.4948	0.6373	0.8186	1.0147	0.8113	0.826	0.8234	0.814
Zscore_PCC_KW_17_LDA	0.8917 [0.8704-0.9129]	0.0108	0.8862	0.4778	0.6373	0.8186	0.9853	0.826	0.8113	0.814	0.8234
Zscore_PCC_KW_17_AE	0.8163 [0.7876-0.8450]	0.0147	0.8091	0.5566	0.5148	0.7574	0.9804	0.7672	0.7475	0.7524	0.7625

Zscore_PCC_KW_17_RF	1 [nan-nan]	0	1	0.635	1	1	1	1	1	1	1
Zscore_PCC_KW_17_LR	0.8836 [0.8615-0.9057]	0.0113	0.8779	0.4136	0.6216	0.8088	0.8873	0.8652	0.7525	0.7775	0.8481
Zscore_PCC_KW_17_LRLasso	0.8853 [0.8633-0.9072]	0.0112	0.8789	0.5256	0.6179	0.8088	1.0294	0.7941	0.8235	0.8182	0.8
Zscore_PCC_KW_17_AB	1 [nan-nan]	0	1	0.5037	1	1	1	1	1	1	1
Zscore_PCC_KW_17_DT	1 [nan-nan]	0	1	1	1	1	1	1	1	1	1
Zscore_PCC_KW_17_GP	0.9697 [0.9586-0.9808]	0.0057	0.9672	0.5091	0.8603	0.9301	1.0025	0.9289	0.9314	0.9312	0.9291
Zscore_PCC_KW_17_NB	0.7939 [0.7633-0.8245]	0.0156	0.7899	1	0.48	0.7365	1.1691	0.652	0.8211	0.7847	0.7023
Zscore_PCC_KW_18_SVM	0.892 [0.8703-0.9136]	0.011	0.8845	0.4343	0.6384	0.8174	0.8946	0.8701	0.7647	0.7871	0.8548
Zscore_PCC_KW_18_LDA	0.8969 [0.8763-0.9175]	0.0105	0.8918	0.4236	0.6338	0.8162	0.9314	0.8505	0.7819	0.7959	0.8395
Zscore_PCC_KW_18_AE	0.7607 [0.7280-0.7934]	0.0167	0.7251	0.5049	0.4167	0.7083	1.0196	0.6985	0.7181	0.7125	0.7043
Zscore_PCC_KW_18_RF	1 [nan-nan]	0	1	0.655	1	1	1	1	1	1	1
Zscore_PCC_KW_18_LR	0.8849 [0.8629-0.9070]	0.0113	0.877	0.4403	0.6245	0.8113	0.9216	0.8505	0.7721	0.7886	0.8378
Zscore_PCC_KW_18_LRLasso	0.8882 [0.8665-0.9099]	0.0111	0.8806	0.446	0.6219	0.81	0.924	0.848	0.7721	0.7882	0.8355
Zscore_PCC_KW_18_AB	1 [nan-nan]	0	1	0.5013	1	1	1	1	1	1	1
Zscore_PCC_KW_18_DT	1 [nan-nan]	0	1	1	1	1	1	1	1	1	1
Zscore_PCC_KW_18_GP	0.9748 [0.9648-0.9848]	0.0051	0.9729	0.5019	0.8874	0.9436	0.9853	0.951	0.9363	0.9372	0.9502
Zscore_PCC_KW_18_NB	0.7939 [0.7633-0.8245]	0.0156	0.7906	1	0.4977	0.7439	1.1985	0.6446	0.8431	0.8043	0.7035
Zscore_PCC_KW_19_SVM	0.8996 [0.8789-0.9202]	0.0105	0.8921	0.4089	0.6535	0.8223	0.8358	0.9044	0.7402	0.7768	0.8856
Zscore_PCC_KW_19_LDA	0.9012 [0.8812-0.9212]	0.0102	0.8984	0.5022	0.6399	0.8199	1.027	0.8064	0.8333	0.8287	0.8115
Zscore_PCC_KW_19_AE	0.8387 [0.8121-0.8654]	0.0136	0.8479	0.4909	0.5392	0.7659	1.1642	0.6838	0.848	0.8182	0.7284
Zscore_PCC_KW_19_RF	1 [nan-nan]	0	1	0.645	1	1	1	1	1	1	1
Zscore_PCC_KW_19_LR	0.8911 [0.8698-0.9125]	0.0109	0.8841	0.429	0.6328	0.815	0.9044	0.8627	0.7672	0.7875	0.8482
Zscore_PCC_KW_19_LRLasso	0.8937 [0.8727-0.9148]	0.0107	0.8877	0.4747	0.6277	0.8137	0.9706	0.8284	0.799	0.8048	0.8232
Zscore_PCC_KW_19_AB	1 [nan-nan]	0	1	0.5001	1	1	1	1	1	1	1
Zscore_PCC_KW_19_DT	1 [nan-nan]	0	1	1	1	1	1	1	1	1	1
Zscore_PCC_KW_19_GP	0.9811 [0.9726-0.9896]	0.0043	0.9805	0.4981	0.9119	0.9559	0.9853	0.9632	0.9485	0.9493	0.9627
Zscore_PCC_KW_19_NB	0.7914 [0.7606-0.8222]	0.0157	0.7865	1	0.4788	0.7365	1.1544	0.6593	0.8137	0.7797	0.7049
Zscore_PCC_KW_20_SVM	0.903 [0.8828-0.9232]	0.0103	0.8962	0.4646	0.6529	0.826	0.9461	0.8529	0.799	0.8093	0.8446
Zscore_PCC_KW_20_LDA	0.9029 [0.8831-0.9227]	0.0101	0.9002	0.4259	0.6533	0.826	0.9363	0.8578	0.7941	0.8065	0.8482
Zscore_PCC_KW_20_AE	0.7399 [0.7061-0.7737]	0.0172	0.7009	0.5194	0.3806	0.6887	0.8725	0.7525	0.625	0.6674	0.7163
Zscore_PCC_KW_20_RF	1 [nan-nan]	0	1	0.63	1	1	1	1	1	1	1
Zscore_PCC_KW_20_LR	0.8929 [0.8718-0.9141]	0.0108	0.8863	0.4004	0.6308	0.8125	0.8652	0.8799	0.7451	0.7754	0.8612
Zscore_PCC_KW_20_LRLasso	0.8971 [0.8765-0.9178]	0.0105	0.8914	0.3892	0.6552	0.8223	0.8211	0.9118	0.7328	0.7734	0.8925
Zscore_PCC_KW_20_AB	1 [nan-nan]	0	1	0.5036	1	1	1	1	1	1	1
Zscore_PCC_KW_20_DT	1 [nan-nan]	0	1	1	1	1	1	1	1	1	1
Zscore_PCC_KW_20_GP	0.9848 [0.9773-0.9923]	0.0038	0.984	0.4995	0.9167	0.9583	0.9951	0.9608	0.9559	0.9561	0.9606
Zscore_PCC_KW_20_NB	0.7856 [0.7544-0.8168]	0.0159	0.7781	1	0.4539	0.7267	1.0466	0.7034	0.75	0.7378	0.7166
Zscore_PCC_RFE_1_SVM	0.7289 [0.6947-0.7632]	0.0175	0.6982	0.4606	0.3843	0.6863	0.7549	0.8088	0.5637	0.6496	0.7468
Zscore_PCC_RFE_1_LDA	0.7276 [0.6932-0.7619]	0.0175	0.6951	0.498	0.367	0.6814	0.848	0.7574	0.6054	0.6574	0.7139
Zscore_PCC_RFE_1_AE	0.7126 [0.6774-0.7477]	0.0179	0.6829	0.5252	0.3414	0.6703	0.9338	0.7034	0.6373	0.6598	0.6824
Zscore_PCC_RFE_1_RF	1 [nan-nan]	0	1	0.58	1	1	1	1	1	1	1
Zscore_PCC_RFE_1_LR	0.7288 [0.6945-0.7631]	0.0175	0.6981	0.4528	0.3756	0.6814	0.7402	0.8113	0.5515	0.644	0.745
Zscore_PCC_RFE_1_LRLasso	0.7289 [0.6946-0.7631]	0.0175	0.6982	0.4632	0.3802	0.6838	0.7451	0.8113	0.5564	0.6465	0.7467
Zscore_PCC_RFE_1_AB	0.9 [0.8804-0.9197]	0.01	0.9056	0.4997	0.616	0.8064	0.8971	0.8578	0.7549	0.7778	0.8415
Zscore_PCC_RFE_1_DT	1 [nan-nan]	0	1	1	1	1	1	1	1	1	1
Zscore_PCC_RFE_1_GP	0.7299 [0.6956-0.7642]	0.0175	0.7107	0.5244	0.3689	0.6838	0.9167	0.7255	0.6422	0.6697	0.7005
Zscore_PCC_RFE_1_NB	0.703 [0.6675-0.7386]	0.0182	0.6649	0.5156	0.3355	0.6618	0.7353	0.7941	0.5294	0.6279	0.72
Zscore_PCC_RFE_2_SVM	0.8078 [0.7790-0.8366]	0.0147	0.817	0.5793	0.4648	0.7316	1.0809	0.6912	0.7721	0.752	0.7143
Zscore_PCC_RFE_2_LDA	0.8049 [0.7758-0.8339]	0.0148	0.8129	0.5828	0.4543	0.7267	1.0613	0.6961	0.7574	0.7415	0.7136
Zscore_PCC_RFE_2_AE	0.8 [0.7705-0.8294]	0.015	0.8062	0.4582	0.4365	0.7169	0.8897	0.7721	0.6618	0.6954	0.7438
Zscore_PCC_RFE_2_RF	1 [nan-nan]	0	1	0.585	1	1	1	1	1	1	1
Zscore_PCC_RFE_2_LR	0.8078 [0.7790-0.8366]	0.0147	0.8174	0.5731	0.4666	0.7328	1.0637	0.701	0.7647	0.7487	0.7189
Zscore_PCC_RFE_2_LRLasso	0.8079 [0.7791-0.8367]	0.0147	0.8177	0.5773	0.4668	0.7328	1.0686	0.6985	0.7672	0.75	0.7179
Zscore_PCC_RFE_2_AB	0.9605 [0.9499-0.9712]	0.0054	0.9629	0.4985	0.7549	0.8762	0.9191	0.9167	0.8358	0.8481	0.9093
Zscore_PCC_RFE_2_DT	1 [nan-nan]	0	1	1	1	1	1	1	1	1	1
Zscore_PCC_RFE_2_GP	0.8154 [0.7869-0.8438]	0.0145	0.8179	0.5492	0.4797	0.739	1.0858	0.6961	0.7819	0.7614	0.7201
Zscore_PCC_RFE_2_NB	0.7768 [0.7455-0.8081]	0.016	0.7581	0.6987	0.4099	0.7047	1.0515	0.6789	0.7304	0.7158	0.6946
Zscore_PCC_RFE_3_SVM	0.8419 [0.8159-0.8679]	0.0133	0.8546	0.6317	0.5305	0.7586	1.223	0.6471	0.8701	0.8328	0.7114
Zscore_PCC_RFE_3_LDA	0.8388 [0.8128-0.8648]	0.0133	0.8578	0.6792	0.5142	0.7488	1.2525	0.6225	0.875	0.8328	0.6986
Zscore_PCC_RFE_3_AE	0.7826 [0.7513-0.8138]	0.0159	0.823	0.4924	0.4705	0.7267	1.2672	0.5931	0.8603	0.8094	0.6789
Zscore_PCC_RFE_3_RF	1 [nan-nan]	0	1	0.58	1	1	1	1	1	1	1
Zscore_PCC_RFE_3_LR	0.8422 [0.8163-0.8681]	0.0132	0.8549	0.6399	0.526	0.7574	1.2059	0.6544	0.8603	0.8241	0.7134
Zscore_PCC_RFE_3_LRLasso	0.8424 [0.8165-0.8682]	0.0132	0.8555	0.6413	0.5237	0.7561	1.2083	0.652	0.8603	0.8235	0.712
Zscore_PCC_RFE_3_AB	0.9856 [0.9803-0.9908]	0.0027	0.986	0.5006	0.8538	0.9265	1.0441	0.9044	0.9485	0.9462	0.9085
Zscore_PCC_RFE_3_DT	1 [nan-nan]	0	1	1	1	1	1	1	1	1	1
Zscore_PCC_RFE_3_GP	0.857 [0.8321-0.8819]	0.0127	0.8693	0.5394	0.5559	0.7745	1.1569	0.6961	0.8529	0.8256	0.7373
Zscore_PCC_RFE_3_NB	0.8118 [0.7835-0.8400]	0.0144	0.8296	0.8062	0.4704	0.7255	1.2843	0.5833	0.8676	0.8151	0.6756
Zscore_PCC_RFE_4_SVM	0.8644 [0.8402-0.8886]	0.0124	0.8571	0.4497	0.5711	0.7843	0.9069	0.8309	0.7377	0.7601	0.8135
Zscore_PCC_RFE_4_LDA	0.8596 [0.8354-0.8839]	0.0124	0.8625	0.4627	0.5458	0.7721	0.9216	0.8113	0.7328	0.7523	0.7952
Zscore_PCC_RFE_4_AE	0.7989 [0.7687-0.8291]	0.0154	0.7549	0.4745	0.4848	0.7414	1.0907	0.6961	0.7868	0.7655	0.7213
Zscore_PCC_RFE_4_RF	1 [nan-nan]	0	1	0.625	1	1	1	1	1	1	1
Zscore_PCC_RFE_4_LR	0.865 [0.8410-0.8891]	0.0123	0.8603	0.4131	0.5741	0.7843	0.8627	0.8529	0.7157	0.75	0.8295
Zscore_PCC_RFE_4_LRLasso	0.8649 [0.8409-0.8889]	0.0123	0.8614	0.5201	0.5639	0.7819	1.0245	0.7696	0.7941	0.7889	0.7751

Zscore_PCC_RFE_4_AB	0.9953 [0.9929-0.9978]	0.0012	0.9955	0.5021	0.9227	0.9608	1.049	0.9363	0.9853	0.9845	0.9393
Zscore_PCC_RFE_4_DT	1 [nan-nan]	0	1	1	1	1	1	1	1	1	1
Zscore_PCC_RFE_4_GP	0.8915 [0.8702-0.9129]	0.0109	0.887	0.5004	0.625	0.8125	1.0074	0.8088	0.8162	0.8148	0.8102
Zscore_PCC_RFE_4_NB	0.8293 [0.8023-0.8564]	0.0138	0.8352	0.6969	0.4976	0.7488	1.0123	0.7426	0.7549	0.7519	0.7458
Zscore_PCC_RFE_5_SVM	0.8675 [0.8436-0.8914]	0.0122	0.8643	0.4894	0.5784	0.7892	0.9951	0.7917	0.7868	0.7878	0.7906
Zscore_PCC_RFE_5_LDA	0.8612 [0.8371-0.8852]	0.0123	0.864	0.4699	0.5498	0.7745	0.9461	0.8015	0.7475	0.7605	0.7902
Zscore_PCC_RFE_5_AE	0.794 [0.7638-0.8242]	0.0154	0.7956	0.495	0.4634	0.7255	1.2304	0.6103	0.8407	0.793	0.6833
Zscore_PCC_RFE_5_RF	1 [nan-nan]	0	1	0.615	1	1	1	1	1	1	1
Zscore_PCC_RFE_5_LR	0.8682 [0.8445-0.8919]	0.0121	0.8647	0.4974	0.5858	0.7929	1.0025	0.7917	0.7941	0.7936	0.7922
Zscore_PCC_RFE_5_LRLasso	0.8673 [0.8436-0.8910]	0.0121	0.8649	0.5235	0.5737	0.7868	1.0245	0.7745	0.799	0.794	0.7799
Zscore_PCC_RFE_5_AB	0.9981 [0.9969-0.9994]	0.0006	0.9982	0.4992	0.9562	0.9779	0.9755	0.9902	0.9657	0.9665	0.9899
Zscore_PCC_RFE_5_DT	1 [nan-nan]	0	1	1	1	1	1	1	1	1	1
Zscore_PCC_RFE_5_GP	0.8992 [0.8783-0.9201]	0.0107	0.8914	0.4936	0.6619	0.8309	0.9804	0.8407	0.8211	0.8245	0.8375
Zscore_PCC_RFE_5_NB	0.8238 [0.7961-0.8515]	0.0141	0.8272	0.7853	0.5181	0.7586	1.0613	0.7279	0.7892	0.7755	0.7436
Zscore_PCC_RFE_6_SVM	0.8728 [0.8494-0.8961]	0.0119	0.8667	0.4935	0.5932	0.7966	1.0098	0.7917	0.8015	0.7995	0.7937
Zscore_PCC_RFE_6_LDA	0.8677 [0.8442-0.8912]	0.012	0.8651	0.4836	0.5613	0.7806	0.9975	0.7819	0.7794	0.78	0.7813
Zscore_PCC_RFE_6_AE	0.8083 [0.7789-0.8376]	0.015	0.8166	0.4817	0.505	0.7512	1.1005	0.701	0.8015	0.7793	0.7283
Zscore_PCC_RFE_6_RF	1 [nan-nan]	0	1	0.66	1	1	1	1	1	1	1
Zscore_PCC_RFE_6_LR	0.8738 [0.8506-0.8970]	0.0118	0.8676	0.503	0.5957	0.7978	1.0221	0.7868	0.8088	0.8045	0.7914
Zscore_PCC_RFE_6_LRLasso	0.8728 [0.8496-0.8960]	0.0118	0.868	0.5212	0.5837	0.7917	1.0343	0.7745	0.8088	0.802	0.782
Zscore_PCC_RFE_6_AB	0.999 [0.9983-0.9998]	0.0004	0.9991	0.4999	0.9731	0.9865	0.9926	0.9902	0.9828	0.983	0.9901
Zscore_PCC_RFE_6_DT	1 [nan-nan]	0	1	1	1	1	1	1	1	1	1
Zscore_PCC_RFE_6_GP	0.9104 [0.8908-0.9299]	0.01	0.9007	0.4763	0.6959	0.8468	0.9191	0.8873	0.8064	0.8209	0.8773
Zscore_PCC_RFE_6_NB	0.8296 [0.8024-0.8568]	0.0139	0.8374	0.7773	0.5147	0.7574	1.0098	0.7525	0.7623	0.7599	0.7549
Zscore_PCC_RFE_7_SVM	0.8765 [0.8535-0.8994]	0.0117	0.8705	0.5289	0.5937	0.7966	1.0441	0.7745	0.8186	0.8103	0.784
Zscore_PCC_RFE_7_LDA	0.8737 [0.8507-0.8966]	0.0117	0.8712	0.5497	0.5877	0.7929	1.0809	0.7525	0.8333	0.8187	0.771
Zscore_PCC_RFE_7_AE	0.8003 [0.7703-0.8303]	0.0153	0.8004	0.5466	0.4969	0.7463	0.8701	0.8113	0.6814	0.718	0.7831
Zscore_PCC_RFE_7_RF	1 [nan-nan]	0	1	0.655	1	1	1	1	1	1	1
Zscore_PCC_RFE_7_LR	0.8783 [0.8557-0.9009]	0.0115	0.8736	0.5349	0.6016	0.8002	1.0613	0.7696	0.8309	0.8198	0.7829
Zscore_PCC_RFE_7_LRLasso	0.8748 [0.8518-0.8978]	0.0117	0.8699	0.5104	0.5933	0.7966	1.0196	0.7868	0.8064	0.8025	0.7909
Zscore_PCC_RFE_7_AB	0.9995 [0.9990-1.0000]	0.0003	0.9995	0.5009	0.9878	0.9939	1.0074	0.9902	0.9975	0.9975	0.9903
Zscore_PCC_RFE_7_DT	1 [nan-nan]	0	1	1	1	1	1	1	1	1	1
Zscore_PCC_RFE_7_GP	0.9155 [0.8965-0.9346]	0.0097	0.9052	0.4823	0.7083	0.8529	0.9167	0.8946	0.8113	0.8258	0.885
Zscore_PCC_RFE_7_NB	0.8245 [0.7969-0.8522]	0.0141	0.831	0.8333	0.5099	0.7549	0.9804	0.7647	0.7451	0.75	0.76
Zscore_PCC_RFE_8_SVM	0.8821 [0.8598-0.9045]	0.0114	0.8795	0.4643	0.6211	0.81	0.9436	0.8382	0.7819	0.7935	0.8286
Zscore_PCC_RFE_8_LDA	0.8804 [0.8581-0.9027]	0.0114	0.8792	0.5015	0.5959	0.7978	1.0319	0.7819	0.8137	0.8076	0.7886
Zscore_PCC_RFE_8_AE	0.7023 [0.6666-0.7380]	0.0182	0.6843	0.4981	0.3649	0.6789	0.8039	0.777	0.5809	0.6496	0.7226
Zscore_PCC_RFE_8_RF	1 [nan-nan]	0	1	0.66	1	1	1	1	1	1	1
Zscore_PCC_RFE_8_LR	0.8816 [0.8593-0.9039]	0.0114	0.8775	0.5082	0.6128	0.8064	1.0147	0.799	0.8137	0.8109	0.8019
Zscore_PCC_RFE_8_LRLasso	0.8785 [0.8558-0.9011]	0.0116	0.8735	0.4943	0.6006	0.8002	0.9828	0.8088	0.7917	0.7952	0.8055
Zscore_PCC_RFE_8_AB	0.9998 [0.9996-1.0000]	0.0001	0.9998	0.5011	0.9878	0.9939	1.0123	0.9877	1	1	0.9879
Zscore_PCC_RFE_8_DT	1 [nan-nan]	0	1	1	1	1	1	1	1	1	1
Zscore_PCC_RFE_8_GP	0.9277 [0.9102-0.9453]	0.009	0.9161	0.4862	0.7353	0.8664	0.9191	0.9069	0.826	0.839	0.8987
Zscore_PCC_RFE_8_NB	0.8233 [0.7955-0.8511]	0.0142	0.8332	0.8496	0.52	0.7598	0.9608	0.7794	0.7402	0.75	0.7704
Zscore_PCC_RFE_9_SVM	0.8831 [0.8609-0.9053]	0.0113	0.8804	0.4745	0.6209	0.81	0.9485	0.8358	0.7843	0.7949	0.8269
Zscore_PCC_RFE_9_LDA	0.8814 [0.8591-0.9037]	0.0114	0.8777	0.4515	0.6106	0.8051	0.9681	0.8211	0.7892	0.7957	0.8152
Zscore_PCC_RFE_9_AE	0.7209 [0.6861-0.7558]	0.0178	0.7038	0.4774	0.3554	0.6777	0.9926	0.6814	0.674	0.6764	0.679
Zscore_PCC_RFE_9_RF	1 [nan-nan]	0	1	0.665	1	1	1	1	1	1	1
Zscore_PCC_RFE_9_LR	0.8812 [0.8588-0.9035]	0.0114	0.8767	0.5123	0.6128	0.8064	1.0098	0.8015	0.8113	0.8094	0.8034
Zscore_PCC_RFE_9_LRLasso	0.8789 [0.8563-0.9015]	0.0115	0.8741	0.5141	0.6005	0.8002	1.0123	0.7941	0.8064	0.804	0.7966
Zscore_PCC_RFE_9_AB	0.9999 [0.9998-1.0000]	0.0001	0.9999	0.4968	0.9927	0.9963	0.9926	1	0.9926	0.9927	1
Zscore_PCC_RFE_9_DT	1 [nan-nan]	0	1	1	1	1	1	1	1	1	1
Zscore_PCC_RFE_9_GP	0.9323 [0.9153-0.9494]	0.0087	0.9198	0.4917	0.7619	0.8799	0.9265	0.9167	0.8431	0.8539	0.9101
Zscore_PCC_RFE_9_NB	0.8225 [0.7944-0.8505]	0.0143	0.8274	0.9128	0.5221	0.761	1.0172	0.7525	0.7696	0.7656	0.7566
Zscore_PCC_RFE_10_SVM	0.8825 [0.8603-0.9048]	0.0113	0.8803	0.4737	0.6306	0.815	0.9534	0.8382	0.7917	0.8009	0.8303
Zscore_PCC_RFE_10_LDA	0.8827 [0.8606-0.9048]	0.0113	0.8795	0.5042	0.6056	0.8027	1.027	0.7892	0.8162	0.8111	0.7947
Zscore_PCC_RFE_10_AE	0.7963 [0.7663-0.8262]	0.0153	0.7873	0.5353	0.4689	0.7316	1.1544	0.6544	0.8088	0.7739	0.7006
Zscore_PCC_RFE_10_RF	1 [nan-nan]	0	1	0.645	1	1	1	1	1	1	1
Zscore_PCC_RFE_10_LR	0.8819 [0.8597-0.9041]	0.0113	0.8773	0.5171	0.6055	0.8027	1.0221	0.7917	0.8137	0.8095	0.7962
Zscore_PCC_RFE_10_LRLasso	0.8793 [0.8568-0.9018]	0.0115	0.8746	0.4953	0.5981	0.799	0.9853	0.8064	0.7917	0.7947	0.8035
Zscore_PCC_RFE_10_AB	1 [0.9999-1.0000]	0	1	0.502	0.9951	0.9975	1.0049	0.9951	1	1	0.9951
Zscore_PCC_RFE_10_DT	1 [nan-nan]	0	1	1	1	1	1	1	1	1	1
Zscore_PCC_RFE_10_GP	0.9461 [0.9313-0.9609]	0.0076	0.9396	0.4927	0.7906	0.8946	0.9412	0.924	0.8652	0.8727	0.9193
Zscore_PCC_RFE_10_NB	0.8231 [0.7951-0.8510]	0.0143	0.8309	0.963	0.5166	0.7561	1.1299	0.6912	0.8211	0.7944	0.7267
Zscore_PCC_RFE_11_SVM	0.8896 [0.8680-0.9112]	0.011	0.8853	0.4644	0.6334	0.8162	0.9412	0.8456	0.7868	0.7986	0.8359
Zscore_PCC_RFE_11_LDA	0.889 [0.8675-0.9104]	0.0109	0.8874	0.4826	0.6226	0.8113	1.0098	0.8064	0.8162	0.8144	0.8083
Zscore_PCC_RFE_11_AE	0.7839 [0.7528-0.8150]	0.0159	0.7756	0.5633	0.463	0.7304	0.902	0.7794	0.6814	0.7098	0.7554
Zscore_PCC_RFE_11_RF	1 [nan-nan]	0	1	0.66	1	1	1	1	1	1	1
Zscore_PCC_RFE_11_LR	0.8874 [0.8658-0.9090]	0.011	0.8838	0.4584	0.6185	0.8088	0.9461	0.8358	0.7819	0.793	0.8264
Zscore_PCC_RFE_11_LRLasso	0.8846 [0.8627-0.9066]	0.0112	0.8801	0.5444	0.6086	0.8039	1.049	0.7794	0.8284	0.8196	0.7897
Zscore_PCC_RFE_11_AB	1 [nan-nan]	0	1	0.5015	1	1	1	1	1	1	1
Zscore_PCC_RFE_11_DT	1 [nan-nan]	0	1	1	1	1	1	1	1	1	1
Zscore_PCC_RFE_11_GP	0.9578 [0.9448-0.9708]	0.0066	0.9534	0.5016	0.8212	0.9105	0.9828	0.9191	0.902	0.9036	0.9177

Zscore_PCC_RFE_11_NB	0.8227 [0.7945-0.8508]	0.0143	0.829	0.9628	0.5366	0.7672	1.0931	0.7206	0.8137	0.7946	0.7444
Zscore_PCC_RFE_12_SVM	0.8972 [0.8764-0.9179]	0.0106	0.8926	0.4423	0.6372	0.8174	0.9142	0.8603	0.7745	0.7923	0.8472
Zscore_PCC_RFE_12_LDA	0.898 [0.8777-0.9184]	0.0104	0.8963	0.454	0.6496	0.8248	0.9828	0.8333	0.8162	0.8193	0.8304
Zscore_PCC_RFE_12_AE	0.8063 [0.7771-0.8356]	0.0149	0.8015	0.5543	0.4831	0.7414	1.0319	0.7255	0.7574	0.7494	0.734
Zscore_PCC_RFE_12_RF	1 [nan-nan]	0	1	0.67	1	1	1	1	1	1	1
Zscore_PCC_RFE_12_LR	0.895 [0.8743-0.9157]	0.0106	0.8909	0.5064	0.6227	0.8113	1.0245	0.799	0.8235	0.8191	0.8038
Zscore_PCC_RFE_12_LRLasso	0.8931 [0.8722-0.9140]	0.0107	0.889	0.5168	0.6202	0.81	1.0221	0.799	0.8211	0.817	0.8034
Zscore_PCC_RFE_12_AB	1 [1.0000-1.0000]	0	1	0.4992	0.9976	0.9988	0.9975	1	0.9975	0.9976	1
Zscore_PCC_RFE_12_DT	1 [nan-nan]	0	1	1	1	1	1	1	1	1	1
Zscore_PCC_RFE_12_GP	0.9653 [0.9534-0.9772]	0.0061	0.961	0.5165	0.8358	0.9179	1.0123	0.9118	0.924	0.9231	0.9128
Zscore_PCC_RFE_12_NB	0.8205 [0.7922-0.8488]	0.0144	0.8271	0.9737	0.5268	0.7623	1.0931	0.7157	0.8088	0.7892	0.7399
Zscore_PCC_RFE_13_SVM	0.9022 [0.8820-0.9224]	0.0103	0.8965	0.4359	0.6594	0.8284	0.9118	0.8725	0.7843	0.8018	0.8602
Zscore_PCC_RFE_13_LDA	0.9026 [0.8828-0.9225]	0.0101	0.9013	0.4519	0.6471	0.8235	0.9902	0.8284	0.8186	0.8204	0.8267
Zscore_PCC_RFE_13_AE	0.7918 [0.7616-0.8219]	0.0154	0.7932	0.4647	0.4635	0.7304	0.8922	0.7843	0.6765	0.708	0.7582
Zscore_PCC_RFE_13_RF	1 [nan-nan]	0	1	0.625	1	1	1	1	1	1	1
Zscore_PCC_RFE_13_LR	0.8987 [0.8783-0.9190]	0.0104	0.8944	0.5149	0.6326	0.8162	1.0294	0.8015	0.8309	0.8258	0.8071
Zscore_PCC_RFE_13_LRLasso	0.8967 [0.8762-0.9171]	0.0105	0.8929	0.5183	0.6276	0.8137	1.0245	0.8015	0.826	0.8216	0.8062
Zscore_PCC_RFE_13_AB	1 [nan-nan]	0	1	0.5022	1	1	1	1	1	1	1
Zscore_PCC_RFE_13_DT	1 [nan-nan]	0	1	1	1	1	1	1	1	1	1
Zscore_PCC_RFE_13_GP	0.9769 [0.9674-0.9863]	0.0048	0.9748	0.5012	0.8799	0.94	0.9926	0.9436	0.9363	0.9367	0.9432
Zscore_PCC_RFE_13_NB	0.8245 [0.7965-0.8525]	0.0143	0.834	0.9775	0.5334	0.7647	1.1225	0.7034	0.826	0.8017	0.7358
Zscore_PCC_RFE_14_SVM	0.9047 [0.8846-0.9247]	0.0102	0.899	0.4247	0.6742	0.8358	0.9118	0.8799	0.7917	0.8086	0.8683
Zscore_PCC_RFE_14_LDA	0.9041 [0.8845-0.9238]	0.01	0.9033	0.4589	0.652	0.826	1.0098	0.8211	0.8309	0.8292	0.8228
Zscore_PCC_RFE_14_AE	0.8107 [0.7819-0.8395]	0.0147	0.8	0.5804	0.4844	0.7377	0.8088	0.8333	0.6422	0.6996	0.7939
Zscore_PCC_RFE_14_RF	1 [nan-nan]	0	1	0.645	1	1	1	1	1	1	1
Zscore_PCC_RFE_14_LR	0.8998 [0.8796-0.9199]	0.0103	0.8952	0.4254	0.6421	0.8199	0.9142	0.8627	0.777	0.7946	0.8499
Zscore_PCC_RFE_14_LRLasso	0.8982 [0.8779-0.9185]	0.0104	0.8948	0.4944	0.625	0.8125	0.9926	0.8162	0.8088	0.8102	0.8148
Zscore_PCC_RFE_14_AB	1 [nan-nan]	0	1	0.5031	1	1	1	1	1	1	1
Zscore_PCC_RFE_14_DT	1 [nan-nan]	0	1	1	1	1	1	1	1	1	1
Zscore_PCC_RFE_14_GP	0.9806 [0.9718-0.9893]	0.0045	0.9784	0.5056	0.8922	0.9461	1.0049	0.9436	0.9485	0.9483	0.9439
Zscore_PCC_RFE_14_NB	0.8207 [0.7924-0.8490]	0.0144	0.8283	0.9805	0.5262	0.761	1.125	0.6985	0.8235	0.7983	0.732
Zscore_PCC_RFE_15_SVM	0.9069 [0.8871-0.9267]	0.0101	0.9027	0.4267	0.6716	0.8346	0.9142	0.8775	0.7917	0.8081	0.866
Zscore_PCC_RFE_15_LDA	0.906 [0.8866-0.9255]	0.0099	0.9066	0.4561	0.6593	0.8297	0.9975	0.8309	0.8284	0.8289	0.8305
Zscore_PCC_RFE_15_AE	0.8193 [0.7907-0.8479]	0.0146	0.8073	0.5003	0.5341	0.7647	0.8676	0.8309	0.6985	0.7338	0.8051
Zscore_PCC_RFE_15_RF	1 [nan-nan]	0	1	0.66	1	1	1	1	1	1	1
Zscore_PCC_RFE_15_LR	0.9011 [0.8810-0.9211]	0.0102	0.8965	0.5261	0.645	0.8223	1.0368	0.8039	0.8407	0.8346	0.8109
Zscore_PCC_RFE_15_LRLasso	0.9003 [0.8801-0.9204]	0.0103	0.8969	0.4809	0.6399	0.8199	0.973	0.8333	0.8064	0.8115	0.8287
Zscore_PCC_RFE_15_AB	1 [nan-nan]	0	1	0.5014	1	1	1	1	1	1	1
Zscore_PCC_RFE_15_DT	1 [nan-nan]	0	1	1	1	1	1	1	1	1	1
Zscore_PCC_RFE_15_GP	0.9882 [0.9822-0.9942]	0.003	0.9886	0.4966	0.9216	0.9608	0.9902	0.9657	0.9559	0.9563	0.9653
Zscore_PCC_RFE_15_NB	0.8208 [0.7926-0.8490]	0.0144	0.8292	0.9825	0.518	0.7574	1.1127	0.701	0.8137	0.7901	0.7313
Zscore_PCC_RFE_16_SVM	0.9094 [0.8900-0.9288]	0.0099	0.9056	0.4392	0.6786	0.8382	0.9216	0.8775	0.799	0.8136	0.867
Zscore_PCC_RFE_16_LDA	0.9097 [0.8907-0.9286]	0.0097	0.9084	0.4759	0.6571	0.8284	1.0294	0.8137	0.8431	0.8384	0.819
Zscore_PCC_RFE_16_AE	0.8123 [0.7835-0.8412]	0.0147	0.8159	0.4953	0.4905	0.7439	1.1054	0.6912	0.7966	0.7726	0.7206
Zscore_PCC_RFE_16_RF	1 [nan-nan]	0	1	0.655	1	1	1	1	1	1	1
Zscore_PCC_RFE_16_LR	0.9031 [0.8832-0.9231]	0.0102	0.8976	0.5013	0.6642	0.8321	0.9975	0.8333	0.8309	0.8313	0.8329
Zscore_PCC_RFE_16_LRLasso	0.9036 [0.8838-0.9234]	0.0101	0.9004	0.5124	0.6569	0.8284	1	0.8284	0.8284	0.8284	0.8284
Zscore_PCC_RFE_16_AB	1 [nan-nan]	0	1	0.5035	1	1	1	1	1	1	1
Zscore_PCC_RFE_16_DT	1 [nan-nan]	0	1	1	1	1	1	1	1	1	1
Zscore_PCC_RFE_16_GP	0.9909 [0.9856-0.9961]	0.0027	0.9915	0.5077	0.9314	0.9657	0.9951	0.9681	0.9632	0.9634	0.968
Zscore_PCC_RFE_16_NB	0.8239 [0.7960-0.8518]	0.0142	0.8298	0.9816	0.5105	0.7525	1.1471	0.6789	0.826	0.796	0.7201
Zscore_PCC_RFE_17_SVM	0.9096 [0.8902-0.9290]	0.0099	0.9058	0.4566	0.6824	0.8407	0.9461	0.8676	0.8137	0.8233	0.8601
Zscore_PCC_RFE_17_LDA	0.9103 [0.8914-0.9291]	0.0096	0.9099	0.5133	0.6529	0.826	1.0539	0.799	0.8529	0.8446	0.8093
Zscore_PCC_RFE_17_AE	0.8269 [0.7988-0.8550]	0.0143	0.825	0.5312	0.5454	0.7721	0.9314	0.8064	0.7377	0.7546	0.7921
Zscore_PCC_RFE_17_RF	1 [nan-nan]	0	1	0.655	1	1	1	1	1	1	1
Zscore_PCC_RFE_17_LR	0.9028 [0.8829-0.9228]	0.0102	0.8981	0.4717	0.6671	0.8333	0.9657	0.8505	0.8162	0.8223	0.8452
Zscore_PCC_RFE_17_LRLasso	0.9036 [0.8838-0.9233]	0.0101	0.9006	0.5085	0.6569	0.8284	1.0098	0.8235	0.8333	0.8317	0.8252
Zscore_PCC_RFE_17_AB	1 [nan-nan]	0	1	0.5055	1	1	1	1	1	1	1
Zscore_PCC_RFE_17_DT	1 [nan-nan]	0	1	1	1	1	1	1	1	1	1
Zscore_PCC_RFE_17_GP	0.9947 [0.9908-0.9986]	0.002	0.9954	0.5169	0.9535	0.9767	1.0123	0.9706	0.9828	0.9826	0.9709
Zscore_PCC_RFE_17_NB	0.8251 [0.7972-0.8529]	0.0142	0.8292	0.9817	0.5186	0.7574	1.1225	0.6961	0.8186	0.7933	0.7293
Zscore_PCC_RFE_18_SVM	0.912 [0.8930-0.9310]	0.0097	0.908	0.4501	0.6875	0.8431	0.9412	0.8725	0.8137	0.8241	0.8646
Zscore_PCC_RFE_18_LDA	0.9135 [0.8952-0.9318]	0.0093	0.9145	0.548	0.6638	0.8309	1.0784	0.7917	0.8701	0.859	0.8068
Zscore_PCC_RFE_18_AE	0.7782 [0.7468-0.8097]	0.0161	0.7621	0.5231	0.4535	0.7267	1.0221	0.7157	0.7377	0.7318	0.7218
Zscore_PCC_RFE_18_RF	1 [nan-nan]	0	1	0.655	1	1	1	1	1	1	1
Zscore_PCC_RFE_18_LR	0.9049 [0.8853-0.9245]	0.01	0.9001	0.4834	0.6545	0.8272	0.9877	0.8333	0.8211	0.8232	0.8313
Zscore_PCC_RFE_18_LRLasso	0.9072 [0.8880-0.9265]	0.0098	0.9031	0.5154	0.652	0.826	1.0147	0.8186	0.8333	0.8308	0.8213
Zscore_PCC_RFE_18_AB	1 [nan-nan]	0	1	0.5038	1	1	1	1	1	1	1
Zscore_PCC_RFE_18_DT	1 [nan-nan]	0	1	1	1	1	1	1	1	1	1
Zscore_PCC_RFE_18_GP	0.9967 [0.9943-0.9992]	0.0013	0.997	0.5266	0.9587	0.9792	1.027	0.9657	0.9926	0.9924	0.9666
Zscore_PCC_RFE_18_NB	0.8227 [0.7946-0.8509]	0.0144	0.8237	0.9895	0.5177	0.7561	1.1446	0.6838	0.8284	0.7994	0.7238
Zscore_PCC_RFE_19_SVM	0.914 [0.8953-0.9328]	0.0096	0.9109	0.4812	0.6863	0.8431	0.9951	0.8456	0.8407	0.8415	0.8448
Zscore_PCC_RFE_19_LDA	0.9141 [0.8959-0.9324]	0.0093	0.9159	0.5781	0.6637	0.8297	1.1152	0.7721	0.8873	0.8726	0.7956

Zscore_PCC_RFE_19_AE	0.8588 [0.8341-0.8835]	0.0126	0.8576	0.4756	0.5686	0.7843	1	0.7843	0.7843	0.7843	0.7843
Zscore_PCC_RFE_19_RF	1 [nan-nan]	0	1	0.66	1	1	1	1	1	1	1
Zscore_PCC_RFE_19_LR	0.9038 [0.8840-0.9235]	0.0101	0.8987	0.4816	0.6594	0.8297	0.9828	0.8382	0.8211	0.8241	0.8354
Zscore_PCC_RFE_19_LRLasso	0.9073 [0.8880-0.9266]	0.0098	0.9033	0.4323	0.6588	0.8272	0.8848	0.8848	0.7696	0.7934	0.8698
Zscore_PCC_RFE_19_AB	1 [nan-nan]	0	1	0.5002	1	1	1	1	1	1	1
Zscore_PCC_RFE_19_DT	1 [nan-nan]	0	1	1	1	1	1	1	1	1	1
Zscore_PCC_RFE_19_GP	0.9986 [0.9974-0.9997]	0.0006	0.9986	0.5229	0.9733	0.9865	1.0221	0.9755	0.9975	0.9975	0.976
Zscore_PCC_RFE_19_NB	0.8199 [0.7916-0.8482]	0.0144	0.8211	0.9899	0.4954	0.7463	1.1054	0.6936	0.799	0.7753	0.7228
Zscore_PCC_RFE_20_SVM	0.9181 [0.8999-0.9364]	0.0093	0.9138	0.4761	0.6939	0.8468	0.973	0.8603	0.8333	0.8377	0.8564
Zscore_PCC_RFE_20_LDA	0.918 [0.9002-0.9358]	0.0091	0.9203	0.5814	0.6835	0.8395	1.1152	0.7819	0.8971	0.8837	0.8044
Zscore_PCC_RFE_20_AE	0.8158 [0.7875-0.8441]	0.0144	0.8182	0.5349	0.4726	0.7353	1.0931	0.6887	0.7819	0.7595	0.7152
Zscore_PCC_RFE_20_RF	1 [nan-nan]	0	1	0.66	1	1	1	1	1	1	1
Zscore_PCC_RFE_20_LR	0.906 [0.8865-0.9255]	0.0099	0.9003	0.4622	0.6596	0.8297	0.9681	0.8456	0.8137	0.8195	0.8405
Zscore_PCC_RFE_20_LRLasso	0.9111 [0.8923-0.9299]	0.0096	0.9068	0.4749	0.6745	0.837	0.9632	0.8554	0.8186	0.8251	0.8499
Zscore_PCC_RFE_20_AB	1 [nan-nan]	0	1	0.5035	1	1	1	1	1	1	1
Zscore_PCC_RFE_20_DT	1 [nan-nan]	0	1	1	1	1	1	1	1	1	1
Zscore_PCC_RFE_20_GP	0.999 [0.9980-0.9999]	0.0005	0.999	0.5213	0.9781	0.989	1.0172	0.9804	0.9975	0.9975	0.9807
Zscore_PCC_RFE_20_NB	0.8196 [0.7913-0.8479]	0.0144	0.8198	0.9856	0.486	0.7426	1.0539	0.7157	0.7696	0.7565	0.7302
Zscore_PCC_Relief_1_SVM	0.6277 [0.5898-0.6655]	0.0193	0.621	0.5188	0.1888	0.5944	1.0319	0.5784	0.6103	0.5975	0.5914
Zscore_PCC_Relief_1_LDA	0.6199 [0.5818-0.6580]	0.0194	0.6156	0.5098	0.208	0.6029	0.8578	0.674	0.5319	0.5901	0.62
Zscore_PCC_Relief_1_AE	0.5961 [0.5574-0.6347]	0.0197	0.6031	0.5266	0.1504	0.5699	1.3701	0.3848	0.7549	0.6109	0.551
Zscore_PCC_Relief_1_RF	1 [nan-nan]	0	1	0.555	1	1	1	1	1	1	1
Zscore_PCC_Relief_1_LR	0.6336 [0.5958-0.6713]	0.0193	0.6246	0.5103	0.1996	0.5993	0.8946	0.652	0.5466	0.5898	0.611
Zscore_PCC_Relief_1_LRLasso	0.6322 [0.5944-0.6700]	0.0193	0.6235	0.5166	0.202	0.6005	0.902	0.6495	0.5515	0.5915	0.6114
Zscore_PCC_Relief_1_AB	0.8902 [0.8692-0.9112]	0.0107	0.8963	0.5001	0.5931	0.7966	0.9951	0.799	0.7941	0.7951	0.798
Zscore_PCC_Relief_1_DT	1 [nan-nan]	0	1	1	1	1	1	1	1	1	1
Zscore_PCC_Relief_1_GP	0.6401 [0.6026-0.6777]	0.0192	0.6332	0.5035	0.2154	0.6029	0.7059	0.75	0.4559	0.5795	0.6458
Zscore_PCC_Relief_1_NB	0.5619 [0.5225-0.6013]	0.0201	0.5282	0.5912	0.1647	0.5711	0.4951	0.8235	0.3186	0.5472	0.6436
Zscore_PCC_Relief_2_SVM	0.7165 [0.6818-0.7512]	0.0177	0.7198	0.5185	0.3584	0.6789	1.0539	0.652	0.7059	0.6891	0.6698
Zscore_PCC_Relief_2_LDA	0.705 [0.6698-0.7402]	0.018	0.706	0.519	0.3237	0.6618	1.0294	0.6471	0.6765	0.6667	0.6571
Zscore_PCC_Relief_2_AE	0.624 [0.5859-0.6621]	0.0195	0.6573	0.4948	0.2573	0.6103	1.5147	0.3529	0.8676	0.7273	0.5728
Zscore_PCC_Relief_2_RF	1 [nan-nan]	0	1	0.565	1	1	1	1	1	1	1
Zscore_PCC_Relief_2_LR	0.7261 [0.6917-0.7604]	0.0175	0.7308	0.5174	0.3555	0.6777	1.027	0.6642	0.6912	0.6826	0.673
Zscore_PCC_Relief_2_LRLasso	0.7259 [0.6916-0.7603]	0.0175	0.7309	0.5294	0.3538	0.6765	1.0686	0.6422	0.7108	0.6895	0.6651
Zscore_PCC_Relief_2_AB	0.948 [0.9350-0.9610]	0.0066	0.9505	0.4992	0.736	0.8676	0.9559	0.8897	0.8456	0.8521	0.8846
Zscore_PCC_Relief_2_DT	1 [nan-nan]	0	1	1	1	1	1	1	1	1	1
Zscore_PCC_Relief_2_GP	0.7406 [0.7070-0.7742]	0.0171	0.7437	0.5161	0.3921	0.6936	1.1569	0.6152	0.7721	0.7297	0.6674
Zscore_PCC_Relief_2_NB	0.591 [0.5522-0.6298]	0.0198	0.5702	0.8378	0.1374	0.5686	0.9559	0.5907	0.5466	0.5657	0.5718
Zscore_PCC_Relief_3_SVM	0.7418 [0.7083-0.7752]	0.0171	0.763	0.517	0.3806	0.69	1.0613	0.6593	0.7206	0.7023	0.679
Zscore_PCC_Relief_3_LDA	0.7228 [0.6884-0.7572]	0.0176	0.7471	0.5703	0.381	0.6789	1.3431	0.5074	0.8505	0.7724	0.6332
Zscore_PCC_Relief_3_AE	0.5968 [0.5580-0.6355]	0.0198	0.5685	0.4902	0.1735	0.5846	1.223	0.473	0.6961	0.6088	0.5691
Zscore_PCC_Relief_3_RF	1 [nan-nan]	0	1	0.635	1	1	1	1	1	1	1
Zscore_PCC_Relief_3_LR	0.7441 [0.7107-0.7775]	0.017	0.7687	0.5568	0.3957	0.6924	1.2328	0.576	0.8088	0.7508	0.6561
Zscore_PCC_Relief_3_LRLasso	0.7444 [0.7110-0.7778]	0.017	0.77	0.5357	0.3874	0.6924	1.1152	0.6348	0.75	0.7175	0.6725
Zscore_PCC_Relief_3_AB	0.983 [0.9769-0.9891]	0.0031	0.9833	0.4983	0.8542	0.9265	0.9461	0.9534	0.8995	0.9047	0.9508
Zscore_PCC_Relief_3_DT	1 [nan-nan]	0	1	1	1	1	1	1	1	1	1
Zscore_PCC_Relief_3_GP	0.7699 [0.7379-0.8019]	0.0163	0.7774	0.5173	0.4269	0.712	1.1152	0.6544	0.7696	0.7396	0.6901
Zscore_PCC_Relief_3_NB	0.6288 [0.5908-0.6667]	0.0194	0.6055	0.9769	0.2155	0.6066	1.1446	0.5343	0.6789	0.6246	0.5931
Zscore_PCC_Relief_4_SVM	0.8015 [0.7722-0.8308]	0.015	0.8103	0.5365	0.4508	0.7243	1.1005	0.674	0.7745	0.7493	0.7038
Zscore_PCC_Relief_4_LDA	0.7837 [0.7530-0.8144]	0.0157	0.794	0.5531	0.4346	0.7157	1.1225	0.6544	0.777	0.7458	0.6921
Zscore_PCC_Relief_4_AE	0.7394 [0.7061-0.7726]	0.017	0.7496	0.4901	0.343	0.6703	1.1152	0.6127	0.7279	0.6925	0.6527
Zscore_PCC_Relief_4_RF	1 [nan-nan]	0	1	0.595	1	1	1	1	1	1	1
Zscore_PCC_Relief_4_LR	0.8003 [0.7709-0.8296]	0.015	0.8104	0.5955	0.4528	0.7206	1.2255	0.6078	0.8333	0.7848	0.68
Zscore_PCC_Relief_4_LRLasso	0.8043 [0.7752-0.8335]	0.0149	0.8134	0.5674	0.4563	0.7255	1.152	0.6495	0.8015	0.7659	0.6957
Zscore_PCC_Relief_4_AB	0.9895 [0.9852-0.9938]	0.0022	0.9896	0.4995	0.8824	0.9412	0.9951	0.9436	0.9387	0.939	0.9433
Zscore_PCC_Relief_4_DT	1 [nan-nan]	0	1	1	1	1	1	1	1	1	1
Zscore_PCC_Relief_4_GP	0.8321 [0.8046-0.8597]	0.0141	0.8359	0.5136	0.5449	0.7721	1.0539	0.7451	0.799	0.7876	0.7581
Zscore_PCC_Relief_4_NB	0.6458 [0.6085-0.6831]	0.019	0.6436	0.9837	0.1963	0.598	1.049	0.5735	0.6225	0.6031	0.5935
Zscore_PCC_Relief_5_SVM	0.8252 [0.7976-0.8528]	0.0141	0.8286	0.5221	0.5027	0.7512	1.0319	0.7353	0.7672	0.7595	0.7435
Zscore_PCC_Relief_5_LDA	0.8015 [0.7721-0.8309]	0.015	0.8053	0.5554	0.4464	0.7218	1.1103	0.6667	0.777	0.7493	0.6998
Zscore_PCC_Relief_5_AE	0.6827 [0.6465-0.7190]	0.0185	0.6703	0.493	0.2794	0.6397	1.0049	0.6373	0.6422	0.6404	0.639
Zscore_PCC_Relief_5_RF	1 [nan-nan]	0	1	0.57	1	1	1	1	1	1	1
Zscore_PCC_Relief_5_LR	0.8237 [0.7961-0.8513]	0.0141	0.8283	0.4881	0.4912	0.7451	0.9363	0.777	0.7132	0.7304	0.7618
Zscore_PCC_Relief_5_LRLasso	0.8248 [0.7972-0.8523]	0.014	0.8288	0.4999	0.4983	0.7488	0.9436	0.777	0.7206	0.7355	0.7636
Zscore_PCC_Relief_5_AB	0.9944 [0.9917-0.9972]	0.0014	0.9945	0.4995	0.9216	0.9608	1.0049	0.9583	0.9632	0.9631	0.9585
Zscore_PCC_Relief_5_DT	1 [nan-nan]	0	1	1	1	1	1	1	1	1	1
Zscore_PCC_Relief_5_GP	0.853 [0.8272-0.8789]	0.0132	0.8554	0.4767	0.5732	0.7855	0.9142	0.8284	0.7426	0.763	0.8123
Zscore_PCC_Relief_5_NB	0.6244 [0.5864-0.6625]	0.0194	0.6138	0.9608	0.22	0.6005	0.5931	0.8039	0.3971	0.5714	0.6694
Zscore_PCC_Relief_6_SVM	0.8463 [0.8205-0.8721]	0.0132	0.8453	0.4765	0.5402	0.7696	0.9412	0.799	0.7402	0.7546	0.7865
Zscore_PCC_Relief_6_LDA	0.8238 [0.7961-0.8516]	0.0142	0.821	0.5539	0.4898	0.7439	1.0907	0.6985	0.7892	0.7682	0.7236
Zscore_PCC_Relief_6_AE	0.7214 [0.6869-0.7558]	0.0176	0.739	0.5117	0.3519	0.674	1.1471	0.6005	0.7475	0.704	0.6517
Zscore_PCC_Relief_6_RF	1 [nan-nan]	0	1	0.6	1	1	1	1	1	1	1
Zscore_PCC_Relief_6_LR	0.8453 [0.8196-0.8711]	0.0131	0.8463	0.5494	0.531	0.7647	1.0784	0.7255	0.8039	0.7872	0.7455

Zscore_PCC_Relief_6_LRLasso	0.8479 [0.8223-0.8734]	0.013	0.8487	0.4652	0.5407	0.7684	0.8799	0.8284	0.7083	0.7396	0.805
Zscore_PCC_Relief_6_AB	0.9985 [0.9975-0.9996]	0.0005	0.9986	0.5019	0.9587	0.9792	1.027	0.9657	0.9926	0.9924	0.9666
Zscore_PCC_Relief_6_DT	1 [nan-nan]	0	1	1	1	1	1	1	1	1	1
Zscore_PCC_Relief_6_GP	0.877 [0.8539-0.9000]	0.0117	0.8793	0.4742	0.625	0.8113	0.9118	0.8554	0.7672	0.786	0.8414
Zscore_PCC_Relief_6_NB	0.6548 [0.6175-0.6920]	0.019	0.6369	0.9704	0.2747	0.6275	0.6275	0.8137	0.4412	0.5929	0.7031
Zscore_PCC_Relief_7_SVM	0.8622 [0.8377-0.8867]	0.0125	0.8542	0.4765	0.5781	0.788	0.9142	0.8309	0.7451	0.7652	0.815
Zscore_PCC_Relief_7_LDA	0.8423 [0.8160-0.8686]	0.0134	0.8388	0.5268	0.5296	0.7647	0.9706	0.7794	0.75	0.7571	0.7727
Zscore_PCC_Relief_7_AE	0.6775 [0.6413-0.7137]	0.0185	0.6639	0.5557	0.2666	0.6262	0.6789	0.7868	0.4657	0.5955	0.6859
Zscore_PCC_Relief_7_RF	1 [nan-nan]	0	1	0.63	1	1	1	1	1	1	1
Zscore_PCC_Relief_7_LR	0.8591 [0.8345-0.8836]	0.0125	0.8547	0.5508	0.56	0.7794	1.0637	0.7475	0.8113	0.7984	0.7627
Zscore_PCC_Relief_7_LRLasso	0.8617 [0.8374-0.8860]	0.0124	0.8576	0.472	0.574	0.7855	0.8995	0.8358	0.7353	0.7595	0.8174
Zscore_PCC_Relief_7_AB	0.9997 [0.9993-1.0000]	0.0002	0.9997	0.4988	0.9853	0.9926	0.9951	0.9951	0.9902	0.9902	0.9951
Zscore_PCC_Relief_7_DT	1 [nan-nan]	0	1	1	1	1	1	1	1	1	1
Zscore_PCC_Relief_7_GP	0.8964 [0.8753-0.9176]	0.0108	0.8943	0.4951	0.6456	0.8223	0.9436	0.8505	0.7941	0.8051	0.8416
Zscore_PCC_Relief_7_NB	0.6512 [0.6139-0.6885]	0.019	0.6325	0.9903	0.2537	0.6238	0.7819	0.7328	0.5147	0.6016	0.6583
Zscore_PCC_Relief_8_SVM	0.8672 [0.8431-0.8912]	0.0123	0.8583	0.5121	0.5911	0.7953	0.9632	0.8137	0.777	0.7849	0.8066
Zscore_PCC_Relief_8_LDA	0.8496 [0.8240-0.8752]	0.0131	0.8476	0.5068	0.5569	0.7782	0.9583	0.799	0.7574	0.7671	0.7903
Zscore_PCC_Relief_8_AE	0.6614 [0.6246-0.6982]	0.0188	0.64	0.5226	0.2261	0.6115	1.1642	0.5294	0.6936	0.6334	0.5958
Zscore_PCC_Relief_8_RF	1 [nan-nan]	0	1	0.645	1	1	1	1	1	1	1
Zscore_PCC_Relief_8_LR	0.862 [0.8376-0.8863]	0.0124	0.8565	0.483	0.5649	0.7819	0.9363	0.8137	0.75	0.765	0.801
Zscore_PCC_Relief_8_LRLasso	0.8661 [0.8421-0.8901]	0.0122	0.8594	0.4807	0.5779	0.788	0.9191	0.8284	0.7475	0.7664	0.8133
Zscore_PCC_Relief_8_AB	0.9997 [0.9995-1.0000]	0.0001	0.9997	0.5012	0.9853	0.9926	1	0.9926	0.9926	0.9926	0.9926
Zscore_PCC_Relief_8_DT	1 [nan-nan]	0	1	1	1	1	1	1	1	1	1
Zscore_PCC_Relief_8_GP	0.9069 [0.8868-0.9269]	0.0102	0.9035	0.4706	0.6759	0.8358	0.8873	0.8922	0.7794	0.8018	0.8785
Zscore_PCC_Relief_8_NB	0.6502 [0.6129-0.6875]	0.019	0.627	0.9962	0.2447	0.6201	0.8088	0.7157	0.5245	0.6008	0.6485
Zscore_PCC_Relief_9_SVM	0.8722 [0.8487-0.8957]	0.012	0.8623	0.4924	0.5973	0.7978	0.924	0.8358	0.7598	0.7768	0.8223
Zscore_PCC_Relief_9_LDA	0.8516 [0.8264-0.8769]	0.0129	0.8512	0.4984	0.5449	0.7721	0.9461	0.799	0.7451	0.7581	0.7876
Zscore_PCC_Relief_9_AE	0.737 [0.7032-0.7708]	0.0172	0.7145	0.5006	0.3925	0.6961	0.9608	0.7157	0.6765	0.6887	0.7041
Zscore_PCC_Relief_9_RF	1 [nan-nan]	0	1	0.64	1	1	1	1	1	1	1
Zscore_PCC_Relief_9_LR	0.8651 [0.8411-0.8890]	0.0122	0.8594	0.4642	0.5743	0.7855	0.8946	0.8382	0.7328	0.7583	0.8192
Zscore_PCC_Relief_9_LRLasso	0.8689 [0.8453-0.8925]	0.0121	0.8626	0.4819	0.5871	0.7929	0.9338	0.826	0.7598	0.7747	0.8136
Zscore_PCC_Relief_9_AB	0.9998 [0.9996-1.0000]	0.0001	0.9998	0.5014	0.9902	0.9951	1.0049	0.9926	0.9975	0.9975	0.9927
Zscore_PCC_Relief_9_DT	1 [nan-nan]	0	1	1	1	1	1	1	1	1	1
Zscore_PCC_Relief_9_GP	0.9154 [0.8964-0.9343]	0.0097	0.9132	0.45	0.7137	0.8517	0.8309	0.9363	0.7672	0.8008	0.9233
Zscore_PCC_Relief_9_NB	0.6533 [0.6161-0.6905]	0.019	0.624	0.9981	0.2386	0.6176	0.8333	0.701	0.5343	0.6008	0.6412
Zscore_PCC_Relief_10_SVM	0.8749 [0.8517-0.8981]	0.0118	0.8651	0.428	0.6051	0.799	0.848	0.875	0.723	0.7596	0.8526
Zscore_PCC_Relief_10_LDA	0.8633 [0.8391-0.8874]	0.0123	0.8612	0.4687	0.5552	0.777	0.9314	0.8113	0.7426	0.7592	0.7974
Zscore_PCC_Relief_10_AE	0.6653 [0.6286-0.7021]	0.0187	0.6427	0.504	0.276	0.6311	0.6887	0.7868	0.4755	0.6	0.6904
Zscore_PCC_Relief_10_RF	1 [nan-nan]	0	1	0.64	1	1	1	1	1	1	1
Zscore_PCC_Relief_10_LR	0.8667 [0.8429-0.8905]	0.0121	0.8625	0.4286	0.5862	0.7892	0.8382	0.8701	0.7083	0.7489	0.845
Zscore_PCC_Relief_10_LRLasso	0.8743 [0.8513-0.8974]	0.0118	0.8682	0.482	0.5918	0.7953	0.9387	0.826	0.7647	0.7783	0.8146
Zscore_PCC_Relief_10_AB	1 [0.9999-1.0000]	0	1	0.4994	0.9951	0.9975	0.9951	1	0.9951	0.9951	1
Zscore_PCC_Relief_10_DT	1 [nan-nan]	0	1	1	1	1	1	1	1	1	1
Zscore_PCC_Relief_10_GP	0.9203 [0.9020-0.9387]	0.0093	0.9195	0.4501	0.7231	0.8566	0.8358	0.9387	0.7745	0.8063	0.9267
Zscore_PCC_Relief_10_NB	0.6624 [0.6256-0.6993]	0.0188	0.6456	0.9996	0.2332	0.6164	1.0564	0.5882	0.6446	0.6234	0.6102
Zscore_PCC_Relief_11_SVM	0.8764 [0.8534-0.8995]	0.0118	0.8661	0.4784	0.6001	0.799	0.9167	0.8407	0.7574	0.776	0.8262
Zscore_PCC_Relief_11_LDA	0.8666 [0.8427-0.8904]	0.0122	0.8642	0.5351	0.5668	0.7831	1.0466	0.7598	0.8064	0.7969	0.7705
Zscore_PCC_Relief_11_AE	0.6537 [0.6166-0.6908]	0.0189	0.6369	0.5137	0.277	0.6078	0.3725	0.9216	0.2941	0.5663	0.7895
Zscore_PCC_Relief_11_RF	1 [nan-nan]	0	1	0.645	1	1	1	1	1	1	1
Zscore_PCC_Relief_11_LR	0.8676 [0.8440-0.8912]	0.0121	0.864	0.4581	0.5798	0.788	0.8848	0.8456	0.7304	0.7582	0.8255
Zscore_PCC_Relief_11_LRLasso	0.8768 [0.8541-0.8995]	0.0116	0.8715	0.5191	0.5882	0.7941	0.9951	0.7966	0.7917	0.7927	0.7956
Zscore_PCC_Relief_11_AB	1 [nan-nan]	0	1	0.4977	1	1	1	1	1	1	1
Zscore_PCC_Relief_11_DT	1 [nan-nan]	0	1	1	1	1	1	1	1	1	1
Zscore_PCC_Relief_11_GP	0.9339 [0.9176-0.9501]	0.0083	0.9348	0.4793	0.7356	0.8664	0.9142	0.9093	0.8235	0.8375	0.9008
Zscore_PCC_Relief_11_NB	0.6538 [0.6166-0.6910]	0.019	0.6239	0.9997	0.2405	0.6201	0.951	0.6446	0.5956	0.6145	0.6263
Zscore_PCC_Relief_12_SVM	0.881 [0.8583-0.9038]	0.0116	0.8708	0.4692	0.6224	0.81	0.9142	0.8529	0.7672	0.7856	0.8391
Zscore_PCC_Relief_12_LDA	0.8723 [0.8489-0.8957]	0.0119	0.87	0.5219	0.581	0.7904	1.0221	0.7794	0.8015	0.797	0.7842
Zscore_PCC_Relief_12_AE	0.7891 [0.7587-0.8196]	0.0155	0.7761	0.5201	0.4373	0.7181	0.9314	0.7525	0.6838	0.7041	0.7342
Zscore_PCC_Relief_12_RF	1 [nan-nan]	0	1	0.65	1	1	1	1	1	1	1
Zscore_PCC_Relief_12_LR	0.8702 [0.8468-0.8937]	0.012	0.8667	0.4738	0.5903	0.7941	0.9167	0.8358	0.7525	0.7715	0.8209
Zscore_PCC_Relief_12_LRLasso	0.8799 [0.8575-0.9024]	0.0115	0.8748	0.5054	0.596	0.7978	0.9632	0.8162	0.7794	0.7872	0.8092
Zscore_PCC_Relief_12_AB	1 [1.0000-1.0000]	0	1	0.5015	0.9976	0.9988	1.0025	0.9975	1	1	0.9976
Zscore_PCC_Relief_12_DT	1 [nan-nan]	0	1	1	1	1	1	1	1	1	1
Zscore_PCC_Relief_12_GP	0.9472 [0.9329-0.9616]	0.0073	0.9501	0.4995	0.7729	0.886	0.9534	0.9093	0.8627	0.8689	0.9049
Zscore_PCC_Relief_12_NB	0.6647 [0.6279-0.7015]	0.0188	0.6349	0.9999	0.243	0.6201	1.152	0.5441	0.6961	0.6416	0.6043
Zscore_PCC_Relief_13_SVM	0.8874 [0.8654-0.9095]	0.0113	0.8808	0.4674	0.6258	0.8125	0.9485	0.8382	0.7868	0.7972	0.8295
Zscore_PCC_Relief_13_LDA	0.883 [0.8607-0.9053]	0.0114	0.8798	0.4836	0.6054	0.8027	0.9926	0.8064	0.799	0.8005	0.8049
Zscore_PCC_Relief_13_AE	0.7392 [0.7057-0.7728]	0.0171	0.7174	0.507	0.378	0.6887	1.0539	0.6618	0.7157	0.6995	0.6791
Zscore_PCC_Relief_13_RF	1 [nan-nan]	0	1	0.66	1	1	1	1	1	1	1
Zscore_PCC_Relief_13_LR	0.875 [0.8521-0.8980]	0.0117	0.8737	0.4687	0.6001	0.799	0.9167	0.8407	0.7574	0.776	0.8262
Zscore_PCC_Relief_13_LRLasso	0.8867 [0.8650-0.9084]	0.0111	0.8835	0.5065	0.6203	0.81	0.973	0.8235	0.7966	0.8019	0.8186
Zscore_PCC_Relief_13_AB	1 [nan-nan]	0	1	0.5019	1	1	1	1	1	1	1
Zscore_PCC_Relief_13_DT	1 [nan-nan]	0	1	1	1	1	1	1	1	1	1

Zscore_PCC_Relief_13_GP	0.9578 [0.9450-0.9707]	0.0066	0.9614	0.5123	0.809	0.9044	1.0196	0.8946	0.9142	0.9125	0.8966
Zscore_PCC_Relief_13_NB	0.6653 [0.6286-0.7021]	0.0188	0.6425	0.9993	0.2612	0.6189	0.5858	0.826	0.4118	0.5841	0.7029
Zscore_PCC_Relief_14_SVM	0.8894 [0.8676-0.9112]	0.0111	0.8844	0.4774	0.6281	0.8137	0.9559	0.8358	0.7917	0.8005	0.8282
Zscore_PCC_Relief_14_LDA	0.8853 [0.8631-0.9075]	0.0113	0.8805	0.5208	0.6236	0.8113	1.0588	0.7819	0.8407	0.8307	0.794
Zscore_PCC_Relief_14_AE	0.6798 [0.6432-0.7163]	0.0186	0.6127	0.6455	0.2684	0.6336	1.0956	0.5858	0.6814	0.6477	0.6219
Zscore_PCC_Relief_14_RF	1 [nan-nan]	0	1	0.65	1	1	1	1	1	1	1
Zscore_PCC_Relief_14_LR	0.8745 [0.8514-0.8975]	0.0118	0.8727	0.5224	0.5959	0.7978	1.0319	0.7819	0.8137	0.8076	0.7886
Zscore_PCC_Relief_14_LRLasso	0.8877 [0.8661-0.9093]	0.011	0.8844	0.4954	0.6257	0.8125	0.9534	0.8358	0.7892	0.7986	0.8278
Zscore_PCC_Relief_14_AB	1 [nan-nan]	0	1	0.5012	1	1	1	1	1	1	1
Zscore_PCC_Relief_14_DT	1 [nan-nan]	0	1	1	1	1	1	1	1	1	1
Zscore_PCC_Relief_14_GP	0.9666 [0.9550-0.9781]	0.0059	0.9701	0.5118	0.831	0.9154	1.0172	0.9069	0.924	0.9227	0.9084
Zscore_PCC_Relief_14_NB	0.6595 [0.6223-0.6968]	0.019	0.6185	0.9999	0.2706	0.6336	0.8407	0.7132	0.5539	0.6152	0.6589
Zscore_PCC_Relief_15_SVM	0.8928 [0.8715-0.9142]	0.0109	0.8855	0.4917	0.6377	0.8186	0.9608	0.8382	0.799	0.8066	0.8316
Zscore_PCC_Relief_15_LDA	0.8905 [0.8691-0.9120]	0.0109	0.8849	0.5141	0.6202	0.81	1.0172	0.8015	0.8186	0.8155	0.8048
Zscore_PCC_Relief_15_AE	0.6738 [0.6370-0.7106]	0.0188	0.6102	0.5203	0.3021	0.6446	0.7108	0.7892	0.5	0.6122	0.7034
Zscore_PCC_Relief_15_RF	1 [nan-nan]	0	1	0.635	1	1	1	1	1	1	1
Zscore_PCC_Relief_15_LR	0.8775 [0.8548-0.9002]	0.0116	0.8743	0.5126	0.5933	0.7966	1.0245	0.7843	0.8088	0.804	0.7895
Zscore_PCC_Relief_15_LRLasso	0.8915 [0.8704-0.9126]	0.0108	0.8873	0.5016	0.6181	0.8088	0.9608	0.8284	0.7892	0.7972	0.8214
Zscore_PCC_Relief_15_AB	1 [nan-nan]	0	1	0.5012	1	1	1	1	1	1	1
Zscore_PCC_Relief_15_DT	1 [nan-nan]	0	1	1	1	1	1	1	1	1	1
Zscore_PCC_Relief_15_GP	0.9769 [0.9687-0.9852]	0.0042	0.9784	0.5118	0.8505	0.9252	1.0025	0.924	0.9265	0.9263	0.9242
Zscore_PCC_Relief_15_NB	0.6628 [0.6257-0.6998]	0.0189	0.6227	0.9999	0.2891	0.6409	0.777	0.7525	0.5294	0.6152	0.6814
Zscore_PCC_Relief_16_SVM	0.8961 [0.8753-0.9170]	0.0106	0.8939	0.5125	0.6422	0.8211	0.9853	0.8284	0.8137	0.8164	0.8259
Zscore_PCC_Relief_16_LDA	0.8933 [0.8723-0.9142]	0.0107	0.8922	0.4959	0.6226	0.8113	0.9853	0.8186	0.8039	0.8068	0.8159
Zscore_PCC_Relief_16_AE	0.8034 [0.7741-0.8326]	0.0149	0.7959	0.48	0.4654	0.7292	0.826	0.8162	0.6422	0.6952	0.7774
Zscore_PCC_Relief_16_RF	1 [nan-nan]	0	1	0.625	1	1	1	1	1	1	1
Zscore_PCC_Relief_16_LR	0.8804 [0.8581-0.9026]	0.0114	0.8809	0.4404	0.6056	0.8002	0.8701	0.8652	0.7353	0.7657	0.8451
Zscore_PCC_Relief_16_LRLasso	0.8945 [0.8739-0.9152]	0.0105	0.8936	0.5025	0.6154	0.8076	0.973	0.8211	0.7941	0.7995	0.8161
Zscore_PCC_Relief_16_AB	1 [nan-nan]	0	1	0.5015	1	1	1	1	1	1	1
Zscore_PCC_Relief_16_DT	1 [nan-nan]	0	1	1	1	1	1	1	1	1	1
Zscore_PCC_Relief_16_GP	0.9844 [0.9775-0.9913]	0.0035	0.9865	0.5223	0.8879	0.9436	1.0392	0.924	0.9632	0.9617	0.9269
Zscore_PCC_Relief_16_NB	0.661 [0.6239-0.6981]	0.0189	0.6217	1	0.2768	0.636	0.8162	0.7279	0.5441	0.6149	0.6667
Zscore_PCC_Relief_17_SVM	0.8995 [0.8790-0.9200]	0.0105	0.8964	0.4916	0.6429	0.8211	0.951	0.8456	0.7966	0.8061	0.8376
Zscore_PCC_Relief_17_LDA	0.8978 [0.8774-0.9182]	0.0104	0.8971	0.5123	0.6348	0.8174	1.0074	0.8137	0.8211	0.8198	0.8151
Zscore_PCC_Relief_17_AE	0.7249 [0.6906-0.7593]	0.0175	0.6699	0.5471	0.3382	0.6691	0.9951	0.6716	0.6667	0.6683	0.67
Zscore_PCC_Relief_17_RF	1 [nan-nan]	0	1	0.63	1	1	1	1	1	1	1
Zscore_PCC_Relief_17_LR	0.8786 [0.8561-0.9011]	0.0115	0.8782	0.486	0.6082	0.8039	0.9657	0.8211	0.7868	0.7938	0.8147
Zscore_PCC_Relief_17_LRLasso	0.8955 [0.8749-0.9160]	0.0105	0.8941	0.4509	0.6322	0.8137	0.8775	0.875	0.7525	0.7795	0.8575
Zscore_PCC_Relief_17_AB	1 [nan-nan]	0	1	0.5017	1	1	1	1	1	1	1
Zscore_PCC_Relief_17_DT	1 [nan-nan]	0	1	1	1	1	1	1	1	1	1
Zscore_PCC_Relief_17_GP	0.9874 [0.9811-0.9936]	0.0032	0.9892	0.5232	0.9052	0.9522	1.0417	0.9314	0.973	0.9719	0.9341
Zscore_PCC_Relief_17_NB	0.6594 [0.6221-0.6967]	0.019	0.6139	1	0.2952	0.6458	0.8456	0.723	0.5686	0.6263	0.6725
Zscore_PCC_Relief_18_SVM	0.9011 [0.8809-0.9213]	0.0103	0.8967	0.4899	0.6551	0.8272	0.9534	0.8505	0.8039	0.8126	0.8432
Zscore_PCC_Relief_18_LDA	0.9009 [0.8809-0.9209]	0.0102	0.8997	0.4578	0.6431	0.8211	0.9461	0.848	0.7941	0.8047	0.8394
Zscore_PCC_Relief_18_AE	0.7587 [0.7265-0.7908]	0.0164	0.7546	0.5031	0.3861	0.69	0.8211	0.7794	0.6005	0.6611	0.7313
Zscore_PCC_Relief_18_RF	1 [nan-nan]	0	1	0.635	1	1	1	1	1	1	1
Zscore_PCC_Relief_18_LR	0.8784 [0.8558-0.9010]	0.0115	0.8779	0.5024	0.6153	0.8076	0.9828	0.8162	0.799	0.8024	0.813
Zscore_PCC_Relief_18_LRLasso	0.8963 [0.8758-0.9168]	0.0105	0.8942	0.4494	0.6402	0.8174	0.8701	0.8824	0.7525	0.7809	0.8648
Zscore_PCC_Relief_18_AB	1 [nan-nan]	0	1	0.5034	1	1	1	1	1	1	1
Zscore_PCC_Relief_18_DT	1 [nan-nan]	0	1	1	1	1	1	1	1	1	1
Zscore_PCC_Relief_18_GP	0.9915 [0.9861-0.9969]	0.0027	0.9932	0.5131	0.9338	0.9669	1.0074	0.9632	0.9706	0.9704	0.9635
Zscore_PCC_Relief_18_NB	0.6566 [0.6192-0.6940]	0.0191	0.6103	1	0.2834	0.6409	0.8946	0.6936	0.5882	0.6275	0.6575
Zscore_PCC_Relief_19_SVM	0.9042 [0.8844-0.9240]	0.0101	0.9013	0.4741	0.6514	0.8248	0.924	0.8627	0.7868	0.8018	0.8515
Zscore_PCC_Relief_19_LDA	0.9037 [0.8841-0.9232]	0.01	0.9037	0.4859	0.6422	0.8211	0.9902	0.826	0.8162	0.818	0.8243
Zscore_PCC_Relief_19_AE	0.7756 [0.7443-0.8069]	0.016	0.7581	0.4584	0.426	0.7096	0.8211	0.799	0.6201	0.6778	0.7552
Zscore_PCC_Relief_19_RF	1 [nan-nan]	0	1	0.67	1	1	1	1	1	1	1
Zscore_PCC_Relief_19_LR	0.8823 [0.8602-0.9044]	0.0113	0.8821	0.507	0.6128	0.8064	0.9951	0.8088	0.8039	0.8049	0.8079
Zscore_PCC_Relief_19_LRLasso	0.901 [0.8811-0.9208]	0.0101	0.9007	0.4468	0.6456	0.8211	0.8971	0.8725	0.7696	0.7911	0.8579
Zscore_PCC_Relief_19_AB	1 [nan-nan]	0	1	0.5047	1	1	1	1	1	1	1
Zscore_PCC_Relief_19_DT	1 [nan-nan]	0	1	1	1	1	1	1	1	1	1
Zscore_PCC_Relief_19_GP	0.9949 [0.9910-0.9989]	0.002	0.9959	0.5137	0.951	0.9755	1.0098	0.9706	0.9804	0.9802	0.9709
Zscore_PCC_Relief_19_NB	0.6547 [0.6172-0.6921]	0.0191	0.6077	1	0.2801	0.6397	0.9314	0.674	0.6054	0.6307	0.65
Zscore_PCC_Relief_20_SVM	0.9039 [0.8839-0.9240]	0.0102	0.9013	0.5578	0.6556	0.8272	1.0613	0.7966	0.8578	0.8486	0.8083
Zscore_PCC_Relief_20_LDA	0.9063 [0.8870-0.9256]	0.0098	0.9063	0.5526	0.6462	0.8223	1.0711	0.7868	0.8578	0.847	0.8009
Zscore_PCC_Relief_20_AE	0.7494 [0.7164-0.7824]	0.0168	0.7267	0.5069	0.3895	0.6936	0.8922	0.7475	0.6397	0.6748	0.717
Zscore_PCC_Relief_20_RF	1 [nan-nan]	0	1	0.65	1	1	1	1	1	1	1
Zscore_PCC_Relief_20_LR	0.8831 [0.8610-0.9052]	0.0113	0.8828	0.4705	0.6201	0.8088	0.9118	0.8529	0.7647	0.7838	0.8387
Zscore_PCC_Relief_20_LRLasso	0.9023 [0.8826-0.9220]	0.01	0.9022	0.4695	0.6522	0.8248	0.9093	0.8701	0.7794	0.7978	0.8571
Zscore_PCC_Relief_20_AB	1 [nan-nan]	0	1	0.5034	1	1	1	1	1	1	1
Zscore_PCC_Relief_20_DT	1 [nan-nan]	0	1	1	1	1	1	1	1	1	1
Zscore_PCC_Relief_20_GP	0.9957 [0.9918-0.9997]	0.002	0.9967	0.5171	0.9611	0.9804	1.0245	0.9681	0.9926	0.9925	0.9689
Zscore_PCC_Relief_20_NB	0.6473 [0.6096-0.6850]	0.0192	0.6027	1	0.2647	0.6311	0.8652	0.6985	0.5637	0.6156	0.6516
