# Supplementary material for: A more objective PD diagnostic model: integrating texture feature markers of cerebellar gray matter and white matter through machine learning
Source: Front Aging Neurosci. 2024 Jun 7;16:1393841. doi: 10.3389/fnagi.2024.1393841 (PMC11190310; doi:10.3389/fnagi.2024.1393841)
Supplement: Supplementary file 3 [file Data_Sheet_3.pdf]

Validation_results

	AUC	95% CIs	Std	AUC-PR	Cutoff	MCC	Acc	Youden Index	Sen	Spe	PPV	NPV
Zscore_PCC_ANOVA_1_SVM	0.5969	[0.5183-0.6754]	0.0401	0.583	0.5772	0.2135	0.6029	1.2647	0.4706	0.7353	0.64	0.5814
Zscore_PCC_ANOVA_1_LDA	0.5956	[0.5169-0.6744]	0.0402	0.584	0.5784	0.2326	0.6127	1.2451	0.4902	0.7353	0.6494	0.5906
Zscore_PCC_ANOVA_1_AE	0.5807	[0.5013-0.6602]	0.0405	0.5549	0.539	0.2338	0.6127	1.2647	0.4804	0.7451	0.6533	0.5891
Zscore_PCC_ANOVA_1_RF	0.5208	[0.4411-0.6005]	0.0407	0.5424	0.09	0.0925	0.5392	0.4706	0.8039	0.2745	0.5256	0.5833
Zscore_PCC_ANOVA_1_LR	0.5964	[0.5178-0.6750]	0.0401	0.5787	0.5873	0.2231	0.6078	1.2549	0.4804	0.7353	0.6447	0.5859
Zscore_PCC_ANOVA_1_LRLasso	0.5969	[0.5184-0.6754]	0.0401	0.579	0.5906	0.2231	0.6078	1.2549	0.4804	0.7353	0.6447	0.5859
Zscore_PCC_ANOVA_1_AB	0.5322	[0.4525-0.6119]	0.0406	0.5485	0.5033	0.1257	0.5588	1.3529	0.3824	0.7353	0.5909	0.5435
Zscore_PCC_ANOVA_1_DT	0.4853	[0.4164-0.5542]	0.0352	0.6153	2	0	0.5	2	0	1	0	0.5
Zscore_PCC_ANOVA_1_GP	0.5863	[0.5069-0.6657]	0.0405	0.5541	0.5875	0.2433	0.6176	1.2549	0.4902	0.7451	0.6579	0.5938
Zscore_PCC_ANOVA_1_NB	0.5586	[0.4788-0.6384]	0.0407	0.5364	0.5498	0.1711	0.5833	0.7745	0.6961	0.4706	0.568	0.6076
Zscore_PCC_ANOVA_2_SVM	0.6935	[0.6215-0.7655]	0.0367	0.6952	0.3813	0.3419	0.6618	0.6765	0.8235	0.5	0.6222	0.7391
Zscore_PCC_ANOVA_2_LDA	0.6696	[0.5952-0.7439]	0.0379	0.6368	0.4585	0.312	0.6471	0.6667	0.8137	0.4804	0.6103	0.7206
Zscore_PCC_ANOVA_2_AE	0.6794	[0.6062-0.7525]	0.0373	0.6694	0.4553	0.2948	0.6422	0.7353	0.7745	0.5098	0.6124	0.6933
Zscore_PCC_ANOVA_2_RF	0.6621	[0.5876-0.7366]	0.038	0.6642	0.585	0.295	0.6471	1.0784	0.6078	0.6863	0.6596	0.6364
Zscore_PCC_ANOVA_2_LR	0.6875	[0.6152-0.7599]	0.0369	0.6939	0.6372	0.2948	0.6422	1.2647	0.5098	0.7745	0.6933	0.6124
Zscore_PCC_ANOVA_2_LRLasso	0.6865	[0.6140-0.7590]	0.037	0.6898	0.6419	0.2948	0.6422	1.2647	0.5098	0.7745	0.6933	0.6124
Zscore_PCC_ANOVA_2_AB	0.6318	[0.5557-0.7079]	0.0388	0.6229	0.4944	0.2383	0.6127	0.6765	0.7745	0.451	0.5852	0.6667
Zscore_PCC_ANOVA_2_DT	0.549	[0.4805-0.6175]	0.0349	0.6678	1	0.0982	0.549	0.9412	0.5784	0.5196	0.5463	0.5521
Zscore_PCC_ANOVA_2_GP	0.6788	[0.6052-0.7524]	0.0376	0.6584	0.5823	0.3199	0.6569	1.1961	0.5588	0.7549	0.6951	0.6311
Zscore_PCC_ANOVA_2_NB	0.6454	[0.5692-0.7217]	0.0389	0.6075	0.7452	0.2894	0.6422	1.1863	0.549	0.7353	0.6747	0.6198
Zscore_PCC_ANOVA_3_SVM	0.7198	[0.6502-0.7895]	0.0355	0.7298	0.6444	0.3653	0.6716	1.3431	0.5	0.8431	0.7612	0.6277
Zscore_PCC_ANOVA_3_LDA	0.7034	[0.6318-0.7750]	0.0365	0.7019	0.6055	0.3506	0.6716	1.2059	0.5686	0.7745	0.716	0.6423
Zscore_PCC_ANOVA_3_AE	0.7009	[0.6287-0.7731]	0.0368	0.7149	0.4782	0.3772	0.6863	0.8431	0.7647	0.6078	0.661	0.7209
Zscore_PCC_ANOVA_3_RF	0.7061	[0.6346-0.7776]	0.0365	0.7086	0.645	0.3907	0.6912	1.2059	0.5882	0.7941	0.7407	0.6585
Zscore_PCC_ANOVA_3_LR	0.7223	[0.6530-0.7917]	0.0354	0.7347	0.6109	0.3762	0.6814	1.2647	0.549	0.8137	0.7467	0.6434
Zscore_PCC_ANOVA_3_LRLasso	0.7222	[0.6529-0.7916]	0.0354	0.7333	0.636	0.3693	0.6765	1.2941	0.5294	0.8235	0.75	0.6364
Zscore_PCC_ANOVA_3_AB	0.6475	[0.5723-0.7228]	0.0384	0.6388	0.4931	0.2724	0.6275	0.6471	0.8039	0.451	0.5942	0.697
Zscore_PCC_ANOVA_3_DT	0.5882	[0.5207-0.6558]	0.0345	0.6995	1	0.1773	0.5882	0.902	0.6373	0.5392	0.5804	0.5978
Zscore_PCC_ANOVA_3_GP	0.7441	[0.6767-0.8116]	0.0344	0.7464	0.5313	0.413	0.7059	1.0784	0.6667	0.7451	0.7234	0.6909
Zscore_PCC_ANOVA_3_NB	0.6988	[0.6262-0.7713]	0.037	0.6809	0.7342	0.353	0.6765	0.9804	0.6863	0.6667	0.6731	0.68
Zscore_PCC_ANOVA_4_SVM	0.7687	[0.7041-0.8333]	0.0329	0.7728	0.4598	0.4417	0.7206	0.951	0.7451	0.6961	0.7103	0.732
Zscore_PCC_ANOVA_4_LDA	0.7518	[0.6846-0.8191]	0.0343	0.7508	0.5445	0.4619	0.7304	1.0686	0.6961	0.7647	0.7474	0.7156
Zscore_PCC_ANOVA_4_AE	0.609	[0.5307-0.6873]	0.0399	0.6053	0.48	0.2891	0.6373	1.3137	0.4804	0.7941	0.7	0.6045
Zscore_PCC_ANOVA_4_RF	0.7302	[0.6618-0.7986]	0.0349	0.7254	0.72	0.3772	0.6765	1.3529	0.5	0.8529	0.7727	0.6304
Zscore_PCC_ANOVA_4_LR	0.7674	[0.7027-0.8321]	0.033	0.7709	0.5133	0.4317	0.7157	1.0392	0.6961	0.7353	0.7245	0.7075
Zscore_PCC_ANOVA_4_LRLasso	0.7636	[0.6984-0.8287]	0.0333	0.7688	0.4998	0.4314	0.7157	1	0.7157	0.7157	0.7157	0.7157
Zscore_PCC_ANOVA_4_AB	0.7014	[0.6303-0.7725]	0.0363	0.6966	0.499	0.3357	0.6667	0.8824	0.7255	0.6078	0.6491	0.6889
Zscore_PCC_ANOVA_4_DT	0.6176	[0.5510-0.6843]	0.034	0.7074	1	0.2364	0.6176	1.098	0.5686	0.6667	0.6304	0.6071
Zscore_PCC_ANOVA_4_GP	0.7651	[0.6993-0.8309]	0.0336	0.7561	0.539	0.4832	0.7402	1.1078	0.6863	0.7941	0.7692	0.7168
Zscore_PCC_ANOVA_4_NB	0.6995	[0.6267-0.7724]	0.0372	0.6683	0.8776	0.4169	0.7059	1.1569	0.6275	0.7843	0.7442	0.678
Zscore_PCC_ANOVA_5_SVM	0.8071	[0.7483-0.8659]	0.03	0.8194	0.5556	0.5055	0.75	1.1471	0.6765	0.8235	0.7931	0.7179
Zscore_PCC_ANOVA_5_LDA	0.8236	[0.7665-0.8808]	0.0291	0.8336	0.4884	0.5494	0.7745	1.0392	0.7549	0.7941	0.7857	0.7642
Zscore_PCC_ANOVA_5_AE	0.7398	[0.6729-0.8067]	0.0341	0.748	0.4525	0.3951	0.6863	0.6667	0.8529	0.5196	0.6397	0.7794
Zscore_PCC_ANOVA_5_RF	0.7675	[0.7031-0.8320]	0.0329	0.7734	0.655	0.4348	0.7108	1.2451	0.5882	0.8333	0.7792	0.6693
Zscore_PCC_ANOVA_5_LR	0.817	[0.7597-0.8743]	0.0292	0.8265	0.4197	0.5099	0.7549	0.9804	0.7647	0.7451	0.75	0.76
Zscore_PCC_ANOVA_5_LRLasso	0.8175	[0.7600-0.8750]	0.0293	0.8287	0.4425	0.5098	0.7549	1	0.7549	0.7549	0.7549	0.7549
Zscore_PCC_ANOVA_5_AB	0.7159	[0.6460-0.7858]	0.0357	0.7159	0.4966	0.3563	0.6765	0.8627	0.7451	0.6078	0.6552	0.7045
Zscore_PCC_ANOVA_5_DT	0.6422	[0.5763-0.7080]	0.0336	0.7275	1	0.2854	0.6422	1.0882	0.598	0.6863	0.6559	0.6306
Zscore_PCC_ANOVA_5_GP	0.7905	[0.7277-0.8532]	0.032	0.7908	0.5159	0.5405	0.7696	1.0686	0.7353	0.8039	0.7895	0.7523
Zscore_PCC_ANOVA_5_NB	0.725	[0.6553-0.7948]	0.0356	0.7482	0.9085	0.3971	0.6961	1.1569	0.6176	0.7745	0.7326	0.6695
Zscore_PCC_ANOVA_6_SVM	0.8205	[0.7637-0.8772]	0.0289	0.8265	0.5655	0.5331	0.7647	1.1176	0.7059	0.8235	0.8	0.7368
Zscore_PCC_ANOVA_6_LDA	0.8312	[0.7761-0.8864]	0.0281	0.8341	0.5676	0.5424	0.7696	1.1078	0.7157	0.8235	0.8022	0.7434
Zscore_PCC_ANOVA_6_AE	0.7147	[0.6434-0.7860]	0.0364	0.7035	0.4869	0.4053	0.701	1.1275	0.6373	0.7647	0.7303	0.6783
Zscore_PCC_ANOVA_6_RF	0.7741	[0.7104-0.8378]	0.0325	0.7657	0.48	0.4438	0.7206	0.8922	0.7745	0.6667	0.6991	0.7473
Zscore_PCC_ANOVA_6_LR	0.8211	[0.7648-0.8774]	0.0287	0.826	0.5545	0.5424	0.7696	1.1078	0.7157	0.8235	0.8022	0.7434
Zscore_PCC_ANOVA_6_LRLasso	0.8219	[0.7657-0.8781]	0.0287	0.8271	0.5068	0.5303	0.7647	1.0588	0.7353	0.7941	0.7812	0.75
Zscore_PCC_ANOVA_6_AB	0.7276	[0.6587-0.7964]	0.0351	0.6837	0.4869	0.3874	0.6863	0.7255	0.8235	0.549	0.6462	0.7568
Zscore_PCC_ANOVA_6_DT	0.6618	[0.5965-0.7270]	0.0333	0.7468	1	0.3235	0.6618	0.9902	0.6667	0.6569	0.6602	0.6634
Zscore_PCC_ANOVA_6_GP	0.7901	[0.7271-0.8531]	0.0322	0.7763	0.5414	0.5543	0.7745	1.1373	0.7059	0.8431	0.8182	0.7414
Zscore_PCC_ANOVA_6_NB	0.7395	[0.6713-0.8077]	0.0348	0.7392	0.8449	0.4043	0.701	0.8922	0.7549	0.6471	0.6814	0.7253
Zscore_PCC_ANOVA_7_SVM	0.8187	[0.7622-0.8752]	0.0288	0.8247	0.5575	0.502	0.75	1.0882	0.7059	0.7941	0.7742	0.7297
Zscore_PCC_ANOVA_7_LDA	0.81	[0.7512-0.8688]	0.03	0.7655	0.585	0.5041	0.75	1.1275	0.6863	0.8137	0.7865	0.7217
Zscore_PCC_ANOVA_7_AE	0.6209	[0.5438-0.6981]	0.0394	0.6095	0.6132	0.2771	0.6275	1.3922	0.4314	0.8235	0.7097	0.5915
Zscore_PCC_ANOVA_7_RF	0.7815	[0.7193-0.8436]	0.0317	0.7836	0.395	0.4619	0.7255	0.7843	0.8333	0.6176	0.6855	0.7875
Zscore_PCC_ANOVA_7_LR	0.8082	[0.7503-0.8662]	0.0296	0.8113	0.588	0.5041	0.75	1.1275	0.6863	0.8137	0.7865	0.7217
Zscore_PCC_ANOVA_7_LRLasso	0.7984	[0.7386-0.8583]	0.0305	0.7852	0.5475	0.4917	0.7451	1.0784	0.7059	0.7843	0.766	0.7273
Zscore_PCC_ANOVA_7_AB	0.7277	[0.6593-0.7960]	0.0349	0.7115	0.5096	0.3365	0.6667	1.1373	0.598	0.7353	0.6932	0.6466

Zscore_PCC_ANOVA_7_DT	0.6667 [0.6019-0.7314]	0.0331	0.7473	1	0.3344	0.6667	1.0784	0.6275	0.7059	0.6809	0.6545
Zscore_PCC_ANOVA_7_GP	0.7796 [0.7158-0.8434]	0.0325	0.7742	0.5508	0.5071	0.75	1.1667	0.6667	0.8333	0.8	0.7143
Zscore_PCC_ANOVA_7_NB	0.7366 [0.6681-0.8052]	0.035	0.7275	0.9672	0.4217	0.7059	1.2157	0.598	0.8137	0.7625	0.6694
Zscore_PCC_ANOVA_8_SVM	0.8355 [0.7814-0.8897]	0.0276	0.8328	0.4817	0.5589	0.7794	1.0098	0.7745	0.7843	0.7822	0.7767
Zscore_PCC_ANOVA_8_LDA	0.8174 [0.7590-0.8757]	0.0298	0.7644	0.4716	0.5394	0.7696	1.0294	0.7549	0.7843	0.7778	0.7619
Zscore_PCC_ANOVA_8_AE	0.7042 [0.6327-0.7756]	0.0365	0.6661	0.5054	0.3824	0.6912	1.0098	0.6863	0.6961	0.6931	0.6893
Zscore_PCC_ANOVA_8_RF	0.7901 [0.7282-0.8520]	0.0316	0.7731	0.395	0.5109	0.75	0.7941	0.8529	0.6471	0.7073	0.8148
Zscore_PCC_ANOVA_8_LR	0.829 [0.7737-0.8843]	0.0282	0.8196	0.4882	0.5394	0.7696	1.0294	0.7549	0.7843	0.7778	0.7619
Zscore_PCC_ANOVA_8_LRLasso	0.8163 [0.7587-0.8739]	0.0294	0.7982	0.5052	0.5298	0.7647	1.0392	0.7451	0.7843	0.7755	0.7547
Zscore_PCC_ANOVA_8_AB	0.7702 [0.7064-0.8341]	0.0326	0.7576	0.5053	0.4317	0.7157	1.0392	0.6961	0.7353	0.7245	0.7075
Zscore_PCC_ANOVA_8_DT	0.701 [0.6383-0.7637]	0.032	0.7744	1	0.4043	0.701	1.1078	0.6471	0.7549	0.7253	0.6814
Zscore_PCC_ANOVA_8_GP	0.7886 [0.7259-0.8514]	0.032	0.7751	0.506	0.5405	0.7696	1.0686	0.7353	0.8039	0.7895	0.7523
Zscore_PCC_ANOVA_8_NB	0.7488 [0.6820-0.8155]	0.0341	0.7515	0.9548	0.4169	0.7059	1.1569	0.6275	0.7843	0.7442	0.678
Zscore_PCC_ANOVA_9_SVM	0.8346 [0.7798-0.8893]	0.0279	0.8088	0.5205	0.5405	0.7696	1.0686	0.7353	0.8039	0.7895	0.7523
Zscore_PCC_ANOVA_9_LDA	0.8156 [0.7568-0.8744]	0.03	0.7613	0.4904	0.5405	0.7696	1.0686	0.7353	0.8039	0.7895	0.7523
Zscore_PCC_ANOVA_9_AE	0.7655 [0.7000-0.8309]	0.0334	0.7363	0.507	0.4518	0.7255	1.0588	0.6961	0.7549	0.7396	0.713
Zscore_PCC_ANOVA_9_RF	0.809 [0.7505-0.8676]	0.0299	0.7961	0.45	0.4917	0.7451	0.9216	0.7843	0.7059	0.7273	0.766
Zscore_PCC_ANOVA_9_LR	0.8257 [0.7696-0.8818]	0.0286	0.8018	0.6014	0.5227	0.7598	1.1078	0.7059	0.8137	0.7912	0.7345
Zscore_PCC_ANOVA_9_LRLasso	0.8157 [0.7576-0.8739]	0.0297	0.7779	0.4059	0.5345	0.7647	0.8627	0.8333	0.6961	0.7328	0.8068
Zscore_PCC_ANOVA_9_AB	0.7385 [0.6696-0.8073]	0.0351	0.6705	0.5063	0.4226	0.7108	1.0686	0.6765	0.7451	0.7263	0.6972
Zscore_PCC_ANOVA_9_DT	0.6814 [0.6173-0.7455]	0.0327	0.7591	1	0.3636	0.6814	1.0686	0.6471	0.7157	0.6947	0.6697
Zscore_PCC_ANOVA_9_GP	0.8064 [0.7464-0.8665]	0.0306	0.7811	0.5083	0.561	0.7794	1.0882	0.7353	0.8235	0.8065	0.7568
Zscore_PCC_ANOVA_9_NB	0.7562 [0.6905-0.8220]	0.0335	0.7502	0.9843	0.4237	0.7059	1.2353	0.5882	0.8235	0.7692	0.6667
Zscore_PCC_ANOVA_10_SVM	0.8329 [0.7778-0.8880]	0.0281	0.808	0.5053	0.5494	0.7745	1.0392	0.7549	0.7941	0.7857	0.7642
Zscore_PCC_ANOVA_10_LDA	0.8164 [0.7578-0.8750]	0.0299	0.7707	0.5599	0.5413	0.7696	1.0882	0.7255	0.8137	0.7957	0.7477
Zscore_PCC_ANOVA_10_AE	0.6845 [0.6114-0.7577]	0.0373	0.6249	0.5351	0.3336	0.6667	1.0392	0.6471	0.6863	0.6735	0.6604
Zscore_PCC_ANOVA_10_RF	0.8008 [0.7409-0.8607]	0.0305	0.7947	0.44	0.4936	0.7451	0.8824	0.8039	0.6863	0.7193	0.7778
Zscore_PCC_ANOVA_10_LR	0.8257 [0.7696-0.8819]	0.0286	0.8011	0.3971	0.5239	0.7598	0.8725	0.8235	0.6961	0.7304	0.7978
Zscore_PCC_ANOVA_10_LRLasso	0.8151 [0.7568-0.8733]	0.0297	0.7782	0.406	0.5345	0.7647	0.8627	0.8333	0.6961	0.7328	0.8068
Zscore_PCC_ANOVA_10_AB	0.7581 [0.6919-0.8243]	0.0338	0.7144	0.4995	0.4412	0.7206	0.9902	0.7255	0.7157	0.7184	0.7228
Zscore_PCC_ANOVA_10_DT	0.6422 [0.5761-0.7082]	0.0337	0.7301	1	0.2844	0.6422	1.0294	0.6275	0.6569	0.6465	0.6381
Zscore_PCC_ANOVA_10_GP	0.8035 [0.7427-0.8644]	0.031	0.7718	0.5004	0.5687	0.7843	1.0196	0.7745	0.7941	0.79	0.7788
Zscore_PCC_ANOVA_10_NB	0.7546 [0.6885-0.8207]	0.0337	0.7491	0.9838	0.4275	0.7108	1.1667	0.6275	0.7941	0.7529	0.6807
Zscore_PCC_ANOVA_11_SVM	0.8282 [0.7727-0.8838]	0.0283	0.8049	0.4754	0.5208	0.7598	0.9314	0.7941	0.7255	0.7431	0.7789
Zscore_PCC_ANOVA_11_LDA	0.8178 [0.7591-0.8764]	0.0299	0.7514	0.5056	0.55	0.7745	1.0588	0.7451	0.8039	0.7917	0.7593
Zscore_PCC_ANOVA_11_AE	0.7108 [0.6402-0.7813]	0.036	0.6649	0.59	0.3452	0.6716	1.1078	0.6176	0.7255	0.6923	0.6549
Zscore_PCC_ANOVA_11_RF	0.7988 [0.7389-0.8587]	0.0306	0.8006	0.45	0.4646	0.7304	0.8725	0.7941	0.6667	0.7043	0.764
Zscore_PCC_ANOVA_11_LR	0.8255 [0.7693-0.8817]	0.0287	0.7986	0.3927	0.5239	0.7598	0.8725	0.8235	0.6961	0.7304	0.7978
Zscore_PCC_ANOVA_11_LRLasso	0.815 [0.7567-0.8732]	0.0297	0.7781	0.4056	0.5345	0.7647	0.8627	0.8333	0.6961	0.7328	0.8068
Zscore_PCC_ANOVA_11_AB	0.7676 [0.7026-0.8327]	0.0332	0.7226	0.4995	0.4518	0.7255	0.9412	0.7549	0.6961	0.713	0.7396
Zscore_PCC_ANOVA_11_DT	0.6618 [0.5966-0.7269]	0.0332	0.7444	1	0.3239	0.6618	1.049	0.6373	0.6863	0.6701	0.6542
Zscore_PCC_ANOVA_11_GP	0.7995 [0.7383-0.8607]	0.0312	0.7703	0.5016	0.5591	0.7794	1.0294	0.7647	0.7941	0.7879	0.7714
Zscore_PCC_ANOVA_11_NB	0.7511 [0.6845-0.8176]	0.0339	0.749	0.9896	0.4275	0.7108	1.1667	0.6275	0.7941	0.7529	0.6807
Zscore_PCC_ANOVA_12_SVM	0.828 [0.7723-0.8838]	0.0284	0.8037	0.5377	0.5208	0.7598	1.0686	0.7255	0.7941	0.7789	0.7431
Zscore_PCC_ANOVA_12_LDA	0.8101 [0.7499-0.8702]	0.0307	0.7354	0.5403	0.5216	0.7598	1.0882	0.7157	0.8039	0.7849	0.7387
Zscore_PCC_ANOVA_12_AE	0.7758 [0.7112-0.8403]	0.0329	0.7574	0.5488	0.481	0.7402	0.951	0.7647	0.7157	0.729	0.7526
Zscore_PCC_ANOVA_12_RF	0.7886 [0.7271-0.8502]	0.0314	0.7784	0.38	0.4575	0.7206	0.7353	0.8529	0.5882	0.6744	0.8
Zscore_PCC_ANOVA_12_LR	0.8292 [0.7734-0.8850]	0.0284	0.8039	0.4828	0.5294	0.7647	1	0.7647	0.7647	0.7647	0.7647
Zscore_PCC_ANOVA_12_LRLasso	0.814 [0.7556-0.8724]	0.0298	0.7767	0.4057	0.5162	0.7549	0.8431	0.8333	0.6765	0.7203	0.8023
Zscore_PCC_ANOVA_12_AB	0.7558 [0.6896-0.8219]	0.0338	0.6959	0.5	0.4053	0.701	0.8725	0.7647	0.6373	0.6783	0.7303
Zscore_PCC_ANOVA_12_DT	0.6471 [0.5812-0.7129]	0.0336	0.7374	1	0.2943	0.6471	0.9608	0.6667	0.6275	0.6415	0.6531
Zscore_PCC_ANOVA_12_GP	0.7982 [0.7366-0.8597]	0.0314	0.7742	0.5064	0.5405	0.7696	1.0686	0.7353	0.8039	0.7895	0.7523
Zscore_PCC_ANOVA_12_NB	0.7546 [0.6886-0.8206]	0.0337	0.7689	0.9968	0.4275	0.7108	1.1667	0.6275	0.7941	0.7529	0.6807
Zscore_PCC_ANOVA_13_SVM	0.8334 [0.7781-0.8886]	0.0282	0.8082	0.4542	0.5405	0.7696	0.9314	0.8039	0.7353	0.7523	0.7895
Zscore_PCC_ANOVA_13_LDA	0.8153 [0.7571-0.8735]	0.0297	0.7672	0.5095	0.5303	0.7647	1.0588	0.7353	0.7941	0.7812	0.75
Zscore_PCC_ANOVA_13_AE	0.7369 [0.6692-0.8047]	0.0346	0.731	0.5023	0.3833	0.6912	1.0686	0.6569	0.7255	0.7053	0.6789
Zscore_PCC_ANOVA_13_RF	0.7972 [0.7373-0.8571]	0.0306	0.7967	0.385	0.469	0.7255	0.7255	0.8627	0.5882	0.6769	0.8108
Zscore_PCC_ANOVA_13_LR	0.8318 [0.7764-0.8872]	0.0283	0.801	0.4104	0.5405	0.7696	0.9314	0.8039	0.7353	0.7523	0.7895
Zscore_PCC_ANOVA_13_LRLasso	0.8253 [0.7690-0.8816]	0.0287	0.8042	0.5053	0.5298	0.7647	1.0392	0.7451	0.7843	0.7755	0.7547
Zscore_PCC_ANOVA_13_AB	0.7947 [0.7333-0.8561]	0.0313	0.7505	0.4986	0.4902	0.7451	1	0.7451	0.7451	0.7451	0.7451
Zscore_PCC_ANOVA_13_DT	0.6225 [0.5557-0.6894]	0.0341	0.7151	1	0.2452	0.6225	1.0294	0.6078	0.6373	0.6263	0.619
Zscore_PCC_ANOVA_13_GP	0.8016 [0.7404-0.8628]	0.0312	0.77	0.5047	0.5696	0.7843	1.0588	0.7549	0.8137	0.8021	0.7685
Zscore_PCC_ANOVA_13_NB	0.7391 [0.6713-0.8070]	0.0346	0.7399	0.9896	0.4035	0.701	0.9118	0.7451	0.6569	0.6847	0.7204
Zscore_PCC_ANOVA_14_SVM	0.8412 [0.7878-0.8947]	0.0273	0.8271	0.5	0.55	0.7745	1.0588	0.7451	0.8039	0.7917	0.7593
Zscore_PCC_ANOVA_14_LDA	0.8195 [0.7618-0.8772]	0.0295	0.7682	0.5284	0.5405	0.7696	1.0686	0.7353	0.8039	0.7895	0.7523
Zscore_PCC_ANOVA_14_AE	0.5803 [0.5011-0.6594]	0.0404	0.5355	0.6424	0.2367	0.6078	0.5882	0.8137	0.402	0.5764	0.6833
Zscore_PCC_ANOVA_14_RF	0.8159 [0.7592-0.8727]	0.0289	0.8129	0.555	0.472	0.7353	1.0784	0.6961	0.7745	0.7553	0.7182
Zscore_PCC_ANOVA_14_LR	0.8361 [0.7818-0.8905]	0.0277	0.8107	0.3813	0.5436	0.7696	0.8725	0.8333	0.7059	0.7391	0.809
Zscore_PCC_ANOVA_14_LRLasso	0.8298 [0.7744-0.8852]	0.0283	0.8076	0.5032	0.5399	0.7696	1.049	0.7451	0.7941	0.7835	0.757

Zscore_PCC_ANOVA_14_AB	0.7658 [0.7007-0.8308]	0.0332	0.7349	0.4981	0.4221	0.7108	0.951	0.7353	0.6863	0.7009	0.7216
Zscore_PCC_ANOVA_14_DT	0.6471 [0.5815-0.7126]	0.0334	0.731	1	0.2955	0.6471	1.098	0.598	0.6961	0.663	0.6339
Zscore_PCC_ANOVA_14_GP	0.8124 [0.7529-0.8719]	0.0304	0.7897	0.5004	0.5893	0.7941	1.0588	0.7647	0.8235	0.8125	0.7778
Zscore_PCC_ANOVA_14_NB	0.7453 [0.6781-0.8125]	0.0343	0.7484	0.9923	0.4138	0.7059	0.902	0.7549	0.6569	0.6875	0.7283
Zscore_PCC_ANOVA_15_SVM	0.837 [0.7826-0.8915]	0.0278	0.82	0.448	0.5601	0.7794	0.9314	0.8137	0.7451	0.7615	0.8
Zscore_PCC_ANOVA_15_LDA	0.8167 [0.7580-0.8754]	0.03	0.7583	0.5247	0.55	0.7745	1.0588	0.7451	0.8039	0.7917	0.7593
Zscore_PCC_ANOVA_15_AE	0.7291 [0.6600-0.7983]	0.0353	0.7188	0.5005	0.4275	0.7108	0.8333	0.7941	0.6275	0.6807	0.7529
Zscore_PCC_ANOVA_15_RF	0.8068 [0.7480-0.8655]	0.03	0.7833	0.36	0.4806	0.7304	0.7157	0.8725	0.5882	0.6794	0.8219
Zscore_PCC_ANOVA_15_LR	0.8367 [0.7823-0.8911]	0.0277	0.8083	0.3856	0.5726	0.7843	0.8824	0.8431	0.7255	0.7544	0.8222
Zscore_PCC_ANOVA_15_LRLasso	0.828 [0.7720-0.8839]	0.0286	0.8045	0.5033	0.5595	0.7794	1.049	0.7549	0.8039	0.7938	0.7664
Zscore_PCC_ANOVA_15_AB	0.7884 [0.7249-0.8520]	0.0324	0.7149	0.4992	0.5099	0.7549	0.9804	0.7647	0.7451	0.75	0.76
Zscore_PCC_ANOVA_15_DT	0.6569 [0.5914-0.7223]	0.0334	0.7409	1	0.314	0.6569	1.0392	0.6373	0.6765	0.6633	0.6509
Zscore_PCC_ANOVA_15_GP	0.8042 [0.7436-0.8648]	0.0309	0.7636	0.501	0.55	0.7745	1.0588	0.7451	0.8039	0.7917	0.7593
Zscore_PCC_ANOVA_15_NB	0.7355 [0.6669-0.8041]	0.035	0.7287	0.9954	0.4335	0.7157	0.902	0.7647	0.6667	0.6964	0.7391
Zscore_PCC_ANOVA_16_SVM	0.8317 [0.7761-0.8872]	0.0284	0.8076	0.4911	0.5589	0.7794	0.9902	0.7843	0.7745	0.7767	0.7822
Zscore_PCC_ANOVA_16_LDA	0.8227 [0.7649-0.8804]	0.0295	0.7685	0.5218	0.5595	0.7794	1.049	0.7549	0.8039	0.7938	0.7664
Zscore_PCC_ANOVA_16_AE	0.7332 [0.6640-0.8024]	0.0353	0.7122	0.4878	0.402	0.701	0.9902	0.7059	0.6961	0.699	0.703
Zscore_PCC_ANOVA_16_RF	0.7938 [0.7338-0.8539]	0.0306	0.7927	0.395	0.4418	0.7157	0.7843	0.8235	0.6078	0.6774	0.775
Zscore_PCC_ANOVA_16_LR	0.832 [0.7770-0.8870]	0.0281	0.8046	0.4665	0.5295	0.7647	0.9804	0.7745	0.7549	0.7596	0.77
Zscore_PCC_ANOVA_16_LRLasso	0.8253 [0.7689-0.8816]	0.0287	0.8017	0.5033	0.5399	0.7696	1.049	0.7451	0.7941	0.7835	0.757
Zscore_PCC_ANOVA_16_AB	0.7709 [0.7049-0.8368]	0.0336	0.6877	0.5088	0.4658	0.7304	1.1471	0.6569	0.8039	0.7701	0.7009
Zscore_PCC_ANOVA_16_DT	0.6765 [0.6120-0.7409]	0.0329	0.7561	1	0.3532	0.6765	1.0392	0.6569	0.6961	0.6837	0.6698
Zscore_PCC_ANOVA_16_GP	0.7995 [0.7383-0.8607]	0.0312	0.7582	0.501	0.5494	0.7745	1.0392	0.7549	0.7941	0.7857	0.7642
Zscore_PCC_ANOVA_16_NB	0.7244 [0.6550-0.7939]	0.0354	0.7337	0.998	0.3941	0.6961	0.902	0.7451	0.6471	0.6786	0.7174
Zscore_PCC_ANOVA_17_SVM	0.8264 [0.7702-0.8827]	0.0287	0.8099	0.4609	0.55	0.7745	0.9412	0.8039	0.7451	0.7593	0.7917
Zscore_PCC_ANOVA_17_LDA	0.8217 [0.7634-0.8800]	0.0298	0.7516	0.5292	0.5696	0.7843	1.0588	0.7549	0.8137	0.8021	0.7685
Zscore_PCC_ANOVA_17_AE	0.7428 [0.6753-0.8103]	0.0344	0.732	0.5411	0.4157	0.7059	0.8627	0.7745	0.6373	0.681	0.7386
Zscore_PCC_ANOVA_17_RF	0.8057 [0.7471-0.8642]	0.0299	0.8029	0.4	0.5069	0.7451	0.7451	0.8725	0.6176	0.6953	0.8289
Zscore_PCC_ANOVA_17_LR	0.8235 [0.7670-0.8801]	0.0289	0.7946	0.5463	0.5216	0.7598	1.0882	0.7157	0.8039	0.7849	0.7387
Zscore_PCC_ANOVA_17_LRLasso	0.8195 [0.7623-0.8767]	0.0292	0.7966	0.5032	0.5298	0.7647	1.0392	0.7451	0.7843	0.7755	0.7547
Zscore_PCC_ANOVA_17_AB	0.778 [0.7143-0.8417]	0.0325	0.724	0.5061	0.4608	0.7304	1.0098	0.7255	0.7353	0.7327	0.7282
Zscore_PCC_ANOVA_17_DT	0.6716 [0.6069-0.7362]	0.033	0.752	1	0.3436	0.6716	1.049	0.6471	0.6961	0.6804	0.6636
Zscore_PCC_ANOVA_17_GP	0.7934 [0.7319-0.8550]	0.0314	0.7547	0.5022	0.5202	0.7598	1.049	0.7353	0.7843	0.7732	0.7477
Zscore_PCC_ANOVA_17_NB	0.7098 [0.6388-0.7809]	0.0362	0.6853	0.9981	0.3855	0.6912	0.8725	0.7549	0.6275	0.6696	0.7191
Zscore_PCC_ANOVA_18_SVM	0.8277 [0.7720-0.8833]	0.0284	0.8032	0.5134	0.5198	0.7598	1.0294	0.7451	0.7745	0.7677	0.7524
Zscore_PCC_ANOVA_18_LDA	0.8188 [0.7602-0.8774]	0.0299	0.7532	0.4892	0.5303	0.7647	1.0588	0.7353	0.7941	0.7812	0.75
Zscore_PCC_ANOVA_18_AE	0.7372 [0.6695-0.8049]	0.0345	0.7396	0.4994	0.4232	0.7108	0.9118	0.7549	0.6667	0.6937	0.7312
Zscore_PCC_ANOVA_18_RF	0.7995 [0.7400-0.8589]	0.0303	0.7949	0.4	0.4753	0.7304	0.7549	0.8529	0.6078	0.685	0.8052
Zscore_PCC_ANOVA_18_LR	0.8277 [0.7721-0.8832]	0.0283	0.8	0.433	0.531	0.7647	0.9216	0.8039	0.7255	0.7455	0.7872
Zscore_PCC_ANOVA_18_LRLasso	0.8186 [0.7614-0.8758]	0.0292	0.7949	0.4583	0.5303	0.7647	0.9412	0.7941	0.7353	0.75	0.7812
Zscore_PCC_ANOVA_18_AB	0.7611 [0.6942-0.8281]	0.0342	0.6896	0.5129	0.4646	0.7304	1.1275	0.6667	0.7941	0.764	0.7043
Zscore_PCC_ANOVA_18_DT	0.6176 [0.5508-0.6845]	0.0341	0.7096	1	0.2357	0.6176	1.0588	0.5882	0.6471	0.625	0.6111
Zscore_PCC_ANOVA_18_GP	0.7942 [0.7326-0.8558]	0.0314	0.7528	0.5019	0.5107	0.7549	1.0588	0.7255	0.7843	0.7708	0.7407
Zscore_PCC_ANOVA_18_NB	0.708 [0.6369-0.7791]	0.0363	0.679	0.9994	0.3761	0.6863	0.8627	0.7549	0.6176	0.6638	0.7159
Zscore_PCC_ANOVA_19_SVM	0.8284 [0.7727-0.8842]	0.0284	0.7967	0.5127	0.5394	0.7696	1.0294	0.7549	0.7843	0.7778	0.7619
Zscore_PCC_ANOVA_19_LDA	0.8211 [0.7627-0.8795]	0.0298	0.7564	0.4762	0.5298	0.7647	1.0392	0.7451	0.7843	0.7755	0.7547
Zscore_PCC_ANOVA_19_AE	0.7173 [0.6466-0.7880]	0.0361	0.6579	0.4592	0.3726	0.6863	1.0196	0.6765	0.6961	0.69	0.6827
Zscore_PCC_ANOVA_19_RF	0.8046 [0.7457-0.8634]	0.03	0.8061	0.45	0.4646	0.7304	0.8725	0.7941	0.6667	0.7043	0.764
Zscore_PCC_ANOVA_19_LR	0.8262 [0.7702-0.8822]	0.0286	0.7904	0.4987	0.5202	0.7598	1.049	0.7353	0.7843	0.7732	0.7477
Zscore_PCC_ANOVA_19_LRLasso	0.8187 [0.7615-0.8760]	0.0292	0.7936	0.4872	0.5196	0.7598	0.9902	0.7647	0.7549	0.7573	0.7624
Zscore_PCC_ANOVA_19_AB	0.8028 [0.7418-0.8637]	0.0311	0.7675	0.5121	0.5345	0.7647	1.1373	0.6961	0.8333	0.8068	0.7328
Zscore_PCC_ANOVA_19_DT	0.6127 [0.5456-0.6799]	0.0343	0.7076	1	0.2256	0.6127	1.0294	0.598	0.6275	0.6162	0.6095
Zscore_PCC_ANOVA_19_GP	0.7933 [0.7315-0.8550]	0.0315	0.7564	0.5012	0.531	0.7647	1.0784	0.7255	0.8039	0.7872	0.7455
Zscore_PCC_ANOVA_19_NB	0.7131 [0.6424-0.7838]	0.0361	0.6847	0.9998	0.3928	0.6961	0.9412	0.7255	0.6667	0.6852	0.7083
Zscore_PCC_ANOVA_20_SVM	0.8221 [0.7647-0.8795]	0.0293	0.7756	0.4879	0.5295	0.7647	0.9804	0.7745	0.7549	0.7596	0.77
Zscore_PCC_ANOVA_20_LDA	0.8183 [0.7594-0.8772]	0.03	0.7561	0.5842	0.5239	0.7598	1.1275	0.6961	0.8235	0.7978	0.7304
Zscore_PCC_ANOVA_20_AE	0.5874 [0.5087-0.6660]	0.0401	0.536	0.5143	0.1992	0.598	0.8235	0.6863	0.5098	0.5833	0.619
Zscore_PCC_ANOVA_20_RF	0.8108 [0.7532-0.8684]	0.0294	0.8108	0.475	0.471	0.7353	0.9608	0.7549	0.7157	0.7264	0.7449
Zscore_PCC_ANOVA_20_LR	0.817 [0.7590-0.8749]	0.0296	0.7655	0.4538	0.5107	0.7549	0.9412	0.7843	0.7255	0.7407	0.7708
Zscore_PCC_ANOVA_20_LRLasso	0.8131 [0.7543-0.8718]	0.03	0.7643	0.4873	0.5196	0.7598	0.9902	0.7647	0.7549	0.7573	0.7624
Zscore_PCC_ANOVA_20_AB	0.7967 [0.7354-0.8580]	0.0313	0.7622	0.5168	0.5132	0.75	1.2255	0.6373	0.8627	0.8228	0.704
Zscore_PCC_ANOVA_20_DT	0.6029 [0.5355-0.6704]	0.0344	0.7001	1	0.206	0.6029	1.0294	0.5882	0.6176	0.6061	0.6
Zscore_PCC_ANOVA_20_GP	0.7943 [0.7326-0.8560]	0.0315	0.7625	0.5019	0.532	0.7647	1.098	0.7157	0.8137	0.7935	0.7411
Zscore_PCC_ANOVA_20_NB	0.7039 [0.6319-0.7758]	0.0367	0.6561	1	0.3642	0.6814	1.0882	0.6373	0.7255	0.6989	0.6667
Zscore_PCC_KW_1_SVM	0.6427 [0.5650-0.7203]	0.0396	0.5945	0.5219	0.3051	0.652	0.9118	0.6961	0.6078	0.6396	0.6667
Zscore_PCC_KW_1_LDA	0.5594 [0.4795-0.6393]	0.0408	0.5331	0.5231	0.1864	0.5931	1.0294	0.5784	0.6078	0.596	0.5905
Zscore_PCC_KW_1_AE	0.5018 [0.4205-0.5831]	0.0415	0.484	0.5226	0.1664	0.5784	0.6667	0.7451	0.4118	0.5588	0.6176
Zscore_PCC_KW_1_RF	0.6371 [0.5611-0.7132]	0.0388	0.6431	0.39	0.2553	0.6275	0.9412	0.6569	0.598	0.6204	0.6354
Zscore_PCC_KW_1_LR	0.6046 [0.5258-0.6833]	0.0402	0.5619	0.5323	0.2364	0.6176	0.902	0.6667	0.5686	0.6071	0.6304

Zscore_PCC_KW_1_LRLasso	0.6076 [0.5291-0.6860]	0.04	0.5678	0.5355	0.241	0.6176	0.7843	0.7255	0.5098	0.5968	0.65
Zscore_PCC_KW_1_AB	0.6404 [0.5644-0.7165]	0.0388	0.6266	0.5026	0.2663	0.6324	1.1078	0.5784	0.6863	0.6484	0.6195
Zscore_PCC_KW_1_DT	0.6127 [0.5456-0.6799]	0.0343	0.7116	1	0.2256	0.6127	0.9706	0.6275	0.598	0.6095	0.6162
Zscore_PCC_KW_1_GP	0.5796 [0.5003-0.6588]	0.0404	0.5366	0.5366	0.2039	0.598	1.2745	0.4608	0.7353	0.6351	0.5769
Zscore_PCC_KW_1_NB	0.5822 [0.5034-0.6610]	0.0402	0.5632	0.9739	0.1864	0.5931	1.0294	0.5784	0.6078	0.596	0.5905
Zscore_PCC_KW_2_SVM	0.6514 [0.5763-0.7265]	0.0383	0.6417	0.5969	0.3012	0.6373	1.4118	0.4314	0.8431	0.7333	0.5972
Zscore_PCC_KW_2_LDA	0.5789 [0.5001-0.6577]	0.0402	0.5664	0.5942	0.2096	0.598	1.3529	0.4216	0.7745	0.6515	0.5725
Zscore_PCC_KW_2_AE	0.6146 [0.5376-0.6916]	0.0393	0.6152	0.497	0.2639	0.6127	1.5196	0.3529	0.8725	0.7347	0.5742
Zscore_PCC_KW_2_RF	0.6748 [0.6010-0.7487]	0.0377	0.6528	0.495	0.3143	0.6569	0.9412	0.6863	0.6275	0.6481	0.6667
Zscore_PCC_KW_2_LR	0.6463 [0.5708-0.7218]	0.0385	0.6264	0.6081	0.2919	0.6324	1.4216	0.4216	0.8431	0.7288	0.5931
Zscore_PCC_KW_2_LRLasso	0.6455 [0.5700-0.7210]	0.0385	0.6335	0.6047	0.3012	0.6373	1.4118	0.4314	0.8431	0.7333	0.5972
Zscore_PCC_KW_2_AB	0.72 [0.6493-0.7907]	0.0361	0.6919	0.502	0.4291	0.7108	1.1863	0.6176	0.8039	0.759	0.6777
Zscore_PCC_KW_2_DT	0.5882 [0.5204-0.6561]	0.0346	0.6896	1	0.1765	0.5882	1.0196	0.5784	0.598	0.59	0.5865
Zscore_PCC_KW_2_GP	0.6135 [0.5353-0.6917]	0.0399	0.6069	0.5871	0.3012	0.6373	1.4118	0.4314	0.8431	0.7333	0.5972
Zscore_PCC_KW_2_NB	0.643 [0.5675-0.7186]	0.0385	0.6143	0.9818	0.2461	0.6225	0.9118	0.6667	0.5784	0.6126	0.6344
Zscore_PCC_KW_3_SVM	0.7135 [0.6436-0.7833]	0.0356	0.7138	0.3703	0.3558	0.6716	0.7353	0.8039	0.5392	0.6357	0.7333
Zscore_PCC_KW_3_LDA	0.6487 [0.5733-0.7241]	0.0385	0.6305	0.647	0.2818	0.6324	1.3431	0.4608	0.8039	0.7015	0.5985
Zscore_PCC_KW_3_AE	0.6238 [0.5464-0.7012]	0.0395	0.5713	0.4921	0.2648	0.6324	0.9706	0.6471	0.6176	0.6286	0.6364
Zscore_PCC_KW_3_RF	0.6947 [0.6216-0.7679]	0.0373	0.6924	0.585	0.3732	0.6863	1.0588	0.6569	0.7157	0.6979	0.6759
Zscore_PCC_KW_3_LR	0.7064 [0.6357-0.7770]	0.0361	0.7091	0.3703	0.3337	0.6618	0.7549	0.7843	0.5392	0.6299	0.7143
Zscore_PCC_KW_3_LRLasso	0.7071 [0.6366-0.7777]	0.036	0.7092	0.3829	0.3337	0.6618	0.7549	0.7843	0.5392	0.6299	0.7143
Zscore_PCC_KW_3_AB	0.7168 [0.6461-0.7875]	0.0361	0.6709	0.502	0.3632	0.6814	1.049	0.6569	0.7059	0.6907	0.6729
Zscore_PCC_KW_3_DT	0.5882 [0.5205-0.6560]	0.0346	0.6961	1	0.1768	0.5882	0.9412	0.6176	0.5588	0.5833	0.5938
Zscore_PCC_KW_3_GP	0.6876 [0.6146-0.7606]	0.0372	0.6735	0.526	0.3243	0.6618	1.0686	0.6275	0.6961	0.6737	0.6514
Zscore_PCC_KW_3_NB	0.6675 [0.5934-0.7417]	0.0378	0.6336	0.992	0.3043	0.652	1.049	0.6275	0.6765	0.6598	0.6449
Zscore_PCC_KW_4_SVM	0.7688 [0.7048-0.8329]	0.0327	0.7621	0.404	0.4551	0.7206	0.7549	0.8431	0.598	0.6772	0.7922
Zscore_PCC_KW_4_LDA	0.7111 [0.6400-0.7822]	0.0363	0.6707	0.469	0.3815	0.6863	0.7843	0.7941	0.5784	0.6532	0.7375
Zscore_PCC_KW_4_AE	0.7331 [0.6630-0.8031]	0.0357	0.6883	0.4755	0.4138	0.7059	0.902	0.7549	0.6569	0.6875	0.7283
Zscore_PCC_KW_4_RF	0.7432 [0.6752-0.8112]	0.0347	0.7411	0.525	0.4029	0.701	0.9314	0.7353	0.6667	0.6881	0.7158
Zscore_PCC_KW_4_LR	0.765 [0.7001-0.8298]	0.0331	0.7613	0.4481	0.4327	0.7157	0.9216	0.7549	0.6765	0.7	0.734
Zscore_PCC_KW_4_LRLasso	0.7672 [0.7027-0.8317]	0.0329	0.7642	0.4254	0.4383	0.7157	0.8235	0.8039	0.6275	0.6833	0.7619
Zscore_PCC_KW_4_AB	0.7257 [0.6561-0.7953]	0.0355	0.687	0.501	0.4121	0.7059	1.0392	0.6863	0.7255	0.7143	0.6981
Zscore_PCC_KW_4_DT	0.5931 [0.5256-0.6607]	0.0345	0.7004	1	0.1867	0.5931	0.9314	0.6275	0.5588	0.5872	0.6
Zscore_PCC_KW_4_GP	0.7594 [0.6935-0.8254]	0.0336	0.7549	0.5531	0.4553	0.7255	1.1373	0.6569	0.7941	0.7614	0.6983
Zscore_PCC_KW_4_NB	0.7094 [0.6373-0.7816]	0.0368	0.6549	0.9968	0.4327	0.7108	1.2255	0.598	0.8235	0.7722	0.672
Zscore_PCC_KW_5_SVM	0.7767 [0.7139-0.8396]	0.0321	0.7739	0.5054	0.4412	0.7206	1.0098	0.7157	0.7255	0.7228	0.7184
Zscore_PCC_KW_5_LDA	0.7296 [0.6609-0.7984]	0.0351	0.7001	0.4728	0.3692	0.6814	0.8137	0.7745	0.5882	0.6529	0.7229
Zscore_PCC_KW_5_AE	0.6032 [0.5243-0.6822]	0.0403	0.5412	0.4723	0.255	0.6275	0.9804	0.6373	0.6176	0.625	0.63
Zscore_PCC_KW_5_RF	0.7602 [0.6945-0.8259]	0.0335	0.752	0.57	0.4118	0.7059	1.0196	0.6961	0.7157	0.71	0.7019
Zscore_PCC_KW_5_LR	0.7734 [0.7098-0.8369]	0.0324	0.7731	0.5142	0.4414	0.7206	1.0294	0.7059	0.7353	0.7273	0.7143
Zscore_PCC_KW_5_LRLasso	0.7762 [0.7130-0.8394]	0.0322	0.7751	0.5127	0.4608	0.7304	1.0098	0.7255	0.7353	0.7327	0.7282
Zscore_PCC_KW_5_AB	0.733 [0.6639-0.8021]	0.0353	0.6907	0.4876	0.4649	0.7108	0.5784	0.9216	0.5	0.6483	0.8644
Zscore_PCC_KW_5_DT	0.6078 [0.5409-0.6748]	0.0342	0.6995	1	0.2167	0.6078	1.098	0.5588	0.6569	0.6196	0.5982
Zscore_PCC_KW_5_GP	0.7699 [0.7046-0.8352]	0.0333	0.7537	0.5379	0.4626	0.7304	1.0882	0.6863	0.7745	0.7527	0.7117
Zscore_PCC_KW_5_NB	0.7268 [0.6557-0.7979]	0.0363	0.6606	0.9978	0.4043	0.701	1.1078	0.6471	0.7549	0.7253	0.6814
Zscore_PCC_KW_6_SVM	0.8004 [0.7409-0.8598]	0.0303	0.8081	0.4742	0.4608	0.7304	0.9902	0.7353	0.7255	0.7282	0.7327
Zscore_PCC_KW_6_LDA	0.7785 [0.7153-0.8418]	0.0323	0.7455	0.4537	0.4646	0.7304	0.8725	0.7941	0.6667	0.7043	0.764
Zscore_PCC_KW_6_AE	0.7371 [0.6692-0.8051]	0.0347	0.7247	0.5026	0.3984	0.6961	1.1765	0.6078	0.7843	0.7381	0.6667
Zscore_PCC_KW_6_RF	0.7672 [0.7023-0.8320]	0.0331	0.7662	0.41	0.4566	0.7255	0.8431	0.8039	0.6471	0.6949	0.7674
Zscore_PCC_KW_6_LR	0.8006 [0.7409-0.8602]	0.0305	0.8055	0.3236	0.5077	0.7402	0.6765	0.902	0.5784	0.6815	0.8551
Zscore_PCC_KW_6_LRLasso	0.8021 [0.7427-0.8615]	0.0303	0.8106	0.511	0.471	0.7353	1.0392	0.7157	0.7549	0.7449	0.7264
Zscore_PCC_KW_6_AB	0.754 [0.6871-0.8209]	0.0341	0.7132	0.5013	0.4414	0.7206	1.0294	0.7059	0.7353	0.7273	0.7143
Zscore_PCC_KW_6_DT	0.5735 [0.5057-0.6413]	0.0346	0.6711	1	0.1479	0.5735	1.1078	0.5196	0.6275	0.5824	0.5664
Zscore_PCC_KW_6_GP	0.7958 [0.7348-0.8569]	0.0312	0.8063	0.545	0.4739	0.7353	1.1176	0.6765	0.7941	0.7667	0.7105
Zscore_PCC_KW_6_NB	0.7314 [0.6618-0.8011]	0.0355	0.6885	0.9988	0.4237	0.7059	1.2353	0.5882	0.8235	0.7692	0.6667
Zscore_PCC_KW_7_SVM	0.7986 [0.7388-0.8585]	0.0305	0.803	0.5171	0.4613	0.7304	1.049	0.7059	0.7549	0.7423	0.7196
Zscore_PCC_KW_7_LDA	0.7825 [0.7194-0.8455]	0.0322	0.7462	0.3867	0.5109	0.75	0.7941	0.8529	0.6471	0.7073	0.8148
Zscore_PCC_KW_7_AE	0.7338 [0.6647-0.8028]	0.0352	0.71	0.5896	0.4035	0.701	1.0882	0.6569	0.7451	0.7204	0.6847
Zscore_PCC_KW_7_RF	0.7745 [0.7112-0.8377]	0.0323	0.7769	0.49	0.4226	0.7108	0.9314	0.7451	0.6765	0.6972	0.7263
Zscore_PCC_KW_7_LR	0.7986 [0.7385-0.8587]	0.0307	0.8034	0.5059	0.4804	0.7402	1.0098	0.7353	0.7451	0.7426	0.7379
Zscore_PCC_KW_7_LRLasso	0.8024 [0.7430-0.8618]	0.0303	0.8108	0.5081	0.471	0.7353	1.0392	0.7157	0.7549	0.7449	0.7264
Zscore_PCC_KW_7_AB	0.7656 [0.6998-0.8315]	0.0336	0.6871	0.5003	0.4511	0.7255	1.0196	0.7157	0.7353	0.73	0.7212
Zscore_PCC_KW_7_DT	0.6275 [0.5608-0.6941]	0.034	0.7206	1	0.2549	0.6275	1	0.6275	0.6275	0.6275	0.6275
Zscore_PCC_KW_7_GP	0.7845 [0.7220-0.8470]	0.0319	0.7944	0.5079	0.4804	0.7402	0.9902	0.7451	0.7353	0.7379	0.7426
Zscore_PCC_KW_7_NB	0.7208 [0.6509-0.7907]	0.0357	0.6915	0.9993	0.3679	0.6814	1.1667	0.598	0.7647	0.7176	0.6555
Zscore_PCC_KW_8_SVM	0.8198 [0.7633-0.8762]	0.0288	0.8166	0.5594	0.502	0.75	1.0882	0.7059	0.7941	0.7742	0.7297
Zscore_PCC_KW_8_LDA	0.8051 [0.7454-0.8647]	0.0304	0.7618	0.3708	0.4999	0.7451	0.8039	0.8431	0.6471	0.7049	0.8049
Zscore_PCC_KW_8_AE	0.7195 [0.6498-0.7893]	0.0356	0.7139	0.4923	0.3717	0.6765	0.6863	0.8333	0.5196	0.6343	0.7571
Zscore_PCC_KW_8_RF	0.7962 [0.7362-0.8562]	0.0306	0.8035	0.405	0.4474	0.7206	0.8333	0.8039	0.6373	0.6891	0.7647

Zscore_PCC_KW_8_LR	0.822 [0.7659-0.8781]	0.0286	0.8215	0.5157	0.5303	0.7647	1.0588	0.7353	0.7941	0.7812	0.75
Zscore_PCC_KW_8_LRLasso	0.8255 [0.7701-0.8810]	0.0283	0.8271	0.5755	0.5216	0.7598	1.0882	0.7157	0.8039	0.7849	0.7387
Zscore_PCC_KW_8_AB	0.7717 [0.7076-0.8358]	0.0327	0.7352	0.508	0.4344	0.7157	1.1176	0.6569	0.7745	0.7444	0.693
Zscore_PCC_KW_8_DT	0.6373 [0.5710-0.7035]	0.0338	0.7291	1	0.2746	0.6373	0.9804	0.6471	0.6275	0.6346	0.64
Zscore_PCC_KW_8_GP	0.7834 [0.7208-0.8459]	0.0319	0.7939	0.5087	0.4815	0.7402	1.0686	0.7059	0.7745	0.7579	0.7248
Zscore_PCC_KW_8_NB	0.7467 [0.6797-0.8138]	0.0342	0.7112	0.9994	0.449	0.7206	1.1863	0.6275	0.8137	0.7711	0.686
Zscore_PCC_KW_9_SVM	0.8392 [0.7851-0.8933]	0.0276	0.8294	0.3629	0.5736	0.7794	0.7745	0.8922	0.6667	0.728	0.8608
Zscore_PCC_KW_9_LDA	0.8303 [0.7741-0.8864]	0.0287	0.7891	0.3668	0.5848	0.7892	0.8529	0.8627	0.7157	0.7521	0.8391
Zscore_PCC_KW_9_AE	0.7161 [0.6462-0.7859]	0.0356	0.6977	0.4994	0.3563	0.6765	1.1373	0.6078	0.7451	0.7045	0.6552
Zscore_PCC_KW_9_RF	0.7834 [0.7205-0.8462]	0.0321	0.7781	0.365	0.4551	0.7206	0.7549	0.8431	0.598	0.6772	0.7922
Zscore_PCC_KW_9_LR	0.8343 [0.7797-0.8889]	0.0278	0.8274	0.4906	0.5399	0.7696	1.049	0.7451	0.7941	0.7835	0.757
Zscore_PCC_KW_9_LRLasso	0.835 [0.7805-0.8895]	0.0278	0.831	0.4125	0.5543	0.7745	0.8627	0.8431	0.7059	0.7414	0.8182
Zscore_PCC_KW_9_AB	0.7735 [0.7096-0.8373]	0.0326	0.7304	0.4853	0.4486	0.7157	0.7255	0.8529	0.5784	0.6692	0.7973
Zscore_PCC_KW_9_DT	0.6667 [0.6018-0.7315]	0.0331	0.7479	1	0.3339	0.6667	1.0588	0.6373	0.6961	0.6771	0.6574
Zscore_PCC_KW_9_GP	0.7921 [0.7296-0.8546]	0.0319	0.7737	0.5059	0.55	0.7745	1.0588	0.7451	0.8039	0.7917	0.7593
Zscore_PCC_KW_9_NB	0.7488 [0.6820-0.8155]	0.0341	0.7368	0.9994	0.4183	0.7059	1.1765	0.6176	0.7941	0.75	0.675
Zscore_PCC_KW_10_SVM	0.8373 [0.7834-0.8912]	0.0275	0.8284	0.5177	0.5303	0.7647	1.0588	0.7353	0.7941	0.7812	0.75
Zscore_PCC_KW_10_LDA	0.81 [0.7501-0.8698]	0.0305	0.7431	0.4694	0.5295	0.7647	0.9804	0.7745	0.7549	0.7596	0.77
Zscore_PCC_KW_10_AE	0.7737 [0.7097-0.8378]	0.0327	0.7871	0.529	0.502	0.75	1.0882	0.7059	0.7941	0.7742	0.7297
Zscore_PCC_KW_10_RF	0.7999 [0.7399-0.8599]	0.0306	0.7923	0.45	0.4646	0.7304	0.8725	0.7941	0.6667	0.7043	0.764
Zscore_PCC_KW_10_LR	0.8304 [0.7752-0.8855]	0.0281	0.8219	0.6075	0.536	0.7647	1.1569	0.6863	0.8431	0.814	0.7288
Zscore_PCC_KW_10_LRLasso	0.8283 [0.7728-0.8838]	0.0283	0.82	0.5755	0.5345	0.7647	1.1373	0.6961	0.8333	0.8068	0.7328
Zscore_PCC_KW_10_AB	0.7339 [0.6657-0.8022]	0.0348	0.6878	0.4842	0.3898	0.6863	0.7059	0.8333	0.5392	0.6439	0.7639
Zscore_PCC_KW_10_DT	0.6765 [0.6120-0.7410]	0.0329	0.7574	1	0.3529	0.6765	1	0.6765	0.6765	0.6765	0.6765
Zscore_PCC_KW_10_GP	0.8021 [0.7412-0.8630]	0.0311	0.7753	0.502	0.5494	0.7745	1.0392	0.7549	0.7941	0.7857	0.7642
Zscore_PCC_KW_10_NB	0.7502 [0.6835-0.8169]	0.034	0.7315	0.9995	0.4275	0.7108	1.1667	0.6275	0.7941	0.7529	0.6807
Zscore_PCC_KW_11_SVM	0.838 [0.7844-0.8917]	0.0274	0.8312	0.5477	0.5331	0.7647	1.1176	0.7059	0.8235	0.8	0.7368
Zscore_PCC_KW_11_LDA	0.8122 [0.7531-0.8713]	0.0302	0.7673	0.4359	0.5399	0.7696	0.951	0.7941	0.7451	0.757	0.7835
Zscore_PCC_KW_11_AE	0.5442 [0.4648-0.6236]	0.0405	0.5282	0.4675	0.1978	0.5588	0.1961	0.9608	0.1569	0.5326	0.8
Zscore_PCC_KW_11_RF	0.7989 [0.7388-0.8589]	0.0306	0.7966	0.445	0.4729	0.7353	0.902	0.7843	0.6863	0.7143	0.7609
Zscore_PCC_KW_11_LR	0.8304 [0.7753-0.8854]	0.0281	0.8237	0.6081	0.536	0.7647	1.1569	0.6863	0.8431	0.814	0.7288
Zscore_PCC_KW_11_LRLasso	0.8283 [0.7728-0.8838]	0.0283	0.82	0.5755	0.5345	0.7647	1.1373	0.6961	0.8333	0.8068	0.7328
Zscore_PCC_KW_11_AB	0.7472 [0.6797-0.8147]	0.0345	0.7047	0.4964	0.4335	0.7157	0.902	0.7647	0.6667	0.6964	0.7391
Zscore_PCC_KW_11_DT	0.6225 [0.5558-0.6893]	0.0341	0.7139	1	0.2454	0.6225	1.049	0.598	0.6471	0.6289	0.6168
Zscore_PCC_KW_11_GP	0.7955 [0.7336-0.8573]	0.0315	0.7731	0.5161	0.5134	0.7549	1.1176	0.6961	0.8137	0.7889	0.7281
Zscore_PCC_KW_11_NB	0.7577 [0.6918-0.8236]	0.0336	0.7527	0.9999	0.4275	0.7108	1.1667	0.6275	0.7941	0.7529	0.6807
Zscore_PCC_KW_12_SVM	0.8426 [0.7894-0.8959]	0.0272	0.8343	0.5097	0.5691	0.7843	1.0392	0.7647	0.8039	0.7959	0.7736
Zscore_PCC_KW_12_LDA	0.817 [0.7583-0.8757]	0.03	0.7832	0.4727	0.549	0.7745	1	0.7745	0.7745	0.7745	0.7745
Zscore_PCC_KW_12_AE	0.7 [0.6280-0.7721]	0.0368	0.6503	0.4982	0.365	0.6765	0.7451	0.8039	0.549	0.6406	0.7368
Zscore_PCC_KW_12_RF	0.8035 [0.7440-0.8630]	0.0304	0.8033	0.54	0.4815	0.7402	1.0686	0.7059	0.7745	0.7579	0.7248
Zscore_PCC_KW_12_LR	0.8356 [0.7810-0.8903]	0.0279	0.8214	0.4859	0.5591	0.7794	1.0294	0.7647	0.7941	0.7879	0.7714
Zscore_PCC_KW_12_LRLasso	0.8356 [0.7810-0.8903]	0.0279	0.827	0.4042	0.5621	0.7794	0.8922	0.8333	0.7255	0.7522	0.8132
Zscore_PCC_KW_12_AB	0.7311 [0.6620-0.8001]	0.0352	0.6978	0.4919	0.4237	0.7059	0.7647	0.8235	0.5882	0.6667	0.7692
Zscore_PCC_KW_12_DT	0.6667 [0.6017-0.7316]	0.0331	0.7485	1	0.3336	0.6667	1.0392	0.6471	0.6863	0.6735	0.6604
Zscore_PCC_KW_12_GP	0.8 [0.7387-0.8613]	0.0313	0.7769	0.5038	0.5405	0.7696	1.0686	0.7353	0.8039	0.7895	0.7523
Zscore_PCC_KW_12_NB	0.7592 [0.6935-0.8250]	0.0335	0.7476	0.9999	0.4335	0.7157	1.098	0.6667	0.7647	0.7391	0.6964
Zscore_PCC_KW_13_SVM	0.8303 [0.7753-0.8852]	0.0281	0.8217	0.4875	0.5295	0.7647	0.9804	0.7745	0.7549	0.7596	0.77
Zscore_PCC_KW_13_LDA	0.8075 [0.7472-0.8677]	0.0307	0.7365	0.452	0.5099	0.7549	0.9804	0.7647	0.7451	0.75	0.76
Zscore_PCC_KW_13_AE	0.7263 [0.6567-0.7958]	0.0355	0.686	0.516	0.4053	0.701	1.1275	0.6373	0.7647	0.7303	0.6783
Zscore_PCC_KW_13_RF	0.8061 [0.7471-0.8651]	0.0301	0.7964	0.465	0.472	0.7353	0.9216	0.7745	0.6961	0.7182	0.7553
Zscore_PCC_KW_13_LR	0.8303 [0.7753-0.8853]	0.0281	0.8229	0.533	0.532	0.7647	1.098	0.7157	0.8137	0.7935	0.7411
Zscore_PCC_KW_13_LRLasso	0.816 [0.7582-0.8738]	0.0295	0.7971	0.4042	0.5345	0.7647	0.8627	0.8333	0.6961	0.7328	0.8068
Zscore_PCC_KW_13_AB	0.7462 [0.6783-0.8140]	0.0346	0.6744	0.4978	0.425	0.7108	0.8725	0.7745	0.6471	0.687	0.7416
Zscore_PCC_KW_13_DT	0.6863 [0.6224-0.7501]	0.0326	0.7632	1	0.3732	0.6863	1.0588	0.6569	0.7157	0.6979	0.6759
Zscore_PCC_KW_13_GP	0.792 [0.7298-0.8542]	0.0317	0.7727	0.5001	0.5303	0.7647	1.0588	0.7353	0.7941	0.7812	0.75
Zscore_PCC_KW_13_NB	0.7533 [0.6866-0.8199]	0.034	0.7455	1	0.4709	0.7304	1.2059	0.6275	0.8333	0.7901	0.6911
Zscore_PCC_KW_14_SVM	0.822 [0.7640-0.8800]	0.0296	0.7608	0.5	0.5392	0.7696	1.0098	0.7647	0.7745	0.7723	0.767
Zscore_PCC_KW_14_LDA	0.8071 [0.7468-0.8674]	0.0308	0.7397	0.4895	0.5295	0.7647	1.0196	0.7549	0.7745	0.77	0.7596
Zscore_PCC_KW_14_AE	0.755 [0.6885-0.8215]	0.0339	0.7138	0.6492	0.4183	0.7059	1.1765	0.6176	0.7941	0.75	0.675
Zscore_PCC_KW_14_RF	0.805 [0.7458-0.8642]	0.0302	0.8055	0.445	0.4832	0.7402	0.8922	0.7941	0.6863	0.7168	0.7692
Zscore_PCC_KW_14_LR	0.8175 [0.7594-0.8755]	0.0296	0.7672	0.3916	0.5227	0.7598	0.8922	0.8137	0.7059	0.7345	0.7912
Zscore_PCC_KW_14_LRLasso	0.8176 [0.7598-0.8753]	0.0295	0.7836	0.4098	0.5239	0.7598	0.8725	0.8235	0.6961	0.7304	0.7978
Zscore_PCC_KW_14_AB	0.7609 [0.6951-0.8267]	0.0336	0.7197	0.489	0.4719	0.7255	0.7059	0.8725	0.5784	0.6742	0.8194
Zscore_PCC_KW_14_DT	0.6569 [0.5914-0.7223]	0.0334	0.7418	1	0.3138	0.6569	1.0196	0.6471	0.6667	0.66	0.6538
Zscore_PCC_KW_14_GP	0.7913 [0.7297-0.8530]	0.0315	0.7851	0.5089	0.5162	0.7549	1.1569	0.6765	0.8333	0.8023	0.7203
Zscore_PCC_KW_14_NB	0.7375 [0.6684-0.8066]	0.0352	0.6993	1	0.446	0.7206	1.1471	0.6471	0.7941	0.7586	0.6923
Zscore_PCC_KW_15_SVM	0.8272 [0.7706-0.8838]	0.0289	0.7728	0.4368	0.5405	0.7696	0.9314	0.8039	0.7353	0.7523	0.7895
Zscore_PCC_KW_15_LDA	0.8156 [0.7565-0.8748]	0.0302	0.7562	0.5117	0.5399	0.7696	1.049	0.7451	0.7941	0.7835	0.757
Zscore_PCC_KW_15_AE	0.7339 [0.6640-0.8037]	0.0356	0.6749	0.5503	0.4315	0.7157	0.9804	0.7255	0.7059	0.7115	0.72

Zscore_PCC_KW_15_RF	0.8062 [0.7474-0.8650]	0.03	0.8107	0.41	0.498	0.7451	0.8235	0.8333	0.6569	0.7083	0.7976
Zscore_PCC_KW_15_LR	0.8211 [0.7639-0.8784]	0.0292	0.771	0.5166	0.5208	0.7598	1.0686	0.7255	0.7941	0.7789	0.7431
Zscore_PCC_KW_15_LRLasso	0.8172 [0.7593-0.8751]	0.0296	0.7796	0.4057	0.5253	0.7598	0.8529	0.8333	0.6863	0.7265	0.8046
Zscore_PCC_KW_15_AB	0.7266 [0.6569-0.7962]	0.0355	0.6629	0.4912	0.4016	0.6961	0.7843	0.8039	0.5882	0.6613	0.75
Zscore_PCC_KW_15_DT	0.6569 [0.5921-0.7216]	0.0331	0.738	1	0.3167	0.6569	1.1373	0.5882	0.7255	0.6818	0.6379
Zscore_PCC_KW_15_GP	0.8035 [0.7436-0.8635]	0.0306	0.802	0.5286	0.5245	0.7549	1.2353	0.6373	0.8725	0.8333	0.7063
Zscore_PCC_KW_15_NB	0.7424 [0.6740-0.8109]	0.0349	0.7027	1	0.446	0.7206	1.1471	0.6471	0.7941	0.7586	0.6923
Zscore_PCC_KW_16_SVM	0.8299 [0.7740-0.8858]	0.0285	0.7802	0.4765	0.5392	0.7696	0.9902	0.7745	0.7647	0.767	0.7723
Zscore_PCC_KW_16_LDA	0.819 [0.7593-0.8787]	0.0305	0.7419	0.4099	0.5791	0.7892	0.951	0.8137	0.7647	0.7757	0.8041
Zscore_PCC_KW_16_AE	0.7324 [0.6632-0.8016]	0.0353	0.6904	0.4776	0.4138	0.7059	0.902	0.7549	0.6569	0.6875	0.7283
Zscore_PCC_KW_16_RF	0.814 [0.7563-0.8716]	0.0294	0.8091	0.475	0.4906	0.7451	0.9608	0.7647	0.7255	0.7358	0.7551
Zscore_PCC_KW_16_LR	0.8224 [0.7654-0.8794]	0.0291	0.7728	0.4717	0.5196	0.7598	0.9902	0.7647	0.7549	0.7573	0.7624
Zscore_PCC_KW_16_LRLasso	0.8149 [0.7561-0.8737]	0.03	0.7523	0.5123	0.5198	0.7598	1.0294	0.7451	0.7745	0.7677	0.7524
Zscore_PCC_KW_16_AB	0.775 [0.7101-0.8400]	0.0331	0.7136	0.5058	0.5202	0.7598	0.951	0.7843	0.7353	0.7477	0.7732
Zscore_PCC_KW_16_DT	0.701 [0.6378-0.7641]	0.0322	0.7755	1	0.402	0.701	1.0098	0.6961	0.7059	0.703	0.699
Zscore_PCC_KW_16_GP	0.8076 [0.7484-0.8667]	0.0302	0.8063	0.504	0.5331	0.7647	1.1176	0.7059	0.8235	0.8	0.7368
Zscore_PCC_KW_16_NB	0.7387 [0.6701-0.8072]	0.035	0.7032	1	0.4275	0.7108	1.1667	0.6275	0.7941	0.7529	0.6807
Zscore_PCC_KW_17_SVM	0.8341 [0.7786-0.8896]	0.0283	0.7864	0.4725	0.5785	0.7892	0.9902	0.7941	0.7843	0.7864	0.7921
Zscore_PCC_KW_17_LDA	0.8198 [0.7603-0.8793]	0.0304	0.7438	0.4642	0.5687	0.7843	1.0196	0.7745	0.7941	0.79	0.7788
Zscore_PCC_KW_17_AE	0.7176 [0.6466-0.7886]	0.0362	0.6612	0.5451	0.4125	0.7059	0.9412	0.7353	0.6765	0.6944	0.7188
Zscore_PCC_KW_17_RF	0.8118 [0.7535-0.8702]	0.0298	0.8106	0.38	0.5221	0.7549	0.7843	0.8627	0.6471	0.7097	0.825
Zscore_PCC_KW_17_LR	0.8198 [0.7620-0.8776]	0.0295	0.7689	0.6028	0.5227	0.7598	1.1078	0.7059	0.8137	0.7912	0.7345
Zscore_PCC_KW_17_LRLasso	0.8164 [0.7579-0.8750]	0.0299	0.7623	0.5123	0.5394	0.7696	1.0294	0.7549	0.7843	0.7778	0.7619
Zscore_PCC_KW_17_AB	0.7771 [0.7127-0.8414]	0.0328	0.762	0.5053	0.4804	0.7402	1.0098	0.7353	0.7451	0.7426	0.7379
Zscore_PCC_KW_17_DT	0.6667 [0.6018-0.7315]	0.0331	0.7479	1	0.3339	0.6667	1.0588	0.6373	0.6961	0.6771	0.6574
Zscore_PCC_KW_17_GP	0.8151 [0.7566-0.8735]	0.0298	0.8065	0.5046	0.5621	0.7794	1.1078	0.7255	0.8333	0.8132	0.7522
Zscore_PCC_KW_17_NB	0.7235 [0.6533-0.7936]	0.0358	0.6896	1	0.4168	0.701	1.2647	0.5686	0.8333	0.7733	0.6589
Zscore_PCC_KW_18_SVM	0.8319 [0.7759-0.8879]	0.0286	0.7802	0.4804	0.5589	0.7794	1.0098	0.7745	0.7843	0.7822	0.7767
Zscore_PCC_KW_18_LDA	0.8154 [0.7554-0.8753]	0.0306	0.7454	0.5318	0.5704	0.7843	1.0784	0.7451	0.8235	0.8085	0.7636
Zscore_PCC_KW_18_AE	0.6915 [0.6172-0.7657]	0.0379	0.6083	0.465	0.3944	0.6912	0.7549	0.8137	0.5686	0.6535	0.7532
Zscore_PCC_KW_18_RF	0.8184 [0.7618-0.8750]	0.0289	0.8145	0.435	0.5114	0.7549	0.9216	0.7941	0.7157	0.7364	0.7766
Zscore_PCC_KW_18_LR	0.8156 [0.7574-0.8739]	0.0297	0.7661	0.5168	0.5303	0.7647	1.0588	0.7353	0.7941	0.7812	0.75
Zscore_PCC_KW_18_LRLasso	0.8156 [0.7570-0.8741]	0.0299	0.7629	0.5598	0.5208	0.7598	1.0686	0.7255	0.7941	0.7789	0.7431
Zscore_PCC_KW_18_AB	0.7473 [0.6798-0.8148]	0.0344	0.7141	0.5013	0.4414	0.7206	0.9706	0.7353	0.7059	0.7143	0.7273
Zscore_PCC_KW_18_DT	0.6814 [0.6171-0.7456]	0.0328	0.7601	1	0.3629	0.6814	1.0294	0.6667	0.6961	0.6869	0.6762
Zscore_PCC_KW_18_GP	0.8171 [0.7587-0.8755]	0.0298	0.8033	0.5013	0.5595	0.7794	1.049	0.7549	0.8039	0.7938	0.7664
Zscore_PCC_KW_18_NB	0.7231 [0.6529-0.7933]	0.0358	0.6895	1	0.4078	0.6961	1.2745	0.5588	0.8333	0.7703	0.6538
Zscore_PCC_KW_19_SVM	0.8105 [0.7496-0.8713]	0.031	0.7411	0.4825	0.5785	0.7892	1.0098	0.7843	0.7941	0.7921	0.7864
Zscore_PCC_KW_19_LDA	0.8162 [0.7560-0.8764]	0.0307	0.7402	0.4222	0.5787	0.7892	0.9706	0.8039	0.7745	0.781	0.798
Zscore_PCC_KW_19_AE	0.7357 [0.6666-0.8048]	0.0353	0.7038	0.4754	0.4118	0.7059	1	0.7059	0.7059	0.7059	0.7059
Zscore_PCC_KW_19_RF	0.8013 [0.7416-0.8611]	0.0305	0.7998	0.47	0.5006	0.75	0.951	0.7745	0.7255	0.7383	0.7629
Zscore_PCC_KW_19_LR	0.8023 [0.7411-0.8635]	0.0312	0.7321	0.4345	0.5303	0.7647	0.9412	0.7941	0.7353	0.75	0.7812
Zscore_PCC_KW_19_LRLasso	0.8033 [0.7423-0.8644]	0.0312	0.7344	0.4875	0.5196	0.7598	0.9902	0.7647	0.7549	0.7573	0.7624
Zscore_PCC_KW_19_AB	0.7496 [0.6827-0.8165]	0.0341	0.7166	0.5064	0.4024	0.701	1.049	0.6765	0.7255	0.7113	0.6916
Zscore_PCC_KW_19_DT	0.6618 [0.5969-0.7266]	0.0331	0.7426	1	0.3254	0.6618	1.1078	0.6078	0.7157	0.6813	0.646
Zscore_PCC_KW_19_GP	0.8129 [0.7534-0.8724]	0.0304	0.7907	0.5022	0.5887	0.7941	1.0392	0.7745	0.8137	0.8061	0.783
Zscore_PCC_KW_19_NB	0.72 [0.6496-0.7904]	0.0359	0.6878	1	0.4248	0.701	1.3235	0.5392	0.8627	0.7971	0.6519
Zscore_PCC_KW_20_SVM	0.8088 [0.7478-0.8698]	0.0311	0.7403	0.4654	0.5595	0.7794	0.951	0.8039	0.7549	0.7664	0.7938
Zscore_PCC_KW_20_LDA	0.8145 [0.7538-0.8752]	0.031	0.734	0.507	0.5787	0.7892	1.0294	0.7745	0.8039	0.798	0.781
Zscore_PCC_KW_20_AE	0.5978 [0.5195-0.6762]	0.04	0.5466	0.5112	0.22	0.6078	0.8039	0.7059	0.5098	0.5902	0.6341
Zscore_PCC_KW_20_RF	0.8126 [0.7550-0.8702]	0.0294	0.8101	0.45	0.491	0.7451	0.9412	0.7745	0.7157	0.7315	0.7604
Zscore_PCC_KW_20_LR	0.8001 [0.7386-0.8616]	0.0314	0.7292	0.4496	0.5303	0.7647	0.9412	0.7941	0.7353	0.75	0.7812
Zscore_PCC_KW_20_LRLasso	0.8025 [0.7412-0.8638]	0.0313	0.7333	0.4875	0.5198	0.7598	0.9706	0.7745	0.7451	0.7524	0.7677
Zscore_PCC_KW_20_AB	0.7566 [0.6905-0.8228]	0.0338	0.7199	0.4938	0.4291	0.7108	0.8137	0.8039	0.6176	0.6777	0.759
Zscore_PCC_KW_20_DT	0.701 [0.6379-0.7641]	0.0322	0.7751	1	0.4021	0.701	1.0294	0.6863	0.7157	0.7071	0.6952
Zscore_PCC_KW_20_GP	0.8104 [0.7505-0.8702]	0.0305	0.7867	0.5017	0.5887	0.7941	1.0392	0.7745	0.8137	0.8061	0.783
Zscore_PCC_KW_20_NB	0.7116 [0.6408-0.7825]	0.0362	0.6749	1	0.3657	0.6814	1.1275	0.6176	0.7451	0.7079	0.6609
Zscore_PCC_RFE_1_SVM	0.6352 [0.5580-0.7125]	0.0394	0.5856	0.5237	0.3057	0.652	0.8922	0.7059	0.598	0.6372	0.6703
Zscore_PCC_RFE_1_LDA	0.6302 [0.5527-0.7077]	0.0395	0.5847	0.5483	0.2943	0.6471	0.9608	0.6667	0.6275	0.6415	0.6531
Zscore_PCC_RFE_1_AE	0.6097 [0.5313-0.6880]	0.04	0.5704	0.5263	0.2551	0.6275	0.9608	0.6471	0.6078	0.6226	0.6327
Zscore_PCC_RFE_1_RF	0.5712 [0.4924-0.6500]	0.0402	0.5958	0.96	0.1975	0.5784	1.6078	0.2745	0.8824	0.7	0.5488
Zscore_PCC_RFE_1_LR	0.6338 [0.5565-0.7111]	0.0394	0.5831	0.5358	0.2946	0.6471	0.9412	0.6765	0.6176	0.6389	0.6562
Zscore_PCC_RFE_1_LRLasso	0.6329 [0.5556-0.7103]	0.0395	0.5838	0.5264	0.2962	0.6471	0.8824	0.7059	0.5882	0.6316	0.6667
Zscore_PCC_RFE_1_AB	0.5735 [0.4947-0.6523]	0.0402	0.5514	0.4974	0.1677	0.5784	0.6471	0.7549	0.402	0.558	0.6212
Zscore_PCC_RFE_1_DT	0.5 [0.4310-0.5690]	0.0352	0.625	2	0	0.5	2	0	1	0	0.5
Zscore_PCC_RFE_1_GP	0.6263 [0.5484-0.7042]	0.0397	0.558	0.5875	0.2847	0.6422	1.049	0.6176	0.6667	0.6495	0.6355
Zscore_PCC_RFE_1_NB	0.6092 [0.5304-0.6879]	0.0402	0.5543	0.6382	0.2746	0.6373	0.9804	0.6471	0.6275	0.6346	0.64
Zscore_PCC_RFE_2_SVM	0.733 [0.6647-0.8013]	0.0349	0.7015	0.6456	0.3723	0.6814	1.2255	0.5686	0.7941	0.7342	0.648
Zscore_PCC_RFE_2_LDA	0.7211 [0.6508-0.7914]	0.0359	0.67	0.5878	0.3824	0.6912	1.0098	0.6863	0.6961	0.6931	0.6893

Zscore_PCC_RFE_2_AE	0.7183 [0.6479-0.7887]	0.0359	0.6787	0.4905	0.3834	0.6814	1.3235	0.5196	0.8431	0.7681	0.637
Zscore_PCC_RFE_2_RF	0.6841 [0.6117-0.7566]	0.037	0.6563	0.61	0.2867	0.6422	1.1275	0.5784	0.7059	0.6629	0.6261
Zscore_PCC_RFE_2_LR	0.7319 [0.6634-0.8004]	0.0349	0.694	0.6014	0.3744	0.6863	1.098	0.6373	0.7353	0.7065	0.6696
Zscore_PCC_RFE_2_LRLasso	0.7301 [0.6613-0.7989]	0.0351	0.6912	0.6078	0.3744	0.6863	1.098	0.6373	0.7353	0.7065	0.6696
Zscore_PCC_RFE_2_AB	0.6724 [0.5989-0.7459]	0.0375	0.6421	0.4917	0.3043	0.6373	0.5686	0.8529	0.4216	0.5959	0.7414
Zscore_PCC_RFE_2_DT	0.6078 [0.5406-0.6751]	0.0343	0.7087	1	0.2159	0.6078	0.9608	0.6275	0.5882	0.6038	0.6122
Zscore_PCC_RFE_2_GP	0.7273 [0.6576-0.7970]	0.0356	0.7128	0.5821	0.3959	0.6961	1.1373	0.6275	0.7647	0.7273	0.6724
Zscore_PCC_RFE_2_NB	0.7019 [0.6293-0.7746]	0.0371	0.6409	0.745	0.3649	0.6814	1.1078	0.6275	0.7353	0.7033	0.6637
Zscore_PCC_RFE_3_SVM	0.8219 [0.7663-0.8775]	0.0283	0.8371	0.5785	0.5041	0.75	1.1275	0.6863	0.8137	0.7865	0.7217
Zscore_PCC_RFE_3_LDA	0.8231 [0.7680-0.8783]	0.0281	0.8472	0.6695	0.4931	0.7402	1.2255	0.6275	0.8529	0.8101	0.696
Zscore_PCC_RFE_3_AE	0.7678 [0.7016-0.8339]	0.0338	0.8166	0.4852	0.4709	0.7304	1.2059	0.6275	0.8333	0.7901	0.6911
Zscore_PCC_RFE_3_RF	0.7852 [0.7233-0.8471]	0.0316	0.8175	0.545	0.4646	0.7304	1.1275	0.6667	0.7941	0.764	0.7043
Zscore_PCC_RFE_3_LR	0.8223 [0.7666-0.8779]	0.0284	0.8373	0.6024	0.5162	0.7549	1.1569	0.6765	0.8333	0.8023	0.7203
Zscore_PCC_RFE_3_LRLasso	0.8217 [0.7660-0.8775]	0.0284	0.8376	0.6065	0.5071	0.75	1.1667	0.6667	0.8333	0.8	0.7143
Zscore_PCC_RFE_3_AB	0.7344 [0.6671-0.8017]	0.0343	0.739	0.5081	0.3907	0.6912	1.2059	0.5882	0.7941	0.7407	0.6585
Zscore_PCC_RFE_3_DT	0.6814 [0.6172-0.7455]	0.0327	0.7629	1	0.3632	0.6814	0.951	0.7059	0.6569	0.6729	0.6907
Zscore_PCC_RFE_3_GP	0.8104 [0.7514-0.8693]	0.0301	0.8357	0.5057	0.5107	0.7549	1.0588	0.7255	0.7843	0.7708	0.7407
Zscore_PCC_RFE_3_NB	0.7839 [0.7225-0.8453]	0.0313	0.7989	0.7444	0.4368	0.7157	1.1569	0.6373	0.7941	0.7558	0.6864
Zscore_PCC_RFE_4_SVM	0.8281 [0.7727-0.8836]	0.0283	0.8212	0.5474	0.5413	0.7696	1.0882	0.7255	0.8137	0.7957	0.7477
Zscore_PCC_RFE_4_LDA	0.8362 [0.7828-0.8897]	0.0273	0.8421	0.5949	0.5253	0.7598	1.1471	0.6863	0.8333	0.8046	0.7265
Zscore_PCC_RFE_4_AE	0.7618 [0.6958-0.8279]	0.0337	0.7063	0.4629	0.4317	0.7157	0.9608	0.7353	0.6961	0.7075	0.7245
Zscore_PCC_RFE_4_RF	0.8095 [0.7525-0.8665]	0.0291	0.8273	0.48	0.4608	0.7304	0.9902	0.7353	0.7255	0.7282	0.7327
Zscore_PCC_RFE_4_LR	0.8302 [0.7751-0.8852]	0.0281	0.8223	0.5825	0.5436	0.7696	1.1275	0.7059	0.8333	0.809	0.7391
Zscore_PCC_RFE_4_LRLasso	0.8293 [0.7742-0.8844]	0.0281	0.8224	0.584	0.5436	0.7696	1.1275	0.7059	0.8333	0.809	0.7391
Zscore_PCC_RFE_4_AB	0.7856 [0.7243-0.8469]	0.0313	0.7678	0.4929	0.4513	0.7157	0.7059	0.8627	0.5686	0.6667	0.8056
Zscore_PCC_RFE_4_DT	0.6569 [0.5914-0.7223]	0.0334	0.7418	1	0.3138	0.6569	1.0196	0.6471	0.6667	0.66	0.6538
Zscore_PCC_RFE_4_GP	0.8159 [0.7585-0.8734]	0.0293	0.8235	0.5298	0.5345	0.7647	1.1373	0.6961	0.8333	0.8068	0.7328
Zscore_PCC_RFE_4_NB	0.7883 [0.7267-0.8498]	0.0314	0.8006	0.5102	0.464	0.7255	0.7647	0.8431	0.6078	0.6825	0.7949
Zscore_PCC_RFE_5_SVM	0.8202 [0.7640-0.8765]	0.0287	0.8204	0.5603	0.5041	0.75	1.1275	0.6863	0.8137	0.7865	0.7217
Zscore_PCC_RFE_5_LDA	0.8283 [0.7735-0.8832]	0.028	0.8298	0.5908	0.5147	0.7549	1.1373	0.6863	0.8235	0.7955	0.7241
Zscore_PCC_RFE_5_AE	0.7893 [0.7279-0.8508]	0.0314	0.7915	0.4775	0.5	0.75	1.0098	0.7451	0.7549	0.7525	0.7476
Zscore_PCC_RFE_5_RF	0.8017 [0.7431-0.8603]	0.0299	0.8171	0.685	0.4543	0.7157	1.3137	0.5588	0.8725	0.8143	0.6642
Zscore_PCC_RFE_5_LR	0.825 [0.7694-0.8806]	0.0284	0.8205	0.6007	0.5253	0.7598	1.1471	0.6863	0.8333	0.8046	0.7265
Zscore_PCC_RFE_5_LRLasso	0.8232 [0.7674-0.8791]	0.0285	0.8171	0.57	0.5227	0.7598	1.1078	0.7059	0.8137	0.7912	0.7345
Zscore_PCC_RFE_5_AB	0.7578 [0.6934-0.8223]	0.0329	0.7625	0.492	0.4078	0.6961	0.7255	0.8333	0.5588	0.6538	0.7703
Zscore_PCC_RFE_5_DT	0.701 [0.6383-0.7637]	0.032	0.7744	1	0.4043	0.701	1.1078	0.6471	0.7549	0.7253	0.6814
Zscore_PCC_RFE_5_GP	0.8011 [0.7412-0.8610]	0.0306	0.8102	0.5143	0.5134	0.7549	1.1176	0.6961	0.8137	0.7889	0.7281
Zscore_PCC_RFE_5_NB	0.7768 [0.7134-0.8402]	0.0323	0.7691	0.653	0.4626	0.7304	0.9118	0.7745	0.6863	0.7117	0.7527
Zscore_PCC_RFE_6_SVM	0.8278 [0.7726-0.8829]	0.0282	0.8204	0.5	0.5295	0.7647	1.0196	0.7549	0.7745	0.77	0.7596
Zscore_PCC_RFE_6_LDA	0.8255 [0.7697-0.8814]	0.0285	0.798	0.5226	0.5303	0.7647	1.0588	0.7353	0.7941	0.7812	0.75
Zscore_PCC_RFE_6_AE	0.7559 [0.6903-0.8214]	0.0334	0.7499	0.4775	0.424	0.7108	1.1078	0.6569	0.7647	0.7363	0.6903
Zscore_PCC_RFE_6_RF	0.8142 [0.7576-0.8708]	0.0289	0.8195	0.63	0.4709	0.7304	1.2059	0.6275	0.8333	0.7901	0.6911
Zscore_PCC_RFE_6_LR	0.8251 [0.7697-0.8804]	0.0282	0.8217	0.5232	0.5303	0.7647	1.0588	0.7353	0.7941	0.7812	0.75
Zscore_PCC_RFE_6_LRLasso	0.8207 [0.7647-0.8768]	0.0286	0.8169	0.5131	0.5202	0.7598	1.049	0.7353	0.7843	0.7732	0.7477
Zscore_PCC_RFE_6_AB	0.7733 [0.7099-0.8368]	0.0324	0.739	0.5013	0.4221	0.7108	1.049	0.6863	0.7353	0.7216	0.7009
Zscore_PCC_RFE_6_DT	0.7108 [0.6487-0.7729]	0.0317	0.7823	1	0.424	0.7108	1.1078	0.6569	0.7647	0.7363	0.6903
Zscore_PCC_RFE_6_GP	0.8092 [0.7506-0.8678]	0.0299	0.8049	0.514	0.5405	0.7696	1.0686	0.7353	0.8039	0.7895	0.7523
Zscore_PCC_RFE_6_NB	0.7851 [0.7234-0.8468]	0.0315	0.7993	0.9169	0.4778	0.7304	1.2647	0.598	0.8627	0.8133	0.6822
Zscore_PCC_RFE_7_SVM	0.8236 [0.7679-0.8794]	0.0284	0.821	0.5076	0.5099	0.7549	1.0196	0.7451	0.7647	0.76	0.75
Zscore_PCC_RFE_7_LDA	0.8262 [0.7704-0.8820]	0.0285	0.8082	0.491	0.5394	0.7696	1.0294	0.7549	0.7843	0.7778	0.7619
Zscore_PCC_RFE_7_AE	0.767 [0.7011-0.8330]	0.0336	0.7313	0.5677	0.4511	0.7255	1.0196	0.7157	0.7353	0.73	0.7212
Zscore_PCC_RFE_7_RF	0.8281 [0.7741-0.8822]	0.0276	0.8366	0.445	0.5041	0.75	0.8725	0.8137	0.6863	0.7217	0.7865
Zscore_PCC_RFE_7_LR	0.8234 [0.7676-0.8793]	0.0285	0.8198	0.5481	0.5208	0.7598	1.0686	0.7255	0.7941	0.7789	0.7431
Zscore_PCC_RFE_7_LRLasso	0.8156 [0.7584-0.8727]	0.0292	0.7965	0.5318	0.5102	0.7549	1.0392	0.7353	0.7745	0.7653	0.7453
Zscore_PCC_RFE_7_AB	0.7866 [0.7245-0.8488]	0.0317	0.759	0.5076	0.4889	0.7402	1.1863	0.6471	0.8333	0.7952	0.7025
Zscore_PCC_RFE_7_DT	0.6863 [0.6223-0.7503]	0.0326	0.7647	1	0.3725	0.6863	1	0.6863	0.6863	0.6863	0.6863
Zscore_PCC_RFE_7_GP	0.8067 [0.7475-0.8659]	0.0302	0.8026	0.5155	0.5507	0.7745	1.0784	0.7353	0.8137	0.7979	0.7545
Zscore_PCC_RFE_7_NB	0.7838 [0.7217-0.8460]	0.0317	0.7869	0.9608	0.486	0.7255	1.3725	0.5392	0.9118	0.8594	0.6643
Zscore_PCC_RFE_8_SVM	0.8234 [0.7676-0.8793]	0.0285	0.8239	0.5856	0.5134	0.7549	1.1176	0.6961	0.8137	0.7889	0.7281
Zscore_PCC_RFE_8_LDA	0.8058 [0.7456-0.8661]	0.0307	0.7488	0.5496	0.5529	0.7745	1.1176	0.7157	0.8333	0.8111	0.7456
Zscore_PCC_RFE_8_AE	0.6648 [0.5884-0.7413]	0.039	0.5921	0.491	0.3693	0.6765	0.7059	0.8235	0.5294	0.6364	0.75
Zscore_PCC_RFE_8_RF	0.8133 [0.7566-0.8700]	0.0289	0.817	0.46	0.4926	0.7451	0.902	0.7941	0.6961	0.7232	0.7717
Zscore_PCC_RFE_8_LR	0.8175 [0.7605-0.8745]	0.0291	0.8119	0.5327	0.5114	0.7549	1.0784	0.7157	0.7941	0.7766	0.7364
Zscore_PCC_RFE_8_LRLasso	0.8081 [0.7494-0.8667]	0.0299	0.7871	0.5215	0.5006	0.75	1.049	0.7255	0.7745	0.7629	0.7383
Zscore_PCC_RFE_8_AB	0.7969 [0.7367-0.8570]	0.0307	0.788	0.508	0.4673	0.7304	1.1667	0.6471	0.8137	0.7765	0.6975
Zscore_PCC_RFE_8_DT	0.6667 [0.6018-0.7315]	0.0331	0.7527	1	0.3339	0.6667	0.9412	0.6961	0.6373	0.6574	0.6771
Zscore_PCC_RFE_8_GP	0.795 [0.7334-0.8565]	0.0314	0.784	0.5329	0.5424	0.7696	1.1078	0.7157	0.8235	0.8022	0.7434
Zscore_PCC_RFE_8_NB	0.7883 [0.7265-0.8500]	0.0315	0.7944	0.9354	0.4819	0.7353	1.2157	0.6275	0.8431	0.8	0.6935
Zscore_PCC_RFE_9_SVM	0.8224 [0.7665-0.8783]	0.0285	0.8212	0.4	0.5071	0.75	0.8333	0.8333	0.6667	0.7143	0.8

Zscore_PCC_RFE_9_LDA	0.8063 [0.7463-0.8663]	0.0306	0.7522	0.5488	0.532	0.7647	1.098	0.7157	0.8137	0.7935	0.7411
Zscore_PCC_RFE_9_AE	0.7049 [0.6321-0.7778]	0.0372	0.6855	0.4987	0.4327	0.7108	1.2255	0.598	0.8235	0.7722	0.672
Zscore_PCC_RFE_9_RF	0.8154 [0.7586-0.8722]	0.029	0.8182	0.53	0.5002	0.75	1.0294	0.7353	0.7647	0.7576	0.7429
Zscore_PCC_RFE_9_LR	0.8157 [0.7584-0.8731]	0.0292	0.8009	0.5999	0.5029	0.75	1.1078	0.6961	0.8039	0.7802	0.7257
Zscore_PCC_RFE_9_LRLasso	0.8073 [0.7485-0.8661]	0.03	0.7848	0.5518	0.4926	0.7451	1.098	0.6961	0.7941	0.7717	0.7232
Zscore_PCC_RFE_9_AB	0.7755 [0.7116-0.8393]	0.0326	0.7293	0.4994	0.451	0.7255	1	0.7255	0.7255	0.7255	0.7255
Zscore_PCC_RFE_9_DT	0.6765 [0.6120-0.7410]	0.0329	0.7567	1	0.353	0.6765	1.0196	0.6667	0.6863	0.68	0.6731
Zscore_PCC_RFE_9_GP	0.7911 [0.7291-0.8531]	0.0316	0.7875	0.5264	0.5331	0.7647	1.1176	0.7059	0.8235	0.8	0.7368
Zscore_PCC_RFE_9_NB	0.781 [0.7182-0.8439]	0.0321	0.7781	0.9705	0.5098	0.7451	1.2745	0.6078	0.8824	0.8378	0.6923
Zscore_PCC_RFE_10_SVM	0.8233 [0.7675-0.8790]	0.0284	0.8234	0.5965	0.5041	0.75	1.1275	0.6863	0.8137	0.7865	0.7217
Zscore_PCC_RFE_10_LDA	0.807 [0.7471-0.8669]	0.0306	0.7506	0.5315	0.5303	0.7647	1.0588	0.7353	0.7941	0.7812	0.75
Zscore_PCC_RFE_10_AE	0.758 [0.6920-0.8239]	0.0337	0.7255	0.5077	0.4118	0.7059	1	0.7059	0.7059	0.7059	0.7059
Zscore_PCC_RFE_10_RF	0.8147 [0.7572-0.8721]	0.0293	0.8084	0.535	0.5107	0.7549	1.0588	0.7255	0.7843	0.7708	0.7407
Zscore_PCC_RFE_10_LR	0.8128 [0.7549-0.8706]	0.0295	0.7968	0.5332	0.5012	0.75	1.0686	0.7157	0.7843	0.7684	0.7339
Zscore_PCC_RFE_10_LRLasso	0.8057 [0.7467-0.8648]	0.0301	0.7837	0.5317	0.491	0.7451	1.0588	0.7157	0.7745	0.7604	0.7315
Zscore_PCC_RFE_10_AB	0.7809 [0.7174-0.8444]	0.0324	0.74	0.4974	0.4936	0.7451	0.8824	0.8039	0.6863	0.7193	0.7778
Zscore_PCC_RFE_10_DT	0.6765 [0.6121-0.7409]	0.0329	0.7555	1	0.3536	0.6765	1.0588	0.6471	0.7059	0.6875	0.6667
Zscore_PCC_RFE_10_GP	0.776 [0.7126-0.8393]	0.0323	0.774	0.5085	0.4815	0.7402	1.0686	0.7059	0.7745	0.7579	0.7248
Zscore_PCC_RFE_10_NB	0.7827 [0.7200-0.8453]	0.032	0.7846	0.9755	0.5069	0.7451	1.2549	0.6176	0.8725	0.8289	0.6953
Zscore_PCC_RFE_11_SVM	0.8124 [0.7542-0.8706]	0.0297	0.7998	0.4648	0.5208	0.7598	0.9314	0.7941	0.7255	0.7431	0.7789
Zscore_PCC_RFE_11_LDA	0.8031 [0.7426-0.8635]	0.0308	0.7496	0.4948	0.5303	0.7647	1.0588	0.7353	0.7941	0.7812	0.75
Zscore_PCC_RFE_11_AE	0.7314 [0.6622-0.8007]	0.0354	0.7106	0.536	0.4035	0.6961	0.7647	0.8137	0.5784	0.6587	0.7564
Zscore_PCC_RFE_11_RF	0.8175 [0.7605-0.8745]	0.0291	0.809	0.525	0.4906	0.7451	1.0392	0.7255	0.7647	0.7551	0.7358
Zscore_PCC_RFE_11_LR	0.7976 [0.7359-0.8592]	0.0315	0.7367	0.5381	0.5012	0.75	1.0686	0.7157	0.7843	0.7684	0.7339
Zscore_PCC_RFE_11_LRLasso	0.7961 [0.7345-0.8578]	0.0315	0.739	0.5175	0.5	0.75	1.0098	0.7451	0.7549	0.7525	0.7476
Zscore_PCC_RFE_11_AB	0.8066 [0.7483-0.8649]	0.0297	0.8035	0.5254	0.4924	0.7353	1.2941	0.5882	0.8824	0.8333	0.6818
Zscore_PCC_RFE_11_DT	0.6569 [0.5917-0.7220]	0.0332	0.7389	1	0.3152	0.6569	1.098	0.6078	0.7059	0.6739	0.6429
Zscore_PCC_RFE_11_GP	0.7785 [0.7155-0.8414]	0.0321	0.7754	0.5038	0.4706	0.7353	1	0.7353	0.7353	0.7353	0.7353
Zscore_PCC_RFE_11_NB	0.7771 [0.7140-0.8402]	0.0322	0.7826	0.972	0.4765	0.7353	1.1569	0.6569	0.8137	0.7791	0.7034
Zscore_PCC_RFE_12_SVM	0.8145 [0.7564-0.8726]	0.0297	0.7889	0.4502	0.5102	0.7549	0.9608	0.7745	0.7353	0.7453	0.7653
Zscore_PCC_RFE_12_LDA	0.7983 [0.7372-0.8593]	0.0312	0.7362	0.5227	0.4926	0.7451	1.098	0.6961	0.7941	0.7717	0.7232
Zscore_PCC_RFE_12_AE	0.7714 [0.7069-0.8360]	0.0329	0.7596	0.5392	0.4511	0.7255	0.9804	0.7353	0.7157	0.7212	0.73
Zscore_PCC_RFE_12_RF	0.802 [0.7431-0.8610]	0.0301	0.8008	0.415	0.4528	0.7206	0.7745	0.8333	0.6078	0.68	0.7848
Zscore_PCC_RFE_12_LR	0.8013 [0.7403-0.8623]	0.0311	0.7385	0.4078	0.4823	0.7402	0.9118	0.7843	0.6961	0.7207	0.7634
Zscore_PCC_RFE_12_LRLasso	0.7962 [0.7347-0.8578]	0.0314	0.7324	0.4351	0.4815	0.7402	0.9314	0.7745	0.7059	0.7248	0.7579
Zscore_PCC_RFE_12_AB	0.7967 [0.7359-0.8575]	0.031	0.7577	0.5056	0.4804	0.7402	0.9902	0.7451	0.7353	0.7379	0.7426
Zscore_PCC_RFE_12_DT	0.6961 [0.6327-0.7595]	0.0324	0.7721	1	0.3922	0.6961	1	0.6961	0.6961	0.6961	0.6961
Zscore_PCC_RFE_12_GP	0.7743 [0.7106-0.8380]	0.0325	0.7587	0.5046	0.4815	0.7402	1.0686	0.7059	0.7745	0.7579	0.7248
Zscore_PCC_RFE_12_NB	0.7736 [0.7103-0.8370]	0.0323	0.784	0.9897	0.4551	0.7206	1.2451	0.598	0.8431	0.7922	0.6772
Zscore_PCC_RFE_13_SVM	0.8218 [0.7640-0.8796]	0.0295	0.7844	0.4028	0.5529	0.7745	0.8824	0.8333	0.7157	0.7456	0.8111
Zscore_PCC_RFE_13_LDA	0.8024 [0.7417-0.8630]	0.031	0.7432	0.3905	0.5002	0.75	0.9706	0.7647	0.7353	0.7429	0.7576
Zscore_PCC_RFE_13_AE	0.7339 [0.6653-0.8024]	0.035	0.6961	0.4656	0.3744	0.6863	0.902	0.7353	0.6373	0.6696	0.7065
Zscore_PCC_RFE_13_RF	0.8114 [0.7533-0.8696]	0.0297	0.8109	0.515	0.5198	0.7598	1.0294	0.7451	0.7745	0.7677	0.7524
Zscore_PCC_RFE_13_LR	0.8021 [0.7410-0.8632]	0.0312	0.7554	0.4383	0.5298	0.7647	0.9608	0.7843	0.7451	0.7547	0.7755
Zscore_PCC_RFE_13_LRLasso	0.7988 [0.7373-0.8604]	0.0314	0.7396	0.4084	0.5331	0.7647	0.8824	0.8235	0.7059	0.7368	0.8
Zscore_PCC_RFE_13_AB	0.7747 [0.7106-0.8388]	0.0327	0.7306	0.5025	0.4608	0.7304	0.9902	0.7353	0.7255	0.7282	0.7327
Zscore_PCC_RFE_13_DT	0.7206 [0.6588-0.7824]	0.0315	0.79	1	0.4417	0.7206	1.049	0.6961	0.7451	0.732	0.7103
Zscore_PCC_RFE_13_GP	0.7762 [0.7124-0.8401]	0.0326	0.7551	0.5038	0.5012	0.75	1.0686	0.7157	0.7843	0.7684	0.7339
Zscore_PCC_RFE_13_NB	0.7711 [0.7073-0.8350]	0.0326	0.7681	0.9744	0.4765	0.7353	1.1569	0.6569	0.8137	0.7791	0.7034
Zscore_PCC_RFE_14_SVM	0.8168 [0.7579-0.8757]	0.03	0.7715	0.4619	0.5295	0.7647	0.9804	0.7745	0.7549	0.7596	0.77
Zscore_PCC_RFE_14_LDA	0.7983 [0.7372-0.8595]	0.0312	0.7383	0.69	0.4819	0.7353	1.2157	0.6275	0.8431	0.8	0.6935
Zscore_PCC_RFE_14_AE	0.7265 [0.6579-0.7952]	0.035	0.7212	0.613	0.3546	0.6765	0.902	0.7255	0.6275	0.6607	0.6957
Zscore_PCC_RFE_14_RF	0.8247 [0.7689-0.8805]	0.0285	0.8114	0.47	0.5413	0.7696	0.9118	0.8137	0.7255	0.7477	0.7957
Zscore_PCC_RFE_14_LR	0.8068 [0.7465-0.8672]	0.0308	0.7682	0.419	0.531	0.7647	0.9216	0.8039	0.7255	0.7455	0.7872
Zscore_PCC_RFE_14_LRLasso	0.7995 [0.7380-0.8610]	0.0314	0.7449	0.4087	0.5331	0.7647	0.8824	0.8235	0.7059	0.7368	0.8
Zscore_PCC_RFE_14_AB	0.7716 [0.7077-0.8354]	0.0326	0.7463	0.4989	0.4355	0.7157	0.8627	0.7843	0.6471	0.6897	0.75
Zscore_PCC_RFE_14_DT	0.7108 [0.6483-0.7733]	0.0319	0.7826	1	0.4218	0.7108	1.0294	0.6961	0.7255	0.7172	0.7048
Zscore_PCC_RFE_14_GP	0.7784 [0.7145-0.8422]	0.0326	0.7522	0.5215	0.5089	0.75	1.1863	0.6569	0.8431	0.8072	0.7107
Zscore_PCC_RFE_14_NB	0.7681 [0.7038-0.8324]	0.0328	0.7724	0.9788	0.4553	0.7255	1.1373	0.6569	0.7941	0.7614	0.6983
Zscore_PCC_RFE_15_SVM	0.7897 [0.7270-0.8525]	0.032	0.7221	0.4457	0.4815	0.7402	0.9314	0.7745	0.7059	0.7248	0.7579
Zscore_PCC_RFE_15_LDA	0.7897 [0.7275-0.8518]	0.0317	0.7332	0.6074	0.4843	0.7402	1.1275	0.6765	0.8039	0.7753	0.713
Zscore_PCC_RFE_15_AE	0.7809 [0.7174-0.8443]	0.0324	0.759	0.5288	0.4608	0.7304	1.0098	0.7255	0.7353	0.7327	0.7282
Zscore_PCC_RFE_15_RF	0.8169 [0.7603-0.8735]	0.0289	0.8185	0.505	0.5002	0.75	1.0294	0.7353	0.7647	0.7576	0.7429
Zscore_PCC_RFE_15_LR	0.7926 [0.7302-0.8550]	0.0318	0.7307	0.3974	0.4857	0.7402	0.8529	0.8137	0.6667	0.7094	0.7816
Zscore_PCC_RFE_15_LRLasso	0.7947 [0.7329-0.8565]	0.0315	0.7458	0.381	0.502	0.7451	0.7843	0.8529	0.6373	0.7016	0.8125
Zscore_PCC_RFE_15_AB	0.7674 [0.7029-0.8319]	0.0329	0.7337	0.4981	0.4417	0.7206	0.951	0.7451	0.6961	0.7103	0.732
Zscore_PCC_RFE_15_DT	0.7059 [0.6431-0.7687]	0.0321	0.7799	1	0.4118	0.7059	0.9804	0.7157	0.6961	0.7019	0.71
Zscore_PCC_RFE_15_GP	0.7716 [0.7071-0.8361]	0.0329	0.7722	0.5022	0.491	0.7451	1.0588	0.7157	0.7745	0.7604	0.7315
Zscore_PCC_RFE_15_NB	0.7724 [0.7089-0.8359]	0.0324	0.786	0.9868	0.469	0.7304	1.1863	0.6373	0.8235	0.7831	0.6942

Zscore_PCC_RFE_16_SVM	0.7954 [0.7330-0.8577]	0.0318	0.7255	0.4263	0.5029	0.75	0.8922	0.8039	0.6961	0.7257	0.7802
Zscore_PCC_RFE_16_LDA	0.7921 [0.7301-0.8541]	0.0316	0.7353	0.5882	0.4949	0.7451	1.1373	0.6765	0.8137	0.7841	0.7155
Zscore_PCC_RFE_16_AE	0.776 [0.7119-0.8402]	0.0327	0.7458	0.4762	0.4613	0.7304	0.951	0.7549	0.7059	0.7196	0.7423
Zscore_PCC_RFE_16_RF	0.8124 [0.7551-0.8698]	0.0293	0.8137	0.545	0.4751	0.7353	1.1373	0.6667	0.8039	0.7727	0.7069
Zscore_PCC_RFE_16_LR	0.7891 [0.7257-0.8525]	0.0323	0.7142	0.3875	0.498	0.7451	0.8235	0.8333	0.6569	0.7083	0.7976
Zscore_PCC_RFE_16_LRLasso	0.7945 [0.7320-0.8570]	0.0319	0.7238	0.4777	0.4903	0.7451	0.9804	0.7549	0.7353	0.7404	0.75
Zscore_PCC_RFE_16_AB	0.7596 [0.6940-0.8252]	0.0335	0.7339	0.5203	0.4425	0.7108	1.3039	0.5588	0.8627	0.8028	0.6617
Zscore_PCC_RFE_16_DT	0.7108 [0.6483-0.7733]	0.0319	0.7829	1	0.4216	0.7108	1.0098	0.7059	0.7157	0.7129	0.7087
Zscore_PCC_RFE_16_GP	0.7801 [0.7167-0.8435]	0.0323	0.7802	0.5003	0.5102	0.7549	1.0392	0.7353	0.7745	0.7653	0.7453
Zscore_PCC_RFE_16_NB	0.7588 [0.6931-0.8246]	0.0336	0.7286	0.9841	0.4566	0.7255	1.1569	0.6471	0.8039	0.7674	0.6949
Zscore_PCC_RFE_17_SVM	0.7967 [0.7348-0.8586]	0.0316	0.7327	0.521	0.481	0.7402	1.049	0.7157	0.7647	0.7526	0.729
Zscore_PCC_RFE_17_LDA	0.7911 [0.7293-0.8530]	0.0315	0.7365	0.5808	0.4751	0.7353	1.1373	0.6667	0.8039	0.7727	0.7069
Zscore_PCC_RFE_17_AE	0.7549 [0.6878-0.8220]	0.0342	0.7151	0.5628	0.4321	0.7157	1.0588	0.6863	0.7451	0.7292	0.7037
Zscore_PCC_RFE_17_RF	0.8079 [0.7490-0.8669]	0.0301	0.8062	0.575	0.5162	0.7549	1.1569	0.6765	0.8333	0.8023	0.7203
Zscore_PCC_RFE_17_LR	0.7861 [0.7226-0.8497]	0.0324	0.714	0.3555	0.4842	0.7353	0.7647	0.8529	0.6176	0.6905	0.8077
Zscore_PCC_RFE_17_LRLasso	0.797 [0.7350-0.8590]	0.0316	0.7273	0.4064	0.498	0.7451	0.8235	0.8333	0.6569	0.7083	0.7976
Zscore_PCC_RFE_17_AB	0.7442 [0.6763-0.8122]	0.0347	0.6957	0.5171	0.4183	0.7059	1.1765	0.6176	0.7941	0.75	0.675
Zscore_PCC_RFE_17_DT	0.7108 [0.6484-0.7732]	0.0319	0.7843	1	0.4221	0.7108	0.951	0.7353	0.6863	0.7009	0.7216
Zscore_PCC_RFE_17_GP	0.7861 [0.7230-0.8493]	0.0322	0.7875	0.5115	0.5333	0.7598	1.2255	0.6471	0.8725	0.8354	0.712
Zscore_PCC_RFE_17_NB	0.7515 [0.6847-0.8183]	0.0341	0.7129	0.9838	0.4344	0.7157	1.1176	0.6569	0.7745	0.7444	0.693
Zscore_PCC_RFE_18_SVM	0.7858 [0.7226-0.8489]	0.0322	0.7258	0.4546	0.4635	0.7304	0.8922	0.7843	0.6765	0.708	0.7582
Zscore_PCC_RFE_18_LDA	0.782 [0.7190-0.8450]	0.0321	0.7329	0.5822	0.4626	0.7304	1.0882	0.6863	0.7745	0.7527	0.7117
Zscore_PCC_RFE_18_AE	0.7642 [0.6995-0.8289]	0.033	0.7695	0.5756	0.4258	0.7059	1.2549	0.5784	0.8333	0.7763	0.6641
Zscore_PCC_RFE_18_RF	0.8061 [0.7476-0.8646]	0.0298	0.8078	0.61	0.4781	0.7353	1.1765	0.6471	0.8235	0.7857	0.7
Zscore_PCC_RFE_18_LR	0.7737 [0.7091-0.8384]	0.033	0.7054	0.5597	0.4422	0.7206	1.0686	0.6863	0.7549	0.7368	0.7064
Zscore_PCC_RFE_18_LRLasso	0.7824 [0.7189-0.8459]	0.0324	0.7166	0.4385	0.4438	0.7206	0.8922	0.7745	0.6667	0.6991	0.7473
Zscore_PCC_RFE_18_AB	0.7743 [0.7100-0.8386]	0.0328	0.7315	0.503	0.4417	0.7206	0.951	0.7451	0.6961	0.7103	0.732
Zscore_PCC_RFE_18_DT	0.652 [0.5863-0.7176]	0.0335	0.7405	1	0.3041	0.652	0.9706	0.6667	0.6373	0.6476	0.6566
Zscore_PCC_RFE_18_GP	0.7954 [0.7340-0.8568]	0.0313	0.7933	0.5047	0.5179	0.7549	1.1765	0.6667	0.8431	0.8095	0.7167
Zscore_PCC_RFE_18_NB	0.7469 [0.6797-0.8142]	0.0343	0.7081	0.9931	0.4291	0.7108	1.1863	0.6176	0.8039	0.759	0.6777
Zscore_PCC_RFE_19_SVM	0.7779 [0.7131-0.8426]	0.033	0.7163	0.5784	0.4673	0.7304	1.1667	0.6471	0.8137	0.7765	0.6975
Zscore_PCC_RFE_19_LDA	0.7848 [0.7218-0.8478]	0.0321	0.7335	0.5925	0.4949	0.7451	1.1373	0.6765	0.8137	0.7841	0.7155
Zscore_PCC_RFE_19_AE	0.752 [0.6850-0.8191]	0.0342	0.6993	0.4599	0.4317	0.7157	0.9608	0.7353	0.6961	0.7075	0.7245
Zscore_PCC_RFE_19_RF	0.818 [0.7613-0.8747]	0.0289	0.8252	0.59	0.4889	0.7402	1.1863	0.6471	0.8333	0.7952	0.7025
Zscore_PCC_RFE_19_LR	0.7777 [0.7135-0.8418]	0.0327	0.7113	0.6744	0.4528	0.7206	1.2255	0.6078	0.8333	0.7848	0.68
Zscore_PCC_RFE_19_LRLasso	0.7859 [0.7226-0.8491]	0.0322	0.7202	0.4242	0.4541	0.7255	0.8824	0.7843	0.6667	0.7018	0.7556
Zscore_PCC_RFE_19_AB	0.7705 [0.7055-0.8354]	0.0331	0.7202	0.5152	0.4658	0.7304	1.1471	0.6569	0.8039	0.7701	0.7009
Zscore_PCC_RFE_19_DT	0.6422 [0.5761-0.7082]	0.0337	0.7344	1	0.2847	0.6422	0.951	0.6667	0.6176	0.6355	0.6495
Zscore_PCC_RFE_19_GP	0.8069 [0.7475-0.8663]	0.0303	0.8138	0.5017	0.5303	0.7647	1.0588	0.7353	0.7941	0.7812	0.75
Zscore_PCC_RFE_19_NB	0.7409 [0.6729-0.8088]	0.0347	0.7001	0.9963	0.4108	0.701	1.2059	0.598	0.8039	0.7531	0.6667
Zscore_PCC_RFE_20_SVM	0.8006 [0.7391-0.8621]	0.0314	0.7345	0.4778	0.5006	0.75	0.951	0.7745	0.7255	0.7383	0.7629
Zscore_PCC_RFE_20_LDA	0.7959 [0.7343-0.8576]	0.0315	0.7437	0.5722	0.4926	0.7451	1.098	0.6961	0.7941	0.7717	0.7232
Zscore_PCC_RFE_20_AE	0.7315 [0.6635-0.7996]	0.0347	0.697	0.587	0.3834	0.6814	1.3235	0.5196	0.8431	0.7681	0.637
Zscore_PCC_RFE_20_RF	0.8221 [0.7663-0.8779]	0.0285	0.8306	0.555	0.5147	0.7549	1.1373	0.6863	0.8235	0.7955	0.7241
Zscore_PCC_RFE_20_LR	0.7814 [0.7179-0.8450]	0.0324	0.7147	0.653	0.4508	0.7206	1.2059	0.6176	0.8235	0.7778	0.6829
Zscore_PCC_RFE_20_LRLasso	0.7934 [0.7315-0.8554]	0.0316	0.7286	0.4196	0.4872	0.7402	0.8333	0.8235	0.6569	0.7059	0.7882
Zscore_PCC_RFE_20_AB	0.7666 [0.7023-0.8310]	0.0328	0.7376	0.5054	0.3925	0.6961	1.0392	0.6765	0.7157	0.7041	0.6887
Zscore_PCC_RFE_20_DT	0.6176 [0.5507-0.6846]	0.0342	0.7107	1	0.2355	0.6176	1.0392	0.598	0.6373	0.6224	0.6132
Zscore_PCC_RFE_20_GP	0.8024 [0.7421-0.8627]	0.0308	0.8015	0.5017	0.5303	0.7647	1.0588	0.7353	0.7941	0.7812	0.75
Zscore_PCC_RFE_20_NB	0.7425 [0.6749-0.8101]	0.0345	0.7035	0.9974	0.4035	0.6961	1.2353	0.5784	0.8137	0.7564	0.6587
Zscore_PCC_Relief_1_SVM	0.5764 [0.4977-0.6551]	0.0402	0.5861	0.438	0.2321	0.5833	0.3039	0.9314	0.2353	0.5491	0.7742
Zscore_PCC_Relief_1_LDA	0.5639 [0.4849-0.6429]	0.0403	0.5741	0.4258	0.2739	0.5882	0.2353	0.9706	0.2059	0.55	0.875
Zscore_PCC_Relief_1_AE	0.4996 [0.4190-0.5802]	0.0411	0.5404	0.5398	0.1511	0.5588	1.6275	0.2451	0.8725	0.6579	0.5361
Zscore_PCC_Relief_1_RF	0.6287 [0.5524-0.7051]	0.039	0.6349	0.325	0.2581	0.6275	0.8431	0.7059	0.549	0.6102	0.6512
Zscore_PCC_Relief_1_LR	0.584 [0.5058-0.6622]	0.0399	0.5828	0.5316	0.1569	0.5784	1.0196	0.5686	0.5882	0.58	0.5769
Zscore_PCC_Relief_1_LRLasso	0.5811 [0.5029-0.6594]	0.0399	0.585	0.5349	0.1569	0.5784	1.0196	0.5686	0.5882	0.58	0.5769
Zscore_PCC_Relief_1_AB	0.6495 [0.5744-0.7245]	0.0383	0.6082	0.4999	0.2353	0.6176	0.9804	0.6275	0.6078	0.6154	0.62
Zscore_PCC_Relief_1_DT	0.5882 [0.5204-0.6561]	0.0346	0.6896	1	0.1765	0.5882	1.0196	0.5784	0.598	0.59	0.5865
Zscore_PCC_Relief_1_GP	0.5788 [0.5004-0.6572]	0.04	0.5979	0.5015	0.1936	0.5882	0.5882	0.7941	0.3824	0.5625	0.65
Zscore_PCC_Relief_1_NB	0.5566 [0.4769-0.6363]	0.0407	0.5529	0.6356	0.1544	0.5735	0.6961	0.7255	0.4216	0.5564	0.6056
Zscore_PCC_Relief_2_SVM	0.6616 [0.5875-0.7357]	0.0378	0.6613	0.5253	0.255	0.6275	1.0196	0.6176	0.6373	0.63	0.625
Zscore_PCC_Relief_2_LDA	0.6323 [0.5563-0.7082]	0.0387	0.6365	0.5822	0.2856	0.6275	1.451	0.402	0.8529	0.7321	0.5878
Zscore_PCC_Relief_2_AE	0.5422 [0.4621-0.6223]	0.0409	0.5843	0.5009	0.2311	0.598	1.5294	0.3333	0.8627	0.7083	0.5641
Zscore_PCC_Relief_2_RF	0.726 [0.6565-0.7955]	0.0355	0.6905	0.63	0.3815	0.6863	1.2157	0.5784	0.7941	0.7375	0.6532
Zscore_PCC_Relief_2_LR	0.671 [0.5973-0.7447]	0.0376	0.6743	0.5334	0.2847	0.6422	1.049	0.6176	0.6667	0.6495	0.6355
Zscore_PCC_Relief_2_LRLasso	0.6729 [0.5994-0.7464]	0.0375	0.6721	0.562	0.2894	0.6422	1.1863	0.549	0.7353	0.6747	0.6198
Zscore_PCC_Relief_2_AB	0.6856 [0.6114-0.7599]	0.0379	0.6129	0.5003	0.3737	0.6863	0.9216	0.7255	0.6471	0.6727	0.7021
Zscore_PCC_Relief_2_DT	0.6471 [0.5815-0.7126]	0.0334	0.741	1	0.2955	0.6471	0.902	0.6961	0.598	0.6339	0.663
Zscore_PCC_Relief_2_GP	0.6595 [0.5847-0.7342]	0.0381	0.6517	0.5092	0.3039	0.652	1.0098	0.6471	0.6569	0.6535	0.6505

Zscore_PCC_Relief_2_NB	0.5815 [0.5032-0.6598]	0.04	0.6104	0.9697	0.2157	0.5784	1.6863	0.2353	0.9216	0.75	0.5465
Zscore_PCC_Relief_3_SVM	0.736 [0.6669-0.8051]	0.0353	0.7277	0.5543	0.4383	0.7157	1.1765	0.6275	0.8039	0.7619	0.6833
Zscore_PCC_Relief_3_LDA	0.7345 [0.6651-0.8039]	0.0354	0.7103	0.5747	0.4551	0.7206	1.2451	0.598	0.8431	0.7922	0.6772
Zscore_PCC_Relief_3_AE	0.5312 [0.4514-0.6111]	0.0408	0.5437	0.5065	0.1455	0.5588	1.5882	0.2647	0.8529	0.6429	0.537
Zscore_PCC_Relief_3_RF	0.759 [0.6940-0.8240]	0.0332	0.7619	0.625	0.4327	0.7108	1.2255	0.598	0.8235	0.7722	0.672
Zscore_PCC_Relief_3_LR	0.7436 [0.6752-0.8119]	0.0349	0.7444	0.5758	0.4508	0.7206	1.2059	0.6176	0.8235	0.7778	0.6829
Zscore_PCC_Relief_3_LRLasso	0.7455 [0.6773-0.8137]	0.0348	0.7436	0.5613	0.446	0.7206	1.1471	0.6471	0.7941	0.7586	0.6923
Zscore_PCC_Relief_3_AB	0.7383 [0.6699-0.8067]	0.0349	0.7026	0.5004	0.3922	0.6961	1	0.6961	0.6961	0.6961	0.6961
Zscore_PCC_Relief_3_DT	0.6422 [0.5766-0.7077]	0.0334	0.7395	1	0.2867	0.6422	0.8725	0.7059	0.5784	0.6261	0.6629
Zscore_PCC_Relief_3_GP	0.7373 [0.6683-0.8063]	0.0352	0.7158	0.5261	0.4417	0.7206	1.049	0.6961	0.7451	0.732	0.7103
Zscore_PCC_Relief_3_NB	0.6077 [0.5305-0.6850]	0.0394	0.5723	0.9552	0.1985	0.598	0.8431	0.6765	0.5196	0.5847	0.6163
Zscore_PCC_Relief_4_SVM	0.7203 [0.6511-0.7894]	0.0353	0.7137	0.5791	0.3554	0.6765	1.1176	0.6176	0.7353	0.7	0.6579
Zscore_PCC_Relief_4_LDA	0.695 [0.6231-0.7669]	0.0367	0.6667	0.5626	0.3339	0.6667	1.0588	0.6373	0.6961	0.6771	0.6574
Zscore_PCC_Relief_4_AE	0.6837 [0.6105-0.7569]	0.0374	0.6254	0.4751	0.3106	0.652	0.7941	0.7549	0.549	0.626	0.6914
Zscore_PCC_Relief_4_RF	0.7521 [0.6852-0.8190]	0.0341	0.722	0.42	0.4368	0.7157	0.8431	0.7941	0.6373	0.6864	0.7558
Zscore_PCC_Relief_4_LR	0.722 [0.6531-0.7909]	0.0352	0.7177	0.623	0.3492	0.6716	1.1863	0.5784	0.7647	0.7108	0.6446
Zscore_PCC_Relief_4_LRLasso	0.7297 [0.6615-0.7979]	0.0348	0.7286	0.5898	0.3657	0.6814	1.1275	0.6176	0.7451	0.7079	0.6609
Zscore_PCC_Relief_4_AB	0.7116 [0.6399-0.7834]	0.0366	0.6646	0.5079	0.4126	0.701	1.2255	0.5882	0.8137	0.7595	0.664
Zscore_PCC_Relief_4_DT	0.6176 [0.5506-0.6847]	0.0342	0.7132	1	0.2353	0.6176	1	0.6176	0.6176	0.6176	0.6176
Zscore_PCC_Relief_4_GP	0.7122 [0.6405-0.7839]	0.0366	0.7217	0.5721	0.4383	0.7157	1.1765	0.6275	0.8039	0.7619	0.6833
Zscore_PCC_Relief_4_NB	0.6149 [0.5379-0.6918]	0.0393	0.5829	0.8768	0.2111	0.5882	0.451	0.8627	0.3137	0.557	0.6957
Zscore_PCC_Relief_5_SVM	0.7585 [0.6935-0.8235]	0.0332	0.7473	0.4408	0.3971	0.6961	0.8431	0.7745	0.6176	0.6695	0.7326
Zscore_PCC_Relief_5_LDA	0.7203 [0.6507-0.7899]	0.0355	0.6785	0.6045	0.3631	0.6765	1.2353	0.5588	0.7941	0.7308	0.6429
Zscore_PCC_Relief_5_AE	0.6509 [0.5761-0.7257]	0.0382	0.6763	0.5186	0.3018	0.6324	1.4804	0.3922	0.8725	0.7547	0.5894
Zscore_PCC_Relief_5_RF	0.7667 [0.7015-0.8318]	0.0332	0.7474	0.485	0.4511	0.7255	0.9804	0.7353	0.7157	0.7212	0.73
Zscore_PCC_Relief_5_LR	0.7565 [0.6913-0.8218]	0.0333	0.7361	0.4329	0.3772	0.6863	0.8431	0.7647	0.6078	0.661	0.7209
Zscore_PCC_Relief_5_LRLasso	0.7561 [0.6907-0.8214]	0.0333	0.74	0.6211	0.3799	0.6863	1.1961	0.5882	0.7843	0.7317	0.6557
Zscore_PCC_Relief_5_AB	0.7262 [0.6564-0.7960]	0.0356	0.7011	0.5096	0.4146	0.701	1.2451	0.5784	0.8235	0.7662	0.6614
Zscore_PCC_Relief_5_DT	0.6471 [0.5812-0.7129]	0.0336	0.7334	1	0.2943	0.6471	1.0392	0.6275	0.6667	0.6531	0.6415
Zscore_PCC_Relief_5_GP	0.7401 [0.6721-0.8081]	0.0347	0.7394	0.5444	0.4541	0.7255	1.1176	0.6667	0.7843	0.7556	0.7018
Zscore_PCC_Relief_5_NB	0.6115 [0.5344-0.6886]	0.0394	0.5723	0.9592	0.2	0.5882	0.5294	0.8235	0.3529	0.56	0.6667
Zscore_PCC_Relief_6_SVM	0.7921 [0.7303-0.8539]	0.0315	0.7333	0.4457	0.4872	0.7402	0.8333	0.8235	0.6569	0.7059	0.7882
Zscore_PCC_Relief_6_LDA	0.7657 [0.7007-0.8307]	0.0332	0.7152	0.6007	0.4438	0.7157	1.2353	0.598	0.8333	0.7821	0.6746
Zscore_PCC_Relief_6_AE	0.7156 [0.6460-0.7852]	0.0355	0.7574	0.5343	0.3624	0.6667	1.3922	0.4706	0.8627	0.7742	0.6197
Zscore_PCC_Relief_6_RF	0.7901 [0.7282-0.8519]	0.0316	0.7708	0.435	0.4926	0.7451	0.902	0.7941	0.6961	0.7232	0.7717
Zscore_PCC_Relief_6_LR	0.7911 [0.7300-0.8523]	0.0312	0.7489	0.4252	0.4566	0.7255	0.8431	0.8039	0.6471	0.6949	0.7674
Zscore_PCC_Relief_6_LRLasso	0.7926 [0.7314-0.8538]	0.0312	0.7497	0.3937	0.4867	0.7353	0.7451	0.8627	0.6078	0.6875	0.8158
Zscore_PCC_Relief_6_AB	0.7424 [0.6735-0.8113]	0.0352	0.6892	0.4952	0.4383	0.7157	0.8235	0.8039	0.6275	0.6833	0.7619
Zscore_PCC_Relief_6_DT	0.6078 [0.5406-0.6750]	0.0343	0.7019	1	0.2161	0.6078	1.0588	0.5784	0.6373	0.6146	0.6019
Zscore_PCC_Relief_6_GP	0.7629 [0.6975-0.8283]	0.0334	0.7629	0.5778	0.4619	0.7255	1.2157	0.6176	0.8333	0.7875	0.6855
Zscore_PCC_Relief_6_NB	0.6138 [0.5366-0.6910]	0.0394	0.5662	0.9662	0.2762	0.6225	0.5392	0.8529	0.3922	0.5839	0.7273
Zscore_PCC_Relief_7_SVM	0.7918 [0.7291-0.8544]	0.032	0.7245	0.4505	0.4926	0.7451	0.902	0.7941	0.6961	0.7232	0.7717
Zscore_PCC_Relief_7_LDA	0.7306 [0.6595-0.8016]	0.0363	0.668	0.5691	0.4823	0.7402	1.0882	0.6961	0.7843	0.7634	0.7207
Zscore_PCC_Relief_7_AE	0.5986 [0.5211-0.6762]	0.0396	0.5977	0.5998	0.1977	0.5882	1.451	0.3627	0.8137	0.6607	0.5608
Zscore_PCC_Relief_7_RF	0.7837 [0.7205-0.8470]	0.0323	0.7654	0.395	0.5044	0.7451	0.7647	0.8627	0.6275	0.6984	0.8205
Zscore_PCC_Relief_7_LR	0.7804 [0.7171-0.8436]	0.0323	0.7329	0.5088	0.4706	0.7353	1	0.7353	0.7353	0.7353	0.7353
Zscore_PCC_Relief_7_LRLasso	0.7863 [0.7237-0.8490]	0.032	0.7314	0.5357	0.4714	0.7353	1.0588	0.7059	0.7647	0.75	0.7222
Zscore_PCC_Relief_7_AB	0.7454 [0.6770-0.8138]	0.0349	0.6933	0.508	0.4368	0.7157	1.1569	0.6373	0.7941	0.7558	0.6864
Zscore_PCC_Relief_7_DT	0.6471 [0.5812-0.7129]	0.0336	0.7334	1	0.2943	0.6471	1.0392	0.6275	0.6667	0.6531	0.6415
Zscore_PCC_Relief_7_GP	0.7739 [0.7086-0.8392]	0.0333	0.7496	0.5083	0.4806	0.7402	1.0294	0.7255	0.7549	0.7475	0.7333
Zscore_PCC_Relief_7_NB	0.6148 [0.5376-0.6919]	0.0393	0.5659	0.9844	0.2345	0.6078	0.6078	0.8039	0.4118	0.5775	0.6774
Zscore_PCC_Relief_8_SVM	0.8032 [0.7421-0.8644]	0.0312	0.7353	0.424	0.5345	0.7647	0.8627	0.8333	0.6961	0.7328	0.8068
Zscore_PCC_Relief_8_LDA	0.7575 [0.6901-0.8249]	0.0344	0.7	0.5414	0.4613	0.7304	1.049	0.7059	0.7549	0.7423	0.7196
Zscore_PCC_Relief_8_AE	0.4936 [0.4137-0.5734]	0.0407	0.4734	0.5354	0.0624	0.5294	1.3333	0.3627	0.6961	0.5441	0.5221
Zscore_PCC_Relief_8_RF	0.794 [0.7324-0.8557]	0.0315	0.7667	0.455	0.5239	0.7598	0.8725	0.8235	0.6961	0.7304	0.7978
Zscore_PCC_Relief_8_LR	0.7902 [0.7283-0.8521]	0.0316	0.739	0.601	0.4819	0.7353	1.2157	0.6275	0.8431	0.8	0.6935
Zscore_PCC_Relief_8_LRLasso	0.7947 [0.7329-0.8565]	0.0315	0.7397	0.4655	0.481	0.7402	0.951	0.7647	0.7157	0.729	0.7526
Zscore_PCC_Relief_8_AB	0.7441 [0.6774-0.8109]	0.034	0.7259	0.4945	0.3761	0.6863	0.8627	0.7549	0.6176	0.6638	0.7159
Zscore_PCC_Relief_8_DT	0.6225 [0.5565-0.6886]	0.0337	0.7091	1	0.2478	0.6225	1.1471	0.549	0.6961	0.6437	0.6068
Zscore_PCC_Relief_8_GP	0.7823 [0.7180-0.8466]	0.0328	0.755	0.5028	0.5107	0.7549	1.0588	0.7255	0.7843	0.7708	0.7407
Zscore_PCC_Relief_8_NB	0.6175 [0.5403-0.6946]	0.0394	0.5635	0.9954	0.2352	0.6127	0.7157	0.7549	0.4706	0.5878	0.6575
Zscore_PCC_Relief_9_SVM	0.7921 [0.7292-0.8550]	0.0321	0.7117	0.428	0.5041	0.75	0.8725	0.8137	0.6863	0.7217	0.7865
Zscore_PCC_Relief_9_LDA	0.7334 [0.6642-0.8026]	0.0353	0.669	0.471	0.3928	0.6961	0.9412	0.7255	0.6667	0.6852	0.7083
Zscore_PCC_Relief_9_AE	0.64 [0.5646-0.7154]	0.0385	0.6387	0.4974	0.2471	0.6225	0.8725	0.6863	0.5588	0.6087	0.6404
Zscore_PCC_Relief_9_RF	0.7704 [0.7055-0.8354]	0.0331	0.7454	0.545	0.471	0.7353	1.0392	0.7157	0.7549	0.7449	0.7264
Zscore_PCC_Relief_9_LR	0.775 [0.7108-0.8392]	0.0328	0.7106	0.48	0.4511	0.7255	0.9804	0.7353	0.7157	0.7212	0.73
Zscore_PCC_Relief_9_LRLasso	0.7787 [0.7146-0.8429]	0.0327	0.711	0.4125	0.4765	0.7353	0.8431	0.8137	0.6569	0.7034	0.7791
Zscore_PCC_Relief_9_AB	0.7373 [0.6687-0.8059]	0.035	0.6647	0.4934	0.3892	0.6912	0.8137	0.7843	0.598	0.6612	0.7349
Zscore_PCC_Relief_9_DT	0.652 [0.5867-0.7172]	0.0333	0.7347	1	0.3057	0.652	1.1078	0.598	0.7059	0.6703	0.6372

Zscore_PCC_Relief_9_GP	0.7637 [0.6980-0.8295]	0.0335	0.7459	0.5259	0.4832	0.7402	1.1078	0.6863	0.7941	0.7692	0.7168
Zscore_PCC_Relief_9_NB	0.594 [0.5154-0.6727]	0.0401	0.534	0.9964	0.2535	0.6176	0.6275	0.8039	0.4314	0.5857	0.6875
Zscore_PCC_Relief_10_SVM	0.7982 [0.7352-0.8611]	0.0321	0.7086	0.4564	0.5208	0.7598	0.9314	0.7941	0.7255	0.7431	0.7789
Zscore_PCC_Relief_10_LDA	0.7421 [0.6730-0.8113]	0.0353	0.6642	0.5424	0.4221	0.7108	1.049	0.6863	0.7353	0.7216	0.7009
Zscore_PCC_Relief_10_AE	0.6469 [0.5717-0.7220]	0.0383	0.6178	0.5607	0.27	0.6176	1.4902	0.3725	0.8627	0.7308	0.5789
Zscore_PCC_Relief_10_RF	0.7913 [0.7294-0.8532]	0.0316	0.7655	0.46	0.4926	0.7451	0.902	0.7941	0.6961	0.7232	0.7717
Zscore_PCC_Relief_10_LR	0.7802 [0.7163-0.8441]	0.0326	0.7123	0.4582	0.491	0.7451	0.9412	0.7745	0.7157	0.7315	0.7604
Zscore_PCC_Relief_10_LRLasso	0.7918 [0.7294-0.8542]	0.0319	0.7206	0.5917	0.4739	0.7353	1.1176	0.6765	0.7941	0.7667	0.7105
Zscore_PCC_Relief_10_AB	0.7582 [0.6905-0.8259]	0.0345	0.6684	0.4913	0.4857	0.7402	0.8529	0.8137	0.6667	0.7094	0.7816
Zscore_PCC_Relief_10_DT	0.6618 [0.5971-0.7264]	0.033	0.7422	1	0.3262	0.6618	1.1275	0.598	0.7255	0.6854	0.6435
Zscore_PCC_Relief_10_GP	0.7738 [0.7091-0.8386]	0.033	0.7464	0.5046	0.481	0.7402	1.049	0.7157	0.7647	0.7526	0.729
Zscore_PCC_Relief_10_NB	0.5852 [0.5067-0.6636]	0.04	0.5428	0.9977	0.1804	0.5833	0.6176	0.7745	0.3922	0.5603	0.6349
Zscore_PCC_Relief_11_SVM	0.7906 [0.7264-0.8547]	0.0327	0.6982	0.4209	0.5179	0.7549	0.8235	0.8431	0.6667	0.7167	0.8095
Zscore_PCC_Relief_11_LDA	0.7259 [0.6546-0.7972]	0.0364	0.635	0.5601	0.4125	0.7059	1.0588	0.6765	0.7353	0.7188	0.6944
Zscore_PCC_Relief_11_AE	0.6817 [0.6092-0.7541]	0.0369	0.6832	0.5859	0.2747	0.6373	1.0392	0.6176	0.6569	0.6429	0.6321
Zscore_PCC_Relief_11_RF	0.8069 [0.7465-0.8673]	0.0308	0.7696	0.435	0.5424	0.7696	0.8922	0.8235	0.7157	0.7434	0.8022
Zscore_PCC_Relief_11_LR	0.7731 [0.7080-0.8381]	0.0332	0.699	0.4101	0.4889	0.7402	0.8137	0.8333	0.6471	0.7025	0.7952
Zscore_PCC_Relief_11_LRLasso	0.7906 [0.7280-0.8531]	0.0319	0.7165	0.3929	0.4999	0.7451	0.8039	0.8431	0.6471	0.7049	0.8049
Zscore_PCC_Relief_11_AB	0.7574 [0.6905-0.8243]	0.0341	0.7068	0.5003	0.4412	0.7206	1.0098	0.7157	0.7255	0.7228	0.7184
Zscore_PCC_Relief_11_DT	0.6716 [0.6072-0.7359]	0.0328	0.7506	1	0.3452	0.6716	1.1078	0.6176	0.7255	0.6923	0.6549
Zscore_PCC_Relief_11_GP	0.7762 [0.7110-0.8415]	0.0333	0.7283	0.5192	0.5147	0.7549	1.1373	0.6863	0.8235	0.7955	0.7241
Zscore_PCC_Relief_11_NB	0.5641 [0.4846-0.6436]	0.0406	0.5186	0.9989	0.2192	0.6029	0.6569	0.7745	0.4314	0.5766	0.6567
Zscore_PCC_Relief_12_SVM	0.7711 [0.7049-0.8374]	0.0338	0.6835	0.446	0.4751	0.7353	0.8627	0.8039	0.6667	0.7069	0.7727
Zscore_PCC_Relief_12_LDA	0.718 [0.6460-0.7900]	0.0367	0.6309	0.517	0.4232	0.7108	0.9118	0.7549	0.6667	0.6937	0.7312
Zscore_PCC_Relief_12_AE	0.6745 [0.6007-0.7482]	0.0377	0.6323	0.573	0.3509	0.652	1.5	0.402	0.902	0.8039	0.6013
Zscore_PCC_Relief_12_RF	0.8031 [0.7422-0.8640]	0.0311	0.7662	0.375	0.5334	0.7549	0.7059	0.902	0.6078	0.697	0.8611
Zscore_PCC_Relief_12_LR	0.7643 [0.6983-0.8304]	0.0337	0.6904	0.4274	0.4673	0.7304	0.8333	0.8137	0.6471	0.6975	0.7765
Zscore_PCC_Relief_12_LRLasso	0.7773 [0.7130-0.8416]	0.0328	0.705	0.481	0.4511	0.7255	0.9804	0.7353	0.7157	0.7212	0.73
Zscore_PCC_Relief_12_AB	0.7543 [0.6867-0.8220]	0.0345	0.6943	0.5064	0.4422	0.7206	1.0686	0.6863	0.7549	0.7368	0.7064
Zscore_PCC_Relief_12_DT	0.6667 [0.6018-0.7315]	0.0331	0.7479	1	0.3339	0.6667	1.0588	0.6373	0.6961	0.6771	0.6574
Zscore_PCC_Relief_12_GP	0.7815 [0.7177-0.8453]	0.0326	0.7325	0.5112	0.472	0.7353	1.0784	0.6961	0.7745	0.7553	0.7182
Zscore_PCC_Relief_12_NB	0.5604 [0.4808-0.6399]	0.0406	0.5178	0.9985	0.182	0.5833	0.598	0.7843	0.3824	0.5594	0.6393
Zscore_PCC_Relief_13_SVM	0.7675 [0.7012-0.8338]	0.0338	0.6845	0.4751	0.472	0.7353	0.9216	0.7745	0.6961	0.7182	0.7553
Zscore_PCC_Relief_13_LDA	0.7321 [0.6615-0.8028]	0.036	0.6417	0.45	0.4429	0.7206	0.9118	0.7647	0.6765	0.7027	0.7419
Zscore_PCC_Relief_13_AE	0.7035 [0.6320-0.7749]	0.0364	0.6498	0.4942	0.3563	0.6765	0.8627	0.7451	0.6078	0.6552	0.7045
Zscore_PCC_Relief_13_RF	0.7972 [0.7360-0.8585]	0.0312	0.7678	0.355	0.5491	0.7598	0.6765	0.9216	0.598	0.6963	0.8841
Zscore_PCC_Relief_13_LR	0.7629 [0.6965-0.8293]	0.0339	0.6859	0.4191	0.4872	0.7402	0.8333	0.8235	0.6569	0.7059	0.7882
Zscore_PCC_Relief_13_LRLasso	0.7716 [0.7064-0.8369]	0.0333	0.6931	0.4646	0.481	0.7402	0.951	0.7647	0.7157	0.729	0.7526
Zscore_PCC_Relief_13_AB	0.7848 [0.7205-0.8491]	0.0328	0.7017	0.4839	0.5284	0.75	0.6765	0.9118	0.5882	0.6889	0.8696
Zscore_PCC_Relief_13_DT	0.6667 [0.6017-0.7317]	0.0332	0.7492	1	0.3334	0.6667	1.0196	0.6569	0.6765	0.67	0.6635
Zscore_PCC_Relief_13_GP	0.787 [0.7240-0.8500]	0.0321	0.75	0.5103	0.5029	0.75	1.1078	0.6961	0.8039	0.7802	0.7257
Zscore_PCC_Relief_13_NB	0.5548 [0.4752-0.6344]	0.0406	0.5199	0.9957	0.2182	0.5882	0.4118	0.8824	0.2941	0.5556	0.7143
Zscore_PCC_Relief_14_SVM	0.7714 [0.7055-0.8373]	0.0336	0.6862	0.4328	0.4658	0.7304	0.8529	0.8039	0.6569	0.7009	0.7701
Zscore_PCC_Relief_14_LDA	0.7516 [0.6833-0.8199]	0.0349	0.6605	0.4196	0.4553	0.7255	0.8627	0.7941	0.6569	0.6983	0.7614
Zscore_PCC_Relief_14_AE	0.6626 [0.5879-0.7374]	0.0381	0.6085	0.6349	0.2946	0.6471	1.0588	0.6176	0.6765	0.6562	0.6389
Zscore_PCC_Relief_14_RF	0.8108 [0.7519-0.8697]	0.0301	0.7886	0.495	0.5102	0.7549	0.9608	0.7745	0.7353	0.7453	0.7653
Zscore_PCC_Relief_14_LR	0.7646 [0.6983-0.8309]	0.0338	0.6879	0.481	0.4518	0.7255	0.9412	0.7549	0.6961	0.713	0.7396
Zscore_PCC_Relief_14_LRLasso	0.776 [0.7112-0.8407]	0.033	0.6995	0.4888	0.4906	0.7451	0.9608	0.7647	0.7255	0.7358	0.7551
Zscore_PCC_Relief_14_AB	0.7721 [0.7064-0.8378]	0.0335	0.6832	0.5047	0.4626	0.7304	1.0882	0.6863	0.7745	0.7527	0.7117
Zscore_PCC_Relief_14_DT	0.6863 [0.6225-0.7500]	0.0325	0.7628	1	0.3737	0.6863	1.0784	0.6471	0.7255	0.7021	0.6727
Zscore_PCC_Relief_14_GP	0.7912 [0.7297-0.8528]	0.0314	0.7678	0.5069	0.4936	0.7451	1.1176	0.6863	0.8039	0.7778	0.7193
Zscore_PCC_Relief_14_NB	0.5662 [0.4871-0.6453]	0.0404	0.5586	0.9936	0.2222	0.5882	0.3922	0.8922	0.2843	0.5549	0.725
Zscore_PCC_Relief_15_SVM	0.7776 [0.7123-0.8430]	0.0334	0.6937	0.4505	0.4739	0.7353	0.8824	0.7941	0.6765	0.7105	0.7667
Zscore_PCC_Relief_15_LDA	0.7713 [0.7046-0.8381]	0.034	0.6785	0.5289	0.471	0.7353	1.0392	0.7157	0.7549	0.7449	0.7264
Zscore_PCC_Relief_15_AE	0.5685 [0.4892-0.6479]	0.0405	0.5151	0.5399	0.1479	0.5735	0.8922	0.6275	0.5196	0.5664	0.5824
Zscore_PCC_Relief_15_RF	0.8105 [0.7514-0.8696]	0.0301	0.7888	0.43	0.5162	0.7549	0.8431	0.8333	0.6765	0.7203	0.8023
Zscore_PCC_Relief_15_LR	0.7626 [0.6960-0.8292]	0.034	0.6871	0.4056	0.4799	0.7353	0.8039	0.8333	0.6373	0.6967	0.7927
Zscore_PCC_Relief_15_LRLasso	0.7811 [0.7166-0.8456]	0.0329	0.7017	0.4435	0.5029	0.75	0.8922	0.8039	0.6961	0.7257	0.7802
Zscore_PCC_Relief_15_AB	0.7479 [0.6806-0.8152]	0.0343	0.7108	0.509	0.4368	0.7157	1.1569	0.6373	0.7941	0.7558	0.6864
Zscore_PCC_Relief_15_DT	0.6471 [0.5812-0.7129]	0.0336	0.7363	1	0.2942	0.6471	0.9804	0.6569	0.6373	0.6442	0.65
Zscore_PCC_Relief_15_GP	0.7822 [0.7188-0.8455]	0.0323	0.7626	0.5158	0.4963	0.7451	1.1569	0.6667	0.8235	0.7907	0.7119
Zscore_PCC_Relief_15_NB	0.5574 [0.4780-0.6367]	0.0405	0.5208	0.9939	0.2119	0.5833	0.3824	0.8922	0.2745	0.5515	0.7179
Zscore_PCC_Relief_16_SVM	0.7593 [0.6918-0.8268]	0.0344	0.6888	0.4944	0.4714	0.7353	0.9412	0.7647	0.7059	0.7222	0.75
Zscore_PCC_Relief_16_LDA	0.7453 [0.6757-0.8149]	0.0355	0.6542	0.5323	0.4608	0.7304	0.9902	0.7353	0.7255	0.7282	0.7327
Zscore_PCC_Relief_16_AE	0.6474 [0.5721-0.7228]	0.0384	0.6185	0.5033	0.2553	0.6275	1.0588	0.598	0.6569	0.6354	0.6204
Zscore_PCC_Relief_16_RF	0.8084 [0.7488-0.8680]	0.0304	0.7913	0.465	0.5239	0.7598	0.8725	0.8235	0.6961	0.7304	0.7978
Zscore_PCC_Relief_16_LR	0.7654 [0.6988-0.8319]	0.0339	0.6932	0.4347	0.4751	0.7353	0.8627	0.8039	0.6667	0.7069	0.7727
Zscore_PCC_Relief_16_LRLasso	0.7782 [0.7131-0.8432]	0.0332	0.7027	0.4732	0.4815	0.7402	0.9314	0.7745	0.7059	0.7248	0.7579
Zscore_PCC_Relief_16_AB	0.789 [0.7270-0.8511]	0.0317	0.7439	0.4913	0.4765	0.7353	0.8431	0.8137	0.6569	0.7034	0.7791

Zscore_PCC_Relief_16_DT	0.6716 [0.6068-0.7363]	0.033	0.7541	1	0.3432	0.6716	0.9902	0.6765	0.6667	0.6699	0.6733
Zscore_PCC_Relief_16_GP	0.793 [0.7311-0.8549]	0.0316	0.7672	0.5069	0.5413	0.7696	1.0882	0.7255	0.8137	0.7957	0.7477
Zscore_PCC_Relief_16_NB	0.5489 [0.4694-0.6284]	0.0406	0.5111	0.9976	0.1773	0.5735	0.4412	0.8529	0.2941	0.5472	0.6667
Zscore_PCC_Relief_17_SVM	0.7667 [0.7002-0.8333]	0.0339	0.7047	0.4703	0.4917	0.7451	0.9216	0.7843	0.7059	0.7273	0.766
Zscore_PCC_Relief_17_LDA	0.7477 [0.6785-0.8169]	0.0353	0.658	0.5296	0.461	0.7304	0.9706	0.7451	0.7157	0.7238	0.7374
Zscore_PCC_Relief_17_AE	0.7072 [0.6360-0.7784]	0.0363	0.6567	0.5258	0.3492	0.6716	0.8137	0.7647	0.5784	0.6446	0.7108
Zscore_PCC_Relief_17_RF	0.8077 [0.7482-0.8671]	0.0303	0.782	0.415	0.5199	0.7549	0.8039	0.8529	0.6569	0.7131	0.8171
Zscore_PCC_Relief_17_LR	0.7645 [0.6980-0.8310]	0.0339	0.7032	0.4122	0.4999	0.7451	0.8039	0.8431	0.6471	0.7049	0.8049
Zscore_PCC_Relief_17_LRLasso	0.7769 [0.7117-0.8421]	0.0333	0.704	0.3961	0.4949	0.7451	0.8627	0.8137	0.6765	0.7155	0.7841
Zscore_PCC_Relief_17_AB	0.7664 [0.7015-0.8314]	0.0331	0.7131	0.4949	0.446	0.7206	0.8529	0.7941	0.6471	0.6923	0.7586
Zscore_PCC_Relief_17_DT	0.7059 [0.6431-0.7687]	0.0321	0.779	1	0.4118	0.7059	1.0196	0.6961	0.7157	0.71	0.7019
Zscore_PCC_Relief_17_GP	0.7945 [0.7327-0.8562]	0.0315	0.7713	0.5008	0.5298	0.7647	1.0392	0.7451	0.7843	0.7755	0.7547
Zscore_PCC_Relief_17_NB	0.5524 [0.4730-0.6318]	0.0405	0.5306	0.9997	0.1849	0.5784	0.4706	0.8431	0.3137	0.5513	0.6667
Zscore_PCC_Relief_18_SVM	0.7644 [0.6975-0.8313]	0.0341	0.6993	0.5313	0.472	0.7353	1.0784	0.6961	0.7745	0.7553	0.7182
Zscore_PCC_Relief_18_LDA	0.7442 [0.6744-0.8141]	0.0356	0.654	0.3912	0.4635	0.7304	0.8922	0.7843	0.6765	0.708	0.7582
Zscore_PCC_Relief_18_AE	0.595 [0.5172-0.6727]	0.0397	0.5671	0.4757	0.1838	0.5833	0.5784	0.7941	0.3725	0.5586	0.6441
Zscore_PCC_Relief_18_RF	0.8026 [0.7420-0.8633]	0.031	0.7754	0.45	0.5134	0.7549	0.8824	0.8137	0.6961	0.7281	0.7889
Zscore_PCC_Relief_18_LR	0.7671 [0.7010-0.8332]	0.0337	0.706	0.4334	0.4843	0.7402	0.8725	0.8039	0.6765	0.713	0.7753
Zscore_PCC_Relief_18_LRLasso	0.7818 [0.7171-0.8465]	0.033	0.7083	0.612	0.4949	0.7451	1.1373	0.6765	0.8137	0.7841	0.7155
Zscore_PCC_Relief_18_AB	0.766 [0.7012-0.8307]	0.0331	0.7071	0.496	0.4029	0.701	0.9314	0.7353	0.6667	0.6881	0.7158
Zscore_PCC_Relief_18_DT	0.7059 [0.6430-0.7687]	0.0321	0.7794	1	0.4118	0.7059	1	0.7059	0.7059	0.7059	0.7059
Zscore_PCC_Relief_18_GP	0.7858 [0.7225-0.8490]	0.0323	0.7604	0.5024	0.5295	0.7647	1.0196	0.7549	0.7745	0.77	0.7596
Zscore_PCC_Relief_18_NB	0.5597 [0.4806-0.6388]	0.0404	0.5446	0.9997	0.1746	0.5735	0.4608	0.8431	0.3039	0.5478	0.6596
Zscore_PCC_Relief_19_SVM	0.7665 [0.7006-0.8324]	0.0336	0.7057	0.5221	0.471	0.7353	1.0392	0.7157	0.7549	0.7449	0.7264
Zscore_PCC_Relief_19_LDA	0.7492 [0.6800-0.8184]	0.0353	0.6571	0.4251	0.4532	0.7255	0.902	0.7745	0.6765	0.7054	0.75
Zscore_PCC_Relief_19_AE	0.5797 [0.5009-0.6585]	0.0402	0.5383	0.4623	0.1685	0.5833	0.8529	0.6569	0.5098	0.5726	0.5977
Zscore_PCC_Relief_19_RF	0.8106 [0.7515-0.8698]	0.0302	0.7872	0.38	0.542	0.7598	0.7157	0.902	0.6176	0.7023	0.863
Zscore_PCC_Relief_19_LR	0.7743 [0.7093-0.8393]	0.0332	0.7149	0.4184	0.4963	0.7451	0.8431	0.8235	0.6667	0.7119	0.7907
Zscore_PCC_Relief_19_LRLasso	0.7783 [0.7136-0.8429]	0.033	0.7075	0.5332	0.471	0.7353	1.0392	0.7157	0.7549	0.7449	0.7264
Zscore_PCC_Relief_19_AB	0.7842 [0.7215-0.8469]	0.032	0.7266	0.5101	0.4635	0.7304	1.1078	0.6765	0.7843	0.7582	0.708
Zscore_PCC_Relief_19_DT	0.701 [0.6379-0.7641]	0.0322	0.7765	1	0.4021	0.701	0.9706	0.7157	0.6863	0.6952	0.7071
Zscore_PCC_Relief_19_GP	0.7802 [0.7163-0.8441]	0.0326	0.7507	0.5003	0.5099	0.7549	1.0196	0.7451	0.7647	0.76	0.75
Zscore_PCC_Relief_19_NB	0.5563 [0.4771-0.6355]	0.0404	0.5393	0.9998	0.1746	0.5735	0.4608	0.8431	0.3039	0.5478	0.6596
Zscore_PCC_Relief_20_SVM	0.7606 [0.6934-0.8277]	0.0343	0.6997	0.4644	0.4739	0.7353	0.8824	0.7941	0.6765	0.7105	0.7667
Zscore_PCC_Relief_20_LDA	0.7466 [0.6766-0.8166]	0.0357	0.6489	0.4081	0.4541	0.7255	0.8824	0.7843	0.6667	0.7018	0.7556
Zscore_PCC_Relief_20_AE	0.6413 [0.5643-0.7183]	0.0393	0.563	0.5128	0.3082	0.652	0.8333	0.7353	0.5686	0.6303	0.6824
Zscore_PCC_Relief_20_RF	0.807 [0.7475-0.8664]	0.0303	0.7798	0.38	0.5157	0.75	0.7549	0.8725	0.6275	0.7008	0.8312
Zscore_PCC_Relief_20_LR	0.7708 [0.7050-0.8365]	0.0336	0.7143	0.4243	0.5029	0.75	0.8922	0.8039	0.6961	0.7257	0.7802
Zscore_PCC_Relief_20_LRLasso	0.7769 [0.7120-0.8418]	0.0331	0.7066	0.5559	0.4714	0.7353	1.0588	0.7059	0.7647	0.75	0.7222
Zscore_PCC_Relief_20_AB	0.7751 [0.7116-0.8385]	0.0324	0.7276	0.506	0.4221	0.7108	1.049	0.6863	0.7353	0.7216	0.7009
Zscore_PCC_Relief_20_DT	0.7059 [0.6430-0.7687]	0.0321	0.7794	1	0.4118	0.7059	1	0.7059	0.7059	0.7059	0.7059
Zscore_PCC_Relief_20_GP	0.7839 [0.7213-0.8466]	0.032	0.7681	0.5089	0.4872	0.7402	1.1667	0.6569	0.8235	0.7882	0.7059
Zscore_PCC_Relief_20_NB	0.5552 [0.4760-0.6343]	0.0404	0.5439	0.9992	0.1729	0.5686	0.3922	0.8725	0.2647	0.5427	0.675
